# How likely is unmeasured confounding to explain meta-analysis-derived associations between alcohol, other substances, and mood-related conditions with HIV risk behaviors?

**DOI:** 10.1186/s12874-025-02490-9

**Published:** 2025-03-07

**Authors:** Prima Manandhar-Sasaki, Kaoon Francois Ban, Emma Richard, R. Scott Braithwaite, Ellen C. Caniglia

**Affiliations:** 1 Department of Population Health, New York University Grossman School of Medicine, New York, NY USA; 2 Perelman School of Medicine, University of Pennsylvania, Philadelphia, PA USA

**Keywords:** Meta-analysis, Unmeasured confounding, E-value, Proportion of meaningfully strong effects, HIV, Substance use

## Abstract

**Background:**

HIV transmission and disease progression may be driven by associations HIV risk behaviors have with a constellation of alcohol, other substance, and mood-related conditions (CASM). However, observational study-based measures of these associations are often prone to unmeasured confounding. While meta-analysis offers a systematic approach to summarize effect sizes across studies, the validity of these estimates can be compromised if similar biases exist across studies. Our analysis assesses the likelihood that unmeasured confounding explains meta-analysis-derived measures of association between CASM and HIV risk behaviors, and provides bias-adjusted estimates.

**Methods:**

We first conducted systematic reviews and meta-analyses to assess associations between CASM conditions and four HIV risk behaviors (medication non-adherence, unprotected sex, transactional sex, and multiple sexual partners). We then adjusted for potential unmeasured confounders using two methods designed for meta-analyses - Point Estimate and Proportion of Meaningfully Strong Effects methods. We selected “risk propensity” as an illustrative and potentially important unmeasured confounder based on the extant literature and mechanistic plausibility.

**Results:**

In analyses unadjusted for unmeasured confounding, 89% (24/27) of odds ratios (ORs) show strong evidence of a positive association, with alcohol use and stimulant use emerging as dominant risk factors for HIV risk behaviors. After adjusting for unmeasured confounding by risk propensity, 81% (22/27) of ORs still showed strong evidence of a positive association. Associations between mood-related conditions and HIV risk behaviors were more robust to unmeasured confounding than associations between alcohol use and other substance use and HIV risk behaviors.

**Conclusion:**

Despite residual confounding present in constituent studies, there remains strong evidence of associations between CASM and HIV risk behaviors as well as the clustered nature of CASM conditions. Our analysis provides an example of how to assess unmeasured confounding in meta-analysis-derived measures of association.

**Supplementary Information:**

The online version contains supplementary material available at 10.1186/s12874-025-02490-9.

## Background

Human immunodeficiency virus (HIV) remains a globally persistent public health issue, affecting an estimated 39 million people living with HIV (PLHIV) at the end of 2022, and leading to 630,000 deaths in 2022 from HIV-related causes [1]. While progress has been made to reach the 95-95-95 goals, shortcomings remain, as there were an estimated 1.3 million additional cases in 2022, 660,000 of which were reported in the African region [1]. A constellation of alcohol (e.g., alcohol use disorder), other substance (e.g., tobacco, opioid, and stimulant use disorders), and mood-related (e.g. depressive and generalized anxiety disorders and chronic pain) conditions (CASM), have a high co-prevalence with HIV and appear to be important facilitators for HIV transmission and disease progression. Alcohol users in sub-Saharan Africa (SSA) were found to have 1.61 (95% CI 1.44–1.80) times the odds of having HIV compared to non-alcohol users [2], and PLHIV in high-income countries have an estimated 1.2–2.4-fold greater prevalence of alcohol use disorder, major depression, generalized anxiety, and drug use disorder than that among non-PLHIV [3]. An estimated 11% and 26% of PLHIV and male PLHIV respectively in SSA smoke cigarettes [4], and 22% of PLHIV in Nigeria have anxiety disorders [5]. An estimated 31% (95% CI 26–38%) of PLHIV on antiretroviral therapy (ART) in SSA have significant depressive symptoms [6], and 18–81% of various PLHIV populations have major depression [7]. An estimated 25–90% [8], 50–70% [9], 16.8% [10], and 17–64% of PLHIV in the United States have chronic pain, are current smokers, have substance use disorders, and used an illicit drug in their lifetime, respectively. These comorbidities remain present across age, sex, and gender differences [3, 7, 11–14], and studies have suggested biological pathways that CASM contributes to HIV disease and progression [15–19].

While associations between CASM and HIV risk behaviors have been studied in depth, including medication non-adherence [20–32] and risky sexual behaviors [33–40], observational study-based measures are inherently prone to unmeasured confounding. That is, the influence of factors not included in an analysis that may be the true drivers of results observed. Observational study results risk such scenarios since only a finite group of infinite potential risk factors are hypothesized and measured. Furthermore, composite exposure measures (e.g. substance use) prevent single exposure association estimates, and variation in sensitivity and specificity of CASM screening and diagnosis tools and HIV risk behavior measurement scales add to the ambiguity of conclusions drawn by these observational studies. Despite these shortcomings, observational studies remain essential ways to understand public health phenomena, for ethical and practical reasons. While meta-analysis offers a systematic approach to summarize effect sizes across studies by aggregating data, the validity of these estimates can be compromised by bias from residual unmeasured confounding in observational constituent studies. Furthermore, confirmation bias has led to systematic inclusion of more common potential confounders while excluding others from routine consideration [41].

**Fig. 1:**
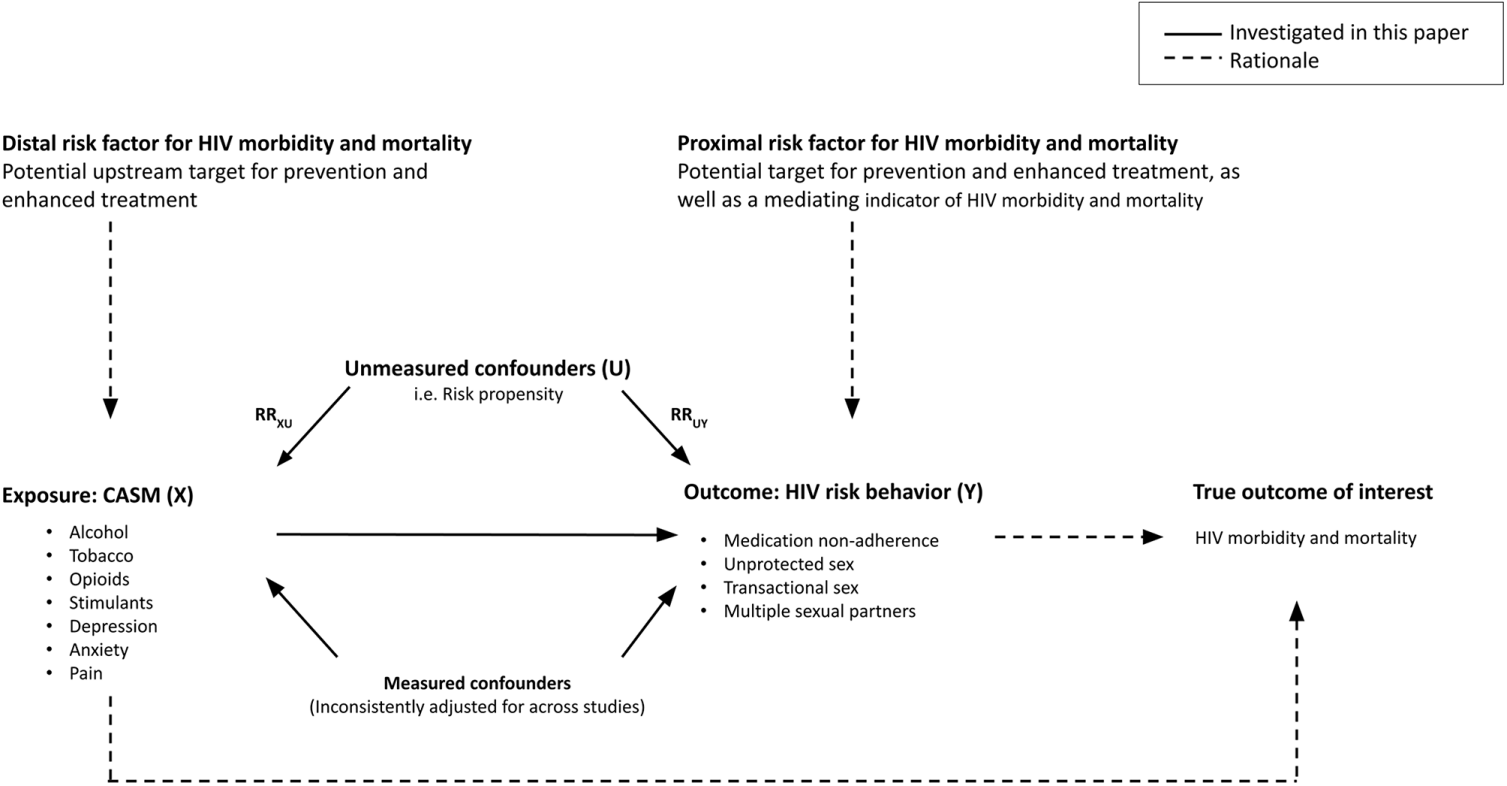
Conceptual diagram of CASM and HIV risk behavior

Our objective was to assess the likelihood that unmeasured confounding explains meta-analysis-derived measures of association between CASM and HIV risk behaviors, and to provide corresponding bias-adjusted estimates. Understanding the extent to which presumed causal associations between CASM and HIV risk behaviors are confounded can help identify integrative preventive strategies that complement the traditional HIV care cascade by addressing CASM (Fig. 1). We hypothesized that risk propensity (e.g., an increased preference to act in ways having elevated potential for negative consequences [42–45]) is an important unmeasured confounder between CASM conditions and HIV risk behaviors and selected it as our primary potential confounder of interest. Risk propensity has been associated with CASM [42, 43] and HIV risk behaviors [44, 45] and was not adjusted for in any constituent studies across our unadjusted analyses. We also hypothesized that the strength of unmeasured confounding by risk propensity would serve as a useful benchmark for the strength of other potential unmeasured confounders. We hypothesized that distrust of medical institutions (DIM) may also be an important confounder and selected it as a secondary potential confounder of interest, as it has been associated with both our exposures and outcomes of interest [57–60].

## Methods

First, we describe our analyses unadjusted for potential unmeasured confounders, conducted to derive summary estimates for associations between CASM conditions and HIV risk behaviors. Second, we describe how we analyzed these summary estimates for potential unmeasured confounding [46, 47].

### Analyses unadjusted for potential unmeasured confounding: systematic reviews and meta-analyses

We carried out 28 systematic reviews and performed corresponding meta-analyses to estimate the association between seven CASM (alcohol, depression, anxiety, pain, tobacco, opioids, stimulants) and four HIV risk behaviors (medication non-adherence, unprotected sex, transactional sex, and multiple sexual partners) (Supplementary Box 1; Figures S1-S26; Supplementary Box 2; Figures S27-S52). Our recently published meta-analysis characterizing the association between stimulants and HIV risk behaviors describes the methodology in detail and is reported elsewhere [48]. Briefly, we searched the PubMed database between July 2019-January 2021, filtering for English-language and human studies with no restrictions on setting, population, study design, or year. A single reviewer viewed abstracts from all search results and reviewed in full studies meeting inclusion criteria. Inclusion criteria required (1) the exposure of interest with a comparison group, (2) the outcome of interest, and (3) an odds ratio (OR) with corresponding 95% CI or other data from which ORs could be ascertained. To prevent overrepresentation of any study, one measure of association was used per constituent study. We used adjusted estimates when available. If multiple population groups or variations of exposure or outcome were presented, a pooled OR and 95% CI estimate was calculated [49]. If pooling was not possible, a series of decision rules was used: (1) we focused on the subgroup most commonly reported across all other constituent studies, the timeframe with the highest likelihood of exposure preceding the outcome, and the highest-level exposure and/or outcome stratum; (2) we prioritized an unadjusted OR including all subgroups over any particular subgroup-adjusted OR; (3) we harmonized directionalities (e.g., protected sex rather than unprotected sex) [49]; and (4) we excluded studies with composite exposure or outcome measures that might misclassify the exposure or dilute its effect.

*Medication non-adherence* refers to non-adherence to medication for HIV and/or hepatitis C virus (HCV), and no restrictions were placed on reporting method (e.g., self-reported adherence, visual analog scales, MEMs caps). *Unprotected sex* refers to condomless sex and includes all relevant measures (e.g., inconsistent condom use, condomless sex within specified recall times). *Transactional sex* refers to measures of sexual activity in which money or gifts were exchanged for sexual acts; we aggregated data on “buyers” and “sellers.” *Multiple sexual partners* refers to having multiple concurrent or subsequent sexual partners, and we considered different magnitudes and recall times. Exposure definitions were used as per individual study.

Our 28 systematic reviews yielded 27 measures of association (there were no eligible studies associating pain and transactional sex), 26 of which included ≥ 2 studies and therefore were amenable to meta-analyses (only one eligible study associated pain and multiple sexual partners). Random effects meta-analyses were conducted in RStudio, Version 1.3.1093, and Stata/IC, Version 15.1. We assessed heterogeneity in each meta-analysis by I-squared, tau-squared (τ²), and the Q-statistic.

### Analyses adjusted for potential unmeasured confounding

**Fig. 2:**
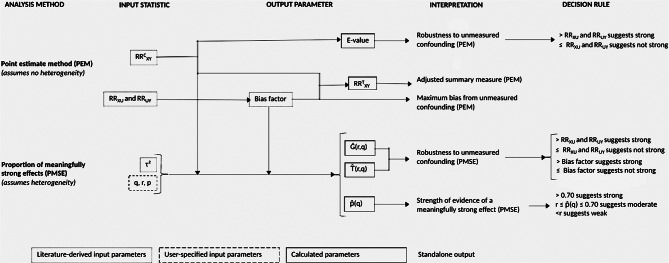
Parameter map for sensitivity analyses accounting for potential unmeasured confounding. *Literature-derived input parameters: ***RR**^**C**^_**XY**_: unadjusted meta-analysis-derived risk ratio (RR), presumed to have unmeasured confounding; **RR**^**T**^_**XY**_: true RR estimate, corrected for presumed unmeasured confounding; **RR**_**XU**_: RR between the exposure and U; **RR**_**UY**_: RR between U and the outcome; **τ²**: heterogeneity between studies. *User-specified input parameters: ***q**: minimum threshold (RR scale) of a meaningfully strong effect; **r**: the minimum proportion of constituent studies with true effects above q required to indicate moderate to strong evidence of an effect; **p**: proportion of heterogeneity (τ²) due to variation in confounding bias. *Output parameters:*** E-value**: Minimum confounding strength (risk ratio (RR) scale) by an unmeasured confounder that could explain RR^C^_XY_); **Bias factor**: maximum amount the unmeasured confounder could impact RR^C^_XY_, derived from RR_XU_ and RR_UY_, applied to RR^C^_XY_ to calculate RR^T^_XY_; **p̂(q)**: the proportion of studies that have scientifically meaningful strong effects (RR > q); **Ĝ(r**,** q)**: the minimum confounding strength required to lower p̂ to < r; ${\rm{\hat T}}$**(r**,** q)**: the minimum bias factor required to lower p̂ to < r

To assess how robust our meta-analysis-derived measures of association were to unmeasured confounding, we used the Point Estimate method and the Proportion of Meaningfully Strong Effects method (PMSE) [46, 47, 50–54]. The Point Estimate method is most suitable for single studies or for meta-analyses with low or no heterogeneity (e.g., τ² = 0), whereas PMSE is most suitable for meta-analyses with greater heterogeneity, albeit with a requirement of ≥ 10 studies. Neither approach requires specifying the prevalence of the unmeasured confounder. Both approaches allow interactions between the exposure (X), unmeasured confounder (U), and outcome (Y) [46, 47, 52, 54], and require the assumption that unmeasured confounders of interest are not completely collinear with any variables controlled for in constituent studies. To employ these methods, ORs were converted to risk ratios (RR) [54] using the square root transformation [55]. Figure 2 illustrates the interdependence of methods and parameters, and Table 1 lists key inputs used.

**Table 1:** Input parameters used in sensitivity analyses

Input parameter	Value	Reference
*All sensitivity analyses*		
Meta-analysis pooled risk ratios of CASM with HIV risk behaviors	Tables 2 and 3 (Figure S53 for secondary analysis)	Figures S27-S52
RR_XU_	1.54 (ATOS^1^ and RP^2^) 1.02 (DAP^3^ and RP)	[42, 43]
RR_UY_	1.40 (RP and medication non-adherence) 1.71 (RP and unprotected sex, transactional sex, multiple sexual partners)	[44, 45]
*Proportion of Meaningfully Strong Effects*		
Estimated heterogeneity (τ²)	τ² from meta-analysis statistical output	Table S1
Mean bias factor across studies	Tables 2 and 3 (single bias factor per meta-analysis assuming generalizable)	Corresponding RR_XU_ and RR_UY_ values
Proportion of heterogeneity (τ²) due to variation in confounding bias	0.80 (assumed high heterogeneity of bias across studies)	[53]
Threshold (q) for scientifically meaningfully strong effect size	1.10; 0.90	[46, 47]
Minimum proportion of constituent studies with true effects above q deemed to indicate moderate to strong evidence of an effect (r)	0.20 if 10 ≤ k ≤ 15 0.10 if k > 15	[47]
Tail	Above for q = 1.10 Below for q = 0.90	RR > 1 indicates causative effect, RR < 1 indicates protective effect

^1^ Alcohol, tobacco, opioids, and stimulants

^2^ Risk propensity

^3^ Depression, anxiety, and pain

**Table 2:** Assessment of unmeasured confounding among meta-analyses assessed by Point Estimate sensitivity analysis only

Exposure	Outcome	Pooled RR (95% CI)^1^	E-value^2^ (95% CI LL)	Bias factor^3^	Bias-adjusted RR (95% CI)
Alcohol	Non-adherence	1.40 (1.30–1.50)	2.15 (1.93)	1.11	1.26 (1.17–1.35)
Tobacco	Non-adherence	1.28 (1.20–1.38)	1.89 (1.68)	1.11	1.16 (1.08–1.24)
Stimulants	Non-adherence	1.59 (1.44–1.75)	2.55 (2.23)	1.11	1.43 (1.29–1.58)
Depression	Non-adherence	1.52 (1.35–1.72)	2.42 (2.04)	1.01	1.51 (1.34–1.71)
Anxiety	Non-adherence	1.44 (1.24–1.68)	2.24 (1.78)	1.01	1.43 (1.23–1.67)
Pain	Non-adherence	1.20 (1.08–1.34)	1.70 (1.38)	1.01	1.20 (1.08–1.33)
Pain	Unprotected sex	1.32 (1.08–1.62)	1.97 (1.37)	1.01	1.31 (1.07–1.61)
Alcohol	Transactional sex	1.54 (1.4–1.68)	2.44 (2.16)	1.17	1.31 (1.20–1.44)
Stimulants	Transactional sex	1.73 (1.58–1.89)	2.85 (2.54)	1.17	1.48 (1.35–1.62)
Depression	Transactional sex	1.32 (1.22–1.43)	1.98 (1.74)	1.01	1.31 (1.21–1.42)
Anxiety	Transactional sex	1.22 (1.08–1.38)	1.73 (1.37)	1.01	1.21 (1.07–1.37)
Tobacco	Multiple sexual partners	1.53 (1.42–1.64)	2.43 (2.20)	1.17	1.31 (1.22–1.40)
Opioids	Multiple sexual partners	1.14 (0.93–1.41)	1.55 (1.00)	1.17	0.98 (0.79–1.21)
Anxiety	Multiple sexual partners	0.97 (0.81–1.16)	1.21 (1.00)	1.01	0.98 (0.82–1.17)
Pain^4^	Multiple sexual partners	1.30 (1.10–1.55)	1.93 (1.42)	1.01	1.29 (1.09–1.54)

^1^ Converted from pooled ORs of Fig. 3

^2^ E-value: quantifies how strongly an unmeasured confounder would need to be associated with the exposure (minimum risk ratio RR_XU_) and outcome (minimum risk ratio RR_UY_) to fully explain the observed association (RR_XY_)

^3^ Bias factor: quantifies the maximum amount by which an unmeasured confounder could alter RR^T^_XY_

^4^ Single study

**Table 3:** Assessment of unmeasured confounding among meta-analyses assessed by Point Estimate and PMSE sensitivity analyses

Exposure	Outcome	Pooled RR (95% CI)^1^	E-value^2^ (95% CI LL)	Bias factor^3^	Bias-adjusted RR (95% CI)	p̂(q)^4^	${\rm{\hat T}}$(*r*, q)^5^	Ĝ(*r*, q)^6^
Opioids	Non-adherence	1.28 (1.16–1.42)	1.88 (1.60)	1.11	1.15 (1.05–1.28)	0.702	1.254	1.819
Alcohol	Unprotected sex	1.36 (1.26–1.46)	2.05 (1.83)	1.17	1.16 (1.07–1.25)	0.67	1.452	2.263
Tobacco	Unprotected sex	1.24 (1.12–1.37)	1.79 (1.48)	1.17	1.06 (0.96–1.17)	0.429	1.469	2.299
Opioids	Unprotected sex	1.10 (0.89–1.37)	1.44 (1.00)	1.17	0.94 (0.76–1.17)	0.315	1.315	1.959
Stimulants	Unprotected sex	1.44 (1.37–1.52)	2.24 (2.08)	1.17	1.23 (1.17–1.30)	0.786	1.569	2.514
Depression	Unprotected sex	1.24 (1.10–1.41)	1.80 (1.44)	1.01	1.23 (1.10–1.40)	0.769	1.279	1.875
Anxiety	Unprotected sex	1.19 (1.08–1.32)	1.67 (1.38)	1.01	1.18 (1.07–1.30)	0.688	1.294	1.912
Tobacco	Transactional sex	1.75 (1.32–2.3)	2.89 (1.98)	1.17	1.49 (1.13–1.97)	0.791	2.190	3.805
Opioids	Transactional sex	1.39 (1.1–1.76)	2.12 (1.43)	1.17	1.18 (0.94–1.51)	0.588	1.691	2.773
Alcohol	Multiple sexual partners	1.40 (1.27–1.53)	2.14 (1.86)	1.17	1.19 (1.09–1.31)	0.748	1.415	2.181
Stimulants	Multiple sexual partners	1.64 (1.43–1.89)	2.66 (2.21)	1.17	1.40 (1.22–1.61)	0.831	2.062	3.541
Depression	Multiple sexual partners	1.18 (1.07–1.31)	1.65 (1.34)	1.01	1.17 (1.06–1.30)	0.628	1.359	2.057

^1^ Converted from pooled ORs of Fig. 3

^2^ E-value: quantifies how strongly an unmeasured confounder would need to be associated with the exposure (minimum risk ratio RR_XU_) and outcome (minimum risk ratio RR_UY_) to fully explain the observed association (RR_XY_)

^3^ Bias factor: quantifies the maximum amount by which an unmeasured confounder could alter RR^T^_XY_

^4^ ${\rm{\hat p}}$(q): the proportion of studies in a meta-analysis likely to meet or exceed a user-specified threshold of meaningfully strong effect (q)

^5^ ${\rm{\hat T}}$(r, q): the minimum bias capable of reducing ${\rm{\hat p}}$(q) to less than r (analogous to the bias factor)

^6^ ${\rm{\hat G}}$(r, q): the minimum values of RR_UY_ and RR_XU_ capable of reducing p${\rm{\hat p}}$(q) to less than r (analogous to the E-value)

#### Point estimate method

The Point Estimate method entails calculating an E-value and an E-value 95% CI lower limit (LL) for associations of interest. Meta-analyses yield “g-values” rather than “E-values,” which pertain to single studies. Since they are mathematically equivalent to E-values, both terms are grouped together hereon. E-values quantify how strongly an unmeasured confounder would need to be associated with the exposure (minimum risk ratio RR_XU_) and outcome (minimum risk ratio RR_UY_) to fully explain the observed association (RR_XY_) [56], conditional on the measured covariates [54]. The E-value 95% CI lower limit (LL) quantifies the strength of the unmeasured confounder necessary for the 95% CI of the observed and likely confounded association to contain the null value. An E-value greater than both RR_XU_ and RR_UY_ indicates that unmeasured confounding cannot fully explain RR_XY_ [54]. First, the E-value and 95% CI LL were calculated using the formula:

E-value =

1 $$\text{R}\text{R}_\text{XY}\:+\:\text{S}\text{Q}\text{R}\text{T}\left(\text{R}\text{R}_\text{XY}\:\times\:\right(\text{R}\text{R}_\text{XY}\:-\hspace{0.17em}1\left)\right)$$

We used the inverse of the RR when the point estimate or 95% CI LL was below 1 to yield defined values, and 95% CI LLs were set to 1 if the 95% CI LL RR values were less than 1 [54], since an uncertainty range including the null already indicates sufficient association with the unmeasured confounder to explain RR_XY_.

We performed a sensitivity analysis to assess the relative magnitude of our E-values by using distrust in medical institutions (DIM) as an alternative unmeasured confounder [56]. To gauge if an E-value is “small” or “large,” it is useful to place it in context of RR_XU_ and RR_UY_ values corresponding to other, similar, unmeasured confounders [56]. This could indicate, for example, that an association with a seemingly large E-value is rather weak to confounding if associations between the exposure and outcome with a comparable potential confounder are relatively high-magnitude [51]. In accord with recommendations, we describe E-values that exceed “reference RRs” (RR_XU_ and RR_UY_ where the unmeasured confounder is DIM rather than risk propensity) as “very strong,” those within the range of reference RRs as “moderately strong,” and those less than reference RRs as “likely not strong” to unmeasured confounding.

Second, we estimated the lower bound estimate of the true measure of association (RR^T^_XY_) by calculating the bias-adjusted RR [47, 54], using a bias factor to “correct” the confounded measure of association (RR^c^_XY_). The bias factor quantifies the maximum amount by which an unmeasured confounder could alter RR^T^_XY_ [54] and is derived from RR_XU_ and RR_UY_ (Table 1). We assumed that RR_XU_ differs between two subcategories of CASM conditions (1: alcohol, tobacco, opioids, and stimulants (ATOS); 2: depression, anxiety, and pain (DAP)) because they had distinct clustering patterns, potentially representing differential associations with risk propensity. The bias factor was calculated by [54]:

2 $${\rm{B = }}\left( {{\rm{R}}{{\rm{R}}_{{\rm{UY}}}}{\rm{\times R}}{{\rm{R}}_{{\rm{XU}}}}} \right){\rm{ / }}\left( {{\rm{R}}{{\rm{R}}_{{\rm{UY}}}}{\rm{ + }}\left( {{\rm{R}}{{\rm{R}}_{{\rm{XU}}}}{\rm{ - 1}}} \right)} \right){\rm{ }}$$

The bias-adjusted RRs (and 95% CIs) were calculated by:

3 $$\:\text{R}\text{R}^\text{T}\:_\text{XY}\:\ge\:\:\text{R}\text{R}^\text{C}\:_\text{XY}\:/\:\text{B }\:(\text{i}\text{f}\:\text{R}\text{R}_\text{XY}\:>1)$$

$$\:\text{R}\text{R}^\text{T}\:_\text{XY}\:\le\:\:\text{R}\text{R}^\text{C}\:_\text{XY}\:\times\:\text{B }\:(\text{i}\text{f}\:\text{R}\text{R}_\text{XY}\:<1)$$

#### Proportion of meaningfully strong effects method (PMSE)

We used PMSE for meta-analyses with heterogeneity (τ² > 0), sufficient numbers (≥ 10 studies, k), and robust parametric confidence intervals (as determined by 0.15 ≤ p̂(q) ≤ 0.85; p̂(q) defined below). The PMSE method assumes a normal distribution of population effects across studies to account for (1) dispersion of individual study point estimates from a hypothesized “true effect” size and (2) statistical error commensurate with the number of studies. This is done to prevent using “statistically significant” pooled estimates that comprise few studies with meaningfully strong effects and/or effects in the opposite direction of association (52).

The PMSE first assessed the strength of evidence for each meta-analysis by estimating p̂(q): the proportion of studies in a meta-analysis likely to meet or exceed a user-specified threshold of meaningfully strong effect (q). We set q = 1.10 (risk ratio (RR)) since all meta-analyses analyzed had positive directions of association, and additionally assessed q = 0.90 to consider the possibility of an association in the opposite direction. For example, p̂(q = 1.10) = 0.2 means that 20% of individual studies in a given meta-analysis are likely to yield risk ratios of 1.10 or higher. Larger p̂(q) values indicate stronger evidence and suggest the meta-analysis is robust to unmeasured confounding (47). We used the following recommended criteria to categorize strength of evidence by p̂(q): p̂(q) < r (weak), r ≤ p̂(q) ≤ 0.70 (moderate), p̂(q) > 0.70 (strong), referencing values used in existing literature (47). Here, “r” is a user-specified threshold for the minimum proportion of true effects above q needed to suggest evidence for causation. Given the observational nature of included studies in our analysis, we interpret evidence for causation as evidence for association suggesting causation. Per pre-established guidelines (47), we set r equal to 0.20 for meta-analyses with < 15 studies and equal to 0.10 for meta-analyses with ≥ 15 studies. Thus, for a meta-analysis of 30 studies, p̂(q = 1.10) > 0.10 implies sufficient evidence of causation in RR^C^_XY_ since more than 10% of studies have true effect RRs above 1.10.

Second, for each meta-analysis, we used E-value analog parameters ${\rm{\hat T}}$(r, q) and Ĝ(r, q) to determine the magnitude of unmeasured confounding necessary to reduce p̂(q) below r (suggesting RR^C^_XY_ contains residual confounding). ${\rm{\hat T}}$(r, q), analogous to the bias factor used in single studies, reflects the minimum bias capable of reducing p̂(q) to less than r. Ĝ(r, q), analogous to the E-value used in single studies, indicates the minimum values of RR_UY_ and RR_XU_ capable of reducing p̂(q) to less than r. Put another way, a meta-analysis can be considered robust to unmeasured confounding if ${\rm{\hat T}}$(r, q) > the bias factor or Ĝ(r, q) > RR_XU_ and RR_UY_. Given *r* = 0.10, q = 1.10, and k ≥ 15, these conditions would indicate that more than 10% of studies have true RRs > 1.10 and that RR^C^_XY_ is a plausible estimate of RR^T^_XY_.

Analogous to the method used for the E-value, in order to gauge relative magnitude of Ĝ(r, q) values in the context of other potential unmeasured confounders, we describe Ĝ(r, q) that exceed the reference RRs (pertaining to DIM) as “very strong,” those within the range of reference RRs as “moderately strong,” and those less than reference RRs as “likely not strong” to unmeasured confounding. We apply the same categorizations for assessing the relative magnitude of ${\rm{\hat T}}$(r, q) but use reference bias factors (pertaining to DIM) in lieu of reference RRs.

The p̂(q) value, ${\rm{\hat T}}$(r, q), and Ĝ(r, q) values were calculated using the online tool accessible at: https://www.evalue-calculator.com/meta/ [47]. Results yielding p̂(q) < 0.15 or > 0.85 did not have robust parametric confidence intervals and are accordingly not reported.

### Secondary analyses

Our secondary analysis considered a subset of meta-analyses in which constituent studies adjust for ≥ 1 other CASM to explore the impact of measured confounding by other CASM (e.g., constituent studies examine the association between tobacco and medication non-adherence, adjusting for alcohol use disorder). This comprised 26 CASM-adjusted ORs (one association yielded no eligible studies and one association yielded no CASM-adjusted estimates) and 25 “meta-analysis sets” comparing the primary meta-analysis OR and the CASM-adjusted-subgroup meta-analysis OR (one association yielded a CASM-adjusted OR without a reference unadjusted OR) (Figure S53; Supplementary Box 3). We define notable confounding by other CASM as a ≥ 0.20 change-in-effect between ORs of a given meta-analysis set (adjusted value - unadjusted value). We discuss these results in more detail in Supplementary Boxes 3 and 4.

## Results

### Analyses unadjusted for potential unmeasured confounding: systematic reviews and meta-analyses

**Fig. 3:**
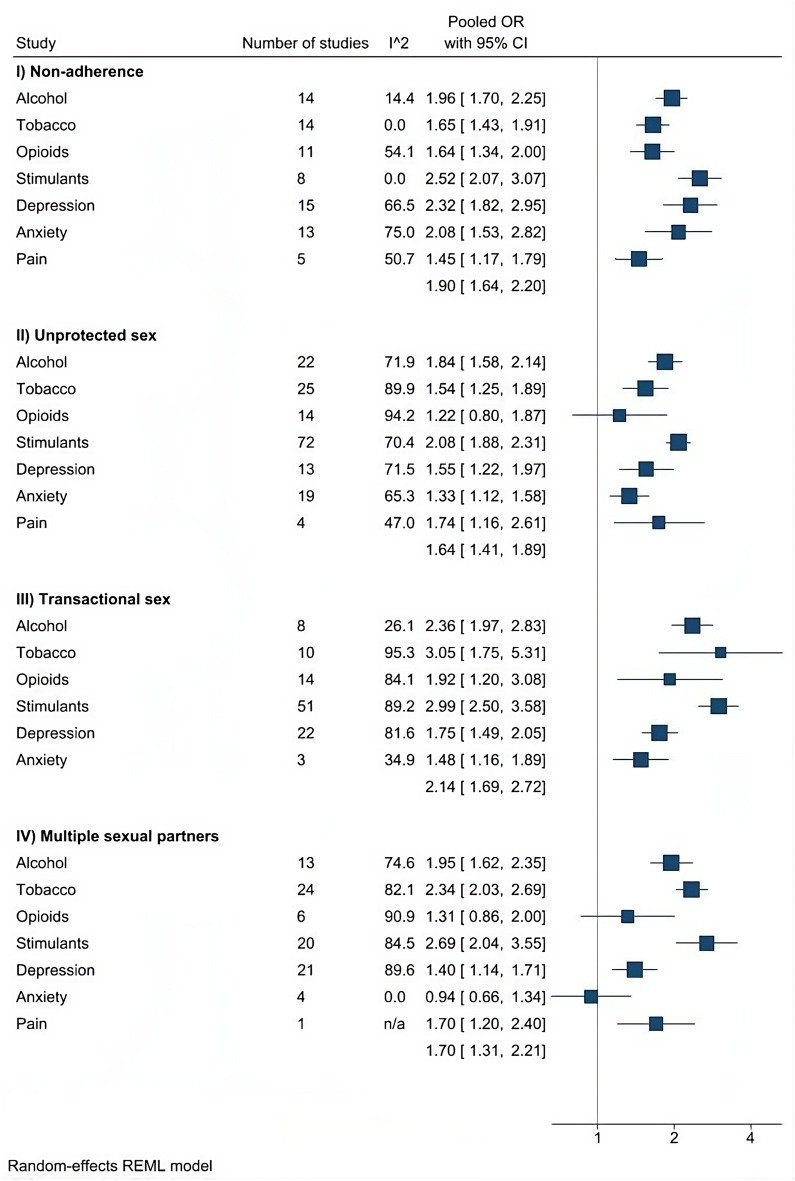
Meta-analysis pooled estimates of CASM-HIV risky behavior associations

Overall, 89% (24/27) of pooled ORs show strong evidence of a positive association with 95% CIs excluding the null, and varying levels of heterogeneity (Fig. 3). Pooled ORs of associations between CASM and medication non-adherence range from 1.45 (95% CI 1.17–1.79) to 2.52 (95% CI 2.07–3.07), with no confidence intervals spanning the null. Pooled ORs of associations between CASM and unprotected sex range from 1.22 (95% CI 0.80–1.87) to 2.08 (95% CI 1.88–2.31), with one of seven confidence intervals spanning the null. Pooled ORs for associations between CASM and transactional sex range from 1.48 (95% CI 1.16–1.89) to 3.05 (95% CI 1.75–5.31), with no confidence intervals spanning the null (there were no eligible studies for pain and transactional sex). Pooled ORs for associations between CASM and multiple sexual partners range from 0.94 (95% CI 0.66–1.35) to 2.69 (95% CI 2.04–3.55), with two of seven confidence intervals spanning the null. Alcohol, stimulants, depression, and anxiety have greater magnitudes of association with medication non-adherence compared to the other CASM, and alcohol and stimulants have greater magnitudes of association with unprotected sex compared to the other CASM. Alcohol, tobacco, and stimulants have greater magnitudes of association with transactional sex and multiple sexual partners compared to the other CASM (Fig. 3).

### Analyses adjusted for potential unmeasured confounding

#### Point estimate method

Twenty-seven associations were assessed by the E-value, among which 81% (22/27) are robust to unmeasured confounding by risk propensity, as indicated by E-values and/or E-value 95% CI LLs exceeding RR_XU_ and RR_UY_ (Tables 2 and 3). The pain and multiple sexual partners systematic review yielded a single relevant study for which the E-value exceeded RR_XU_ and RR_UY_. Among the non-robust associations, only the opioids and unprotected sex OR has an E-value (rather than the E-value 95% CI LL) exceeded by RR_XU_ and RR_UY_. E-values range from 1.21 (95% CI LL 1.00) to 2.89 (95% CI LL 1.98). 80% (4/5) of non-robust associations involved associations with ATOS exposures rather than DAP exposures, and all non-robust associations comprise sexual behavior outcomes rather than the medication non-adherence outcome. In the context of reference RRs of DIM (Table S4; Supplementary Box 5), our E-values appear relatively robust (more in the discussion).

Bias factors ranged from 1.01 (smaller amount of bias for associations with DAP exposures) to 1.11 and 1.17 (greater bias for associations with ATOS exposures (Table 1). Bias-adjusted RRs suggest the CASM with more robust associations are stimulants, depression, and anxiety for medication non-adherence, pain, stimulants and depression for unprotected sex, stimulants and tobacco for transactional sex, and stimulants, tobacco, and pain for multiple sexual partners (Tables 2 and 3).

#### Proportion of meaningfully strong effects method

Twelve meta-analyses had sufficient heterogeneity, comprised ≥ 10 studies, and were therefore assessed by the PMSE method. The strength of evidence for causation varies from moderate (r ≤ p̂(q) ≤ 0.70) to strong (p̂(q) > 0.70)) with p̂(q = 1.10) ranging from 0.315 (for opioids and unprotected sex) to 0.831 (for stimulants and multiple sexual partners) (Table 3). That is, 31.5–83.1% of individual studies in corresponding meta-analyses are likely to yield RRs ≥ 1.10. Of the 12 associations, 50% (6/12) have moderate evidence for causation (alcohol, anxiety, and tobacco, and opioids for unprotected sex; opioids for transactional sex, and depression for multiple sexual partners), and 50% (6/12) have strong evidence for causation (opioids for medication non-adherence, depression, and stimulants for unprotected sex; tobacco for transactional sex; stimulants and alcohol for multiple sexual partners) (Table 3). All meta-analyses demonstrate robustness to bias from unmeasured confounding by risk propensity, as all Ĝ(r, q) values exceed their respective RR_XU_ and RR_UY_ values, ranging from 1.819 (opioids and medication non-adherence) to 3.805 (tobacco and transactional sex) (Table 3). Furthermore, all ${\rm{\hat T}}$(r, q) values exceed their respective bias factor values, ranging from 1.254 (opioids and medication non-adherence) to 2.190 (tobacco and transactional sex). In the context of reference RRs and bias factors of DIM (Table S4; Supplementary Box 5; Table S5), our Ĝ(r, q) and ${\rm{\hat T}}$(r, q) values are relatively robust (more in the discussion).

The true association is not likely to be in the opposite direction of the observed association, as analyses altering q to RR 0.90 yield very low p̂(q) values (between 0 and 0.091) among these meta-analyses, except for tobacco and unprotected sex (0.214), opioids and unprotected sex (0.447), opioids and transactional sex (0.211).

### Secondary analyses

In secondary analyses of CASM-adjusted estimates (Figure S53; Supplementary Box 3; Tables S2-S3; Supplementary Box 4), we found that 73% (19/26) of pooled ORs show strong evidence of a positive association with 95% CIs excluding the null, and varying levels of heterogeneity. Among the 25 meta-analyses sets, 84% (21/25) of CASM-adjusted meta-analysis estimates have notable confounding by other CASM. Most of the notable shifts, 76% (16/21), occurred towards the null, suggesting positive confounding by other CASM. ATOS associations are more likely to have notable confounding by CASM than DAP associations (100% (15/15) versus 60% (6/10)), and among those with notable shifts, ATOS associations are also more likely to indicate positive confounding by other CASM (87% (13/15) versus 50% (3/6)). Among those with notable shifts, transactional sex, and multiple sexual partner associations are more likely than medication non-adherence and unprotected sex associations to indicate positive confounding by other CASM (100% (9/9) versus 57% (4/7) and 60% (3/5), respectively). In regard to E-values, 62% (16/26) are robust to unmeasured confounding by risk propensity, and 60% (6/10) of non-robust E-values comprised ATOS associations. E-values range from 1.00 (95% CI LL 1.00) to 2.97 (95% CI LL 2.24) (Tables S2-S3). Only two associations were assessed by PMSE, both showing strong evidence of causation (p̂(q) > 0.70) and robustness to unmeasured confounding by sufficiently large ${\rm{\hat T}}$(r, q) Ĝ(r, q) values (Tables S2-S3; Supplementary Box 4).

## Discussion

Our analyses unadjusted for potential unmeasured confounding demonstrate evidence for associations between CASM and HIV risk behaviors (Fig. 3), consistent with past findings [20–40]. Our findings add to the literature by increasing precision and generalizability and suggest that alcohol and stimulants are dominant risk factors for all investigated HIV risk behaviors. Our analyses accounting for potential unmeasured confounding by risk propensity indicate that most associations were robust to unmeasured confounding. Associations between ATOS exposures and HIV risk behaviors are less robust to unmeasured confounding than associations between DAP exposures and HIV risk behaviors. Bias-adjusted RRs reflect varying levels of bias by risk propensity, particular to exposure or outcome type. While some bias factors seem negligible in magnitude (e.g., for DAP and medication non-adherence), we present all bias-adjusted RRs to identify trends across CASM conditions and HIV risk behaviors.

Our sensitivity analyses designed to investigate unmeasured confounding in meta-analyses suggest that unmeasured confounding by risk propensity is unlikely to explain the meta-analyzed associations. Furthermore, no meta-analyses showed evidence of a “protective” effect (q = 0.90), suggesting it is unlikely that the true direction of association was negative. Though the Point Estimate method suggests ATOS associations are less robust to unmeasured confounding than DAP associations, PMSE results suggest all ATOS associations are robust to unmeasured confounding. In the PMSE analysis, all associations appear strong to unmeasured confounding, with 75% (9/12) of meta-analyzed studies comprising ATOS associations. This apparent “relative leniency” of PMSE, compared to the Point Estimate method, that deems any residual confounding insufficient to explain results is likely explained by its consideration for inter-study heterogeneous effects that are intrinsic to meta-analyses. Unlike the derivation of Point Estimate method parameters which solely use the central measure of association (RRs), the derivation of PMSE parameters include measures of variance and heterogeneity [47]. Accordingly, the presence of heterogeneity across our meta-analyses (Table S1) suggests more weight should be placed on the PMSE results, as they better characterize the heterogeneous nature of meta-analyzed data. Furthermore, use of meta-analysis-specific parameters facilitate comparison of our data with other literature.

Inferences from the sensitivity analyses hinge to an extent on the relative strength of the unmeasured confounder. To assess the robustness of our Point Estimate method findings, we used DIM as an alternative unmeasured confounder [56], comparing our E-values to corresponding exposure-covariable RRs (RR_XU_^ATOS-DIM^ or RR_XU_^DAP-DIM^) and covariable-outcome (RR_XU_^DIM-medication non-adherence^ or RR_XU_^DIM-risky sexual behavior^) (Table S4; Supplementary Box 5) [57–60]. DIM appears to influence the associations less than risk propensity, as all (27/27) E-values appear robust relative to DIM reference RRs. This suggests that our assessment that 81% of associations are robust may be conservative. Some differences emerge by outcome. For risky sexual behavior outcomes, unlike with risk propensity, we see that ATOS associations are generally more robust to DIM confounding than DAP associations. For medication non-adherence, all exposures except for pain are “very strong” and/or “moderately strong,” and pain is “likely not strong.” These differences illustrate the importance of the selection of the unmeasured confounder proxy used to interpret E-values. We believe risk propensity remains a suitable proxy for unmeasured confounding, as it covers behavioral factors transcending the medical sector.

The inferences from PMSE also hinge on the relative strength of the unmeasured confounder. There is almost perfect alignment between the relative assessment of Ĝ(r, q) and the relative assessment of E-values (as defined in methods) across meta-analyses (both compared to DIM E-values, Table S4, Supplementary Box 5). The only differences were depression and transactional sex and depression and multiple sexual partners (assessment was “likely not strong” by E-values and “very strong” by Ĝ(r, q) values). There is perfect alignment between the relative assessment of ${\rm{\hat T}}$(r, q) and the relative assessment of E-values (compared to DIM bias factors in Table S5 and DIM E-values in Table S4, respectively).

Our secondary analysis of CASM-adjusted estimates suggests that compared to DAP, ATOS associations are more sensitive to measured confounding (by CASM) and unmeasured confounding due to a greater prevalence of ATOS among meta-analysis sets suggesting notable, positive confounding. This suggests that ATOS has greater clustering with other CASM than DAP. Our results also indicate that associations between CASM and medication non-adherence associations are more sensitive to measured confounding (by other CASM factors) than associations between CASM and risky sexual behaviors are; however, they are less sensitive to unmeasured confounding than associations between CASM and risky sexual behaviors. This suggests that measures of association that adjust for other CASM may sufficiently account for residual confounding in CASM-medication non-adherence associations. Although the ATOS-DAP distinction was not seen by PMSE, this is likely reflective of its limited scope, as only two meta-analyses were included by this method in the secondary analysis. Our secondary analysis findings are slightly different when considered relative to DIM. First, CASM-adjusted meta-analyses have a lower prevalence of robust estimates (as determined by the E-value) at 73% (19/26), compared to 100% (27/27) in the primary analysis. While we hypothesized the opposite, that CASM adjustment increases robustness to unmeasured confounding, these results could indicate that non-CASM confounders influence our associations more than CASM confounders. Alternatively, or in combination, CASM-adjusted studies may account for non-CASM conditions less than studies that do not adjust for CASM. Second, we see a higher prevalence of robustness to unmeasured confounding relative to DIM across secondary analyses (73% (19/26) for DIM and 62% (16/26) for risk propensity), supporting previous findings that risk propensity appears to influence our associations more than DIM. Despite these differences, CASM-adjusted meta-analyses are still “very strong” to unmeasured confounding (Tables S2-S3; Supplementary Box 4; Table S4; Supplementary Box 5; Table S5) according to Ĝ(r, q) and ${\rm{\hat T}}$(r, q) values.

Our analyses were not without limitations. Our systematic review searches were limited to PubMed and English articles. Most constituent studies were cross-sectional and have potential for reverse causality, measurement error, selection bias, and publication bias [56]. Limiting inclusion of studies to those reporting ORs or convertible data may have introduced bias by requiring a dichotomous outcome but is not likely to affect findings. We did not perform risk of bias qualitative assessments for each meta-analysis, although we did assess the impact of CASM adjustment via secondary analysis. We had a single reviewer per meta-analysis and do not have a kappa test value of internal validity. However, a standardized data collection document and protocol were used. Decision rules used to standardize data collection across the meta-analyses have the potential to introduce bias compounded by publication bias (e.g., by choosing the mode population group or higher exposure strata to avoid overrepresentation of a study). Our secondary analysis-based interpretations on the presence and direction of measured confounding by other CASM are non-definitive, as they imperfectly assess confounding due to (1) the mutual inclusivity of CASM-adjusted studies in both primary and secondary analyses, (2) comparing studies in lieu of individuals for change-in-effect, and (3) a small study count for some secondary meta-analyses. Furthermore, we did not explore the possibility of unmeasured negative confounding that may bias results towards rather than away from the null effect. In such a case, the literature-based input value RR_UY_ would have the reverse direction of association with the outcome compared to the direction of association between the exposure and outcome. For example, if the RR between the exposure and outcome is positive, RR_UY_ would be negative. In this case, the E-values and PMSE-derived parameters would reflect the strength of unmeasured negative confounders in skewing effect estimates towards the null - that is, the extent to which they underestimate the true effect. For our point estimate analyses, associations with relatively high E-values may still have residual unmeasured confounding arising from other sources, such as the cross-sectional design of many constituent studies [61]. Interpretation of the E-value depends on the magnitude of difference between the exposed and unexposed groups, but this nuance was not assessed due to variation across constituent studies. We used one or two studies to estimate alternative RR_XU_, RR_UY_, and bias factors. However, this may be an oversimplification if these studies do not align with the specific study populations across all constituent studies they were applied to. It may be argued that potential unmeasured confounders other than risk propensity and DIM could be suitable candidates in this analysis. While our selection was based on plausibility, literature, and consideration to avoid colliders, alternative candidates could be applied to our illustrative analysis. As with the Point Estimate method, we used one or two studies to estimate alternative RR_XU_, RR_UY_, and bias factors for PMSE which may not capture the full plausible range of values. Despite its utility for quantitative assessment of unmeasured confounding in heterogeneous contexts, p̂(q) benchmark values and user-specified thresholds were somewhat arbitrary, though guideline- and literature-based to procure results compatible with other literature. 95% CIs were not available for interpretation in PMSE since the variance of τ² and the pooled point estimate were not available for each meta-analysis. We also only assessed a single threshold for a meaningful effect, RR 1.10, which could be a relatively low threshold. Only 42% (11/26) of studies met the criteria for PMSE, so this analysis was limited in scope. However, we reported E-values for all 27 meta-analyses and single studies to provide a uniform parameter for comparison. Although some constituent studies adjust for various potential confounders, we do not believe they adequately consider all unmeasured confounding and accordingly do not presume that our bias-adjusted RRs over-adjust for confounders.

## Conclusion

CASM affect a sizeable portion of PLHIV, with effects reverberating from the individual to society. The associations between CASM and HIV transmission and progression risk factors highlight prevention opportunities, especially in light of evidence for effective interventions that treat CASM among PLHIV [62]. In fact, our measures of association have been used to inform a model examining the potential health benefits of integrated screening strategies for CASM conditions among PLHIV, using upper bound estimates for residual confounding [63]. Our analyses accounting for potential unmeasured confounding by risk propensity suggest that alcohol and stimulants are dominant risk factors for all investigated HIV risk behaviors, highlighting potential high-impact targets for integrative intervention strategies to reduce HIV morbidity and mortality. Associations with ATOS exposures are more sensitive than associations with DAP exposures to unmeasured confounding and more likely to be positively confounded by other CASM, suggesting greater clustering of ATOS conditions with other CASM and underscoring the value of targeting alcohol and stimulants in integrative intervention strategies. Despite evidence of measured and unmeasured confounding, we demonstrate that most associations are sufficiently strong, with or without adjustment for bias.

## Electronic supplementary material

Below is the link to the electronic supplementary material.

Supplementary Material 1

## Data Availability

The supplementary file contains additional information on the systematic reviews and meta-analyses (search terms, PRISMA diagrams, constituent article reference list, meta-analysis forest plots) as well as the analyses adjusting for potential unmeasured confounding (meta-analysis heterogeneity values, full secondary analysis, and measures of association, E-values, and bias factors for the comparable potential unmeasured confounder).

## Abbreviations

HIV: Human immunodeficiency virus
PLHIV: People living with HIV
CASM: Constellation of alcohol (e.g., alcohol use disorder), other substance (e.g., tobacco, opioid, and stimulant use disorders), and mood-related (e.g. depressive and generalized anxiety disorders and chronic pain) conditions
ATOS: Alcohol, tobacco, opioids, and stimulants
DAP: Depression, anxiety, and pain
DIM: Distrust of medical institutions
PMSE: Proportion of Meaningfully Strong Effects method
