# Supplementary material for: How likely is unmeasured confounding to explain meta-analysis-derived associations between alcohol, other substances, and mood-related conditions with HIV risk behaviors?
Source: BMC Med Res Methodol. 2025 Mar 7;25:62. doi: 10.1186/s12874-025-02490-9 (PMC11887180; doi:10.1186/s12874-025-02490-9)
Supplement: Supplementary file 1 — Supplementary Material 1 [file 12874_2025_2490_MOESM1_ESM.docx]

**Supplementary File**

Table of Contents

[Supplementary Box 1: Systematic review search terms 3](#_gjdgxs)

[Figure S1: Systematic review PRISMA diagram: alcohol and medication non-adherence 7](#_30j0zll)

[Figure S2: Systematic review PRISMA diagram: depression and medication non-adherence 8](#_bf5a3ftwpqfb)

[Figure S3: Systematic review PRISMA diagram: anxiety and medication non-adherence 8](#_gu20oiygxwp7)

[Figure S4: Systematic review PRISMA diagram: pain and medication non-adherence 9](#_o949i276q21p)

[Figure S5: Systematic review PRISMA diagram: tobacco and medication non-adherence 9](#_4axsl3rms6fk)

[Figure S6: Systematic review PRISMA diagram: opioids and medication non-adherence 9](#_7ycnrjo5eqho)

[Figure S7: Systematic review PRISMA diagram: stimulants and medication non-adherence 10](#_uy1gszoalp2w)

[Figure S8: Systematic review PRISMA diagram: alcohol and unprotected sex 11](#_g571hzgw3fq4)

[Figure S9: Systematic review PRISMA diagram: depression and unprotected sex 11](#_pc0j2ylbhj83)

[Figure S10: Systematic review PRISMA diagram: anxiety and unprotected sex 12](#_sqv4r7fguviz)

[Figure S11: Systematic review PRISMA diagram: pain and unprotected sex 12](#_4hjsh4pn1e9a)

[Figure S12: Systematic review PRISMA diagram: tobacco and unprotected sex 13](#_y0h0nmxpcct8)

[Figure S13: Systematic review PRISMA diagram: opioids and unprotected sex 13](#_5mcsnbu7d7mj)

[Figure S14: Systematic review PRISMA diagram: stimulants and unprotected sex 14](#_okj3n6ebk3y)

[Figure S15: Systematic review PRISMA diagram: alcohol and transactional sex 14](#_thysxb7e1k64)

[Figure S16: Systematic review PRISMA diagram: depression and transactional sex 15](#_4tiiv5ec5n2x)

[Figure S17: Systematic review PRISMA diagram: anxiety and transactional sex 15](#_vpx13pm307n4)

[Figure S18: Systematic review PRISMA diagram: tobacco and transactional sex 16](#_vn22jkjvbxo7)

[Figure S19: Systematic review PRISMA diagram: opioids and transactional sex 16](#_btejrjlnj9f3)

[Figure S20: Systematic review PRISMA diagram: stimulants and transactional sex 17](#_9sah2mw0vwu9)

[Figure S21: Systematic review PRISMA diagram: alcohol and multiple sexual partners 17](#_e5v5th8tl51f)

[Figure S22: Systematic review PRISMA diagram: depression and multiple sexual partners 18](#_29gquun9nncc)

[Figure S23: Systematic review PRISMA diagram: anxiety and multiple sexual partners 18](#_kmick1br3x24)

[Figure S24: Systematic review PRISMA diagram: tobacco and multiple sexual partners 19](#_txe2y9euf5am)

[Figure S25: Systematic review PRISMA diagram: opioids and multiple sexual partners 20](#_16wfmicrw8sd)

[Figure S26: Systematic review PRISMA diagram: stimulants and multiple sexual partners 20](#_oj3imegdrnuz)

[Supplementary Box 2: Reference list of meta-analysis constituent studies 21](#_1fob9te)

[Figure S27: Meta-analysis forest plot: alcohol and medication non-adherence 62](#_3znysh7)

[Figure S28: Meta-analysis forest plot: depression and medication non-adherence 62](#_8u40gkfqwl2w)

[Figure S29: Meta-analysis forest plot: anxiety and medication non-adherence 63](#_tom3bqzij91b)

[Figure S30: Meta-analysis forest plot: pain and medication non-adherence 63](#_c7unfbxfchm6)

[Figure S31: Meta-analysis forest plot: tobacco and medication non-adherence 64](#_8hj6mtfsq344)

[Figure S32: Meta-analysis forest plot: opioids and medication non-adherence 64](#_gms2o69lll2c)

[Figure S33: Meta-analysis forest plot: stimulants and medication non-adherence 65](#_4eafcuzcd3xj)

[Figure S34: Meta-analysis forest plot: alcohol and unprotected sex 65](#_d5un6qnsark2)

[Figure S35: Meta-analysis forest plot: depression and unprotected sex 66](#_d5q4mj6kijk)

[Figure S36: Meta-analysis forest plot: anxiety and unprotected sex 66](#_u1dck246nbln)

[Figure S37: Meta-analysis forest plot: pain and unprotected sex 67](#_mo2u92zan79)

[Figure S38: Meta-analysis forest plot: tobacco and unprotected sex 67](#_6c5fzmty01fl)

[Figure S39: Meta-analysis forest plot: opioids and unprotected sex 68](#_7mgvki7ocd3c)

[Figure S40: Meta-analysis forest plot: stimulants and unprotected sex 69](#_hnjkjaewu2a6)

[Figure S41: Meta-analysis forest plot: alcohol and transactional sex 70](#_t5zsjdbnhc1p)

[Figure S42: Meta-analysis forest plot: depression and transactional sex 70](#_t9gl7kydvfsw)

[Figure S43: Meta-analysis forest plot: anxiety and transactional sex 71](#_yxdmcz8qiwc)

[Figure S44: Meta-analysis forest plot: tobacco and transactional sex 71](#_8uz8fxvtxs34)

[Figure S45: Meta-analysis forest plot: opioids and transactional sex 72](#_67e8f2ug4jt5)

[Figure S46: Meta-analysis forest plot: stimulants and transactional sex 72](#_70019m1i5o4j)

[Figure S47: Meta-analysis forest plot: alcohol and multiple sexual partners 73](#_y7omdsdx8o0a)

[Figure S48: Meta-analysis forest plot: depression and multiple sexual partners 73](#_aaizc56u3jnx)

[Figure S49: Meta-analysis forest plot: anxiety and multiple sexual partners 74](#_48bkwrd5gg9b)

[Figure S50: Meta-analysis forest plot: tobacco and multiple sexual partners 74](#_xs2b3gg374gx)

[Figure S51: Meta-analysis forest plot: opioids and multiple sexual partners 75](#_qaxacfcswpeu)

[Figure S52: Meta-analysis forest plot: stimulants and multiple sexual partners 75](#_6o81ycimrqo0)

[Table S1: Heterogeneity values from statistical output of meta-analyses eligible for the proportion of meaningfully strong effects sensitivity analysis 76](#_2et92p0)

[Figure S53: Secondary analysis: meta-analysis results (restricted to CASM-adjusted studies) 77](#_tyjcwt)

[Supplementary Box 3: Secondary analysis systematic review and meta-analysis notes 78](#_1239y7y9qk87)

[Table S2: Secondary analysis: assessing unmeasured confounding in meta-analyses restricted to CASM-adjusted estimates, by the point estimate sensitivity analysis, excluding studies assessed by proportion of meaningfully strong effects 79](#_1t3h5sf)

[Table S3: Secondary analysis: assessing unmeasured confounding in meta-analyses restricted to CASM-adjusted estimates, by proportion of meaningfully strong effects sensitivity analysis 80](#_xsmohm7uq8pp)

[Supplementary Box 4: Secondary analyses among sensitivity analyses adjusting for unmeasured confounding notes 80](#_lvldh1iaxn42)

[Table S4: Measures of association and E-values of exposure and outcome associations with comparable covariable distrust in medical institutions to gauge strength of the E-value 82](#_4d34og8)

[Supplementary Box 5: Assessing Ĝ(r,q) in the context of reference risk ratios 84](#_eih63bvgzg7w)

[Table S5: Bias factors from associations with comparable covariable distrust in medical institutions to gauge strength of T̂(r,q) 85](#_2s8eyo1)

# Supplementary Box 1: Systematic review search terms

| **Alcohol and medication non-adherence**  ("alcohol use disorder" OR "alcoholism" OR "alcohol dependence" OR "alcohol addiction" OR "alcohol abuse" OR "unhealthy alcohol use" OR "unhealthy alcohol consumption" OR "at-risk alcohol consumption" OR "at-risk alcohol use" OR "risky alcohol use" OR "risky alcohol consumption" OR "hazardous alcohol consumption" OR "hazardous alcohol use" OR "harmful alcohol use" OR "hazardous alcohol consumption") AND ("adherence" OR "compliance" OR "adhere" OR "comply") AND ("art" OR "antiretroviral therapy" OR "hiv treatment" OR "HAART" OR "cART" OR "HCV treatment" OR "hepatitis C treatment" OR "antiviral medication" OR "direct-acting antiviral" OR "DAA") AND ("PLWH" OR "HIV patient*" OR "HIV-infected" OR "human immunodeficiency virus" OR "HIV positive" or "AIDS" OR "HCV" or "hepatitis C-infected" OR "hep C" OR "hepatitis C virus" OR "hepatitis C" OR "HCV-infected")  **Tobacco and medication non-adherence**  ("smoking" OR "smoker" OR "smoked" OR "smoke" OR "tobacco" OR "tobacco product" OR "cigarette" OR "cigar") AND ("adherence" OR "compliance" OR "adhere" OR "comply") AND ("art" OR "antiretroviral therapy" OR "hiv treatment" OR "HAART" OR "cART" OR "HCV treatment" OR "hepatitis C treatment" OR "antiviral medication" OR "direct-acting antiviral" OR "DAA") AND ("PLWH" OR "HIV patient*" OR "HIV-infected" OR "human immunodeficiency virus" OR "HIV positive" or "AIDS" OR "HCV" or "hepatitis C-infected" OR "hep C" OR "hepatitis C virus" OR "hepatitis C" OR "HCV-infected")  **Opioids and medication non-adherence**  ("opioid" OR "opium" OR "opiate" OR "heroin") AND ("adherence" OR "compliance" OR "adhere" OR "comply") AND ("art" OR "antiretroviral therapy" OR "hiv treatment" OR "HAART" OR "cART" OR "HCV treatment" OR "hepatitis C treatment" OR "antiviral medication" OR "direct-acting antiviral" OR "DAA") AND ("PLWH" OR "HIV patient*" OR "HIV-infected" OR "human immunodeficiency virus" OR "HIV positive" or "AIDS" OR "HCV" or "hepatitis C-infected" OR "hep C" OR "hepatitis C virus" OR "hepatitis C" OR "HCV-infected")  **Stimulants and medication non-adherence**  ("stimulant" OR "crank" OR "ice" OR "crystal" OR "speed" OR “meth” OR “methamphetamine” OR "cocaine" OR “crack” OR “coke”) AND ("adherence" OR "compliance" OR "adhere" OR "comply") AND ("art" OR "antiretroviral therapy" OR "hiv treatment" OR "HAART" OR "cART" OR "HCV treatment" OR "hepatitis C treatment" OR "antiviral medication" OR "direct-acting antiviral" OR "DAA") AND ("PLWH" OR "HIV patient*" OR "HIV-infected" OR "human immunodeficiency virus" OR "HIV positive" or "AIDS" OR "HCV" or "hepatitis C-infected" OR "hep C" OR "hepatitis C virus" OR "hepatitis C" OR "HCV-infected")  **Depression and medication non-adherence**  ("depression" or "depressive" or "depressive symptoms" or "dysthymia") AND ("adherence" OR "compliance" OR "adhere" OR "comply") AND ("art" OR "antiretroviral therapy" OR "hiv treatment" OR "HAART" OR "cART" OR "HCV treatment" OR "hepatitis C treatment" OR "antiviral medication" OR "direct-acting antiviral" OR "DAA") AND ("PLWH" OR "HIV patient*" OR "HIV-infected" OR "human immunodeficiency virus" OR "HIV positive" or "AIDS" OR "HCV" or "hepatitis C-infected" OR "hep C" OR "hepatitis C virus" OR "hepatitis C" OR "HCV-infected")  **Anxiety and medication non-adherence**  ("anxiety" OR "anxiety symptoms" OR "anxiousness" OR "anxiety disorder") AND ("adherence" OR "compliance" OR "adhere" OR "comply") AND ("adherence" OR "compliance" OR "adhere" OR "comply") AND ("art" OR "antiretroviral therapy" OR "hiv treatment" OR "HAART" OR "cART" OR "HCV treatment" OR "hepatitis C treatment" OR "antiviral medication" OR "direct-acting antiviral" OR "DAA") AND ("PLWH" OR "HIV patient*" OR "HIV-infected" OR "human immunodeficiency virus" OR "HIV positive" or "AIDS" OR "HCV" or "hepatitis C-infected" OR "hep C" OR "hepatitis C virus" OR "hepatitis C" OR "HCV-infected")  **Pain and medication non-adherence**  ("pain" OR "pain disorder" OR "chronic pain") AND ("adherence" OR "compliance" OR "adhere" OR "comply") AND ("art" OR "antiretroviral therapy" OR "hiv treatment" OR "HAART" OR "cART" OR "HCV treatment" OR "hepatitis C treatment" OR "antiviral medication" OR "direct-acting antiviral" OR "DAA") AND ("PLWH" OR "HIV patient*" OR "HIV-infected" OR "human immunodeficiency virus" OR "HIV positive" or "AIDS" OR "HCV" or "hepatitis C-infected" OR "hep C" OR "hepatitis C virus" OR "hepatitis C" OR "HCV-infected")  **Alcohol and unprotected sex**  ("alcohol use disorder" OR "alcoholism" OR "alcohol dependence" OR "alcohol addiction" OR "alcohol abuse" OR "unhealthy alcohol use" OR "unhealthy alcohol consumption" OR "at-risk alcohol consumption" OR "at-risk alcohol use" OR "risky alcohol use" OR "risky alcohol consumption" OR "hazardous alcohol consumption" OR "hazardous alcohol use" OR "harmful alcohol use" OR "hazardous alcohol consumption") AND ("unprotected sex" OR "unprotected sexual intercourse" OR "unprotected intercourse" OR "risk* sexual behavio*" OR "sexual risk* behavio*" OR "unsafe sex" OR "unsafe intercourse" OR "unsafe sexual intercourse" OR "unsafe sexual behavio*" OR "condomless" OR "high-risk sexual behavio*" OR "high-risk sex" OR "higher-risk sexual behavio*" OR "higher-risk sex" OR "high risk sexual behavio*" OR "high risk sex" OR "higher risk sexual behavio*" OR "higher risk sex" OR "sex without contraception" OR "sex without a condom" or "sex without birth control" OR "emergency contraception" OR "non-contraceptive" OR "STI" OR "STD" OR "sexual* transmitted disease*" OR "sexual* transmitted infection*")  **Tobacco and unprotected sex**  ("smoking" OR "smoker" OR "smoked" OR "smoke" OR "tobacco" OR "tobacco product" OR "cigarette" OR "cigar") AND ("unprotected sex" OR "unprotected sexual intercourse" OR "unprotected intercourse" OR "risk* sexual behavio*" OR "sexual risk* behavio*" OR "unsafe sex" OR "unsafe intercourse" OR "unsafe sexual intercourse" OR "unsafe sexual behavio*" OR "condomless" OR "high-risk sexual behavio*" OR "high-risk sex" OR "higher-risk sexual behavio*" OR "higher-risk sex" OR "high risk sexual behavio*" OR "high risk sex" OR "higher risk sexual behavio*" OR "higher risk sex" OR "sex without contraception" OR "sex without a condom" or "sex without birth control" OR "emergency contraception" OR "non-contraceptive" OR "STI" OR "STD" OR "sexual* transmitted disease*" OR "sexual* transmitted infection*")  **Opioids and unprotected sex**  ("opioid" OR "opium" OR "opiate" OR "heroin") AND ("unprotected sex" OR "unprotected sexual intercourse" OR "unprotected intercourse" OR "risk* sexual behavio*" OR "sexual risk* behavio*" OR "unsafe sex" OR "unsafe intercourse" OR "unsafe sexual intercourse" OR "unsafe sexual behavio*" OR "condomless" OR "high-risk sexual behavio*" OR "high-risk sex" OR "higher-risk sexual behavio*" OR "higher-risk sex" OR "high risk sexual behavio*" OR "high risk sex" OR "higher risk sexual behavio*" OR "higher risk sex" OR "sex without contraception" OR "sex without a condom" or "sex without birth control" OR "emergency contraception" OR "non-contraceptive" OR "STI" OR "STD" OR "sexual* transmitted disease*" OR "sexual* transmitted infection*")  **Stimulants and unprotected sex**  ("stimulant" OR "crank" OR "ice" OR "crystal" OR "speed" OR “meth” OR “methamphetamine” OR "cocaine" OR “crack” OR “coke”) AND ("unprotected sex" OR "unprotected sexual intercourse" OR "unprotected intercourse" OR "risk* sexual behavio*" OR "sexual risk* behavio*" OR "unsafe sex" OR "unsafe intercourse" OR "unsafe sexual intercourse" OR "unsafe sexual behavio*" OR "condomless" OR "high-risk sexual behavio*" OR "high-risk sex" OR "higher-risk sexual behavio*" OR "higher-risk sex" OR "high risk sexual behavio*" OR "high risk sex" OR "higher risk sexual behavio*" OR "higher risk sex" OR "sex without contraception" OR "sex without a condom" or "sex without birth control" OR "emergency contraception" OR "non-contraceptive" OR "STI" OR "STD" OR "sexual* transmitted disease*" OR "sexual* transmitted infection*")  **Depression and unprotected sex**  ("depression" or "depressive" or "depressive symptoms" or "dysthymia") AND ("unprotected sex" OR "unprotected sexual intercourse" OR "unprotected intercourse" OR "risk* sexual behavio*" OR "sexual risk* behavio*" OR "unsafe sex" OR "unsafe intercourse" OR "unsafe sexual intercourse" OR "unsafe sexual behavio*" OR "condomless" OR "high-risk sexual behavio*" OR "high-risk sex" OR "higher-risk sexual behavio*" OR "higher-risk sex" OR "high risk sexual behavio*" OR "high risk sex" OR "higher risk sexual behavio*" OR "higher risk sex" OR "sex without contraception" OR "sex without a condom" or "sex without birth control" OR "emergency contraception" OR "non-contraceptive" OR "STI" OR "STD" OR "sexual* transmitted disease*" OR "sexual* transmitted infection*")  **Anxiety and unprotected sex**  ("anxiety" OR "anxiety symptoms" OR "anxiousness" OR "anxiety disorder") AND ("unprotected sex" OR "unprotected sexual intercourse" OR "unprotected intercourse" OR "risk* sexual behavio*" OR "sexual risk* behavio*" OR "unsafe sex" OR "unsafe intercourse" OR "unsafe sexual intercourse" OR "unsafe sexual behavio*" OR "condomless" OR "high-risk sexual behavio*" OR "high-risk sex" OR "higher-risk sexual behavio*" OR "higher-risk sex" OR "high risk sexual behavio*" OR "high risk sex" OR "higher risk sexual behavio*" OR "higher risk sex" OR "sex without contraception" OR "sex without a condom" or "sex without birth control" OR "emergency contraception" OR "non-contraceptive" OR "STI" OR "STD" OR "sexual* transmitted disease*" OR "sexual* transmitted infection*")  **Pain and unprotected sex**  ("pain" OR "pain disorder" OR "chronic pain") AND ("unprotected sex" OR "unprotected sexual intercourse" OR "unprotected intercourse" OR "risk* sexual behavio*" OR "sexual risk* behavio*" OR "unsafe sex" OR "unsafe intercourse" OR "unsafe sexual intercourse" OR "unsafe sexual behavio*" OR "condomless" OR "high-risk sexual behavio*" OR "high-risk sex" OR "higher-risk sexual behavio*" OR "higher-risk sex" OR "high risk sexual behavio*" OR "high risk sex" OR "higher risk sexual behavio*" OR "higher risk sex" OR "sex without contraception" OR "sex without a condom" or "sex without birth control" OR "emergency contraception" OR "non-contraceptive" OR "STI" OR "STD" OR "sexual* transmitted disease*" OR "sexual* transmitted infection*")  **Alcohol and transactional sex and alcohol and multiple sexual partners**  ("alcohol use disorder" OR "alcoholism" OR "alcohol dependence" OR "alcohol addiction" OR "alcohol abuse" OR "unhealthy alcohol use" OR "unhealthy alcohol consumption" OR "at-risk alcohol consumption" OR "at-risk alcohol use" OR "risky alcohol use" OR "risky alcohol consumption" OR "hazardous alcohol consumption" OR "hazardous alcohol use" OR "harmful alcohol use" OR "hazardous alcohol consumption") AND ("multiple sexual partners" OR "multiple sex partners" OR "numerous sexual partners" OR "number of sexual partners" OR "concurrent sexual partners" OR "concurrent sex partners" OR "commercial sex" OR "transactional sex" OR "female sex worker*" OR "male sex worker*" OR "sex trade" OR "sex work*" OR "prostitution" OR "exchange sex")  **Tobacco and transactional sex and tobacco and multiple sexual partners**  ("smoking" OR "smoker" OR "smoked" OR "smoke" OR "tobacco" OR "tobacco product" OR "cigarette" OR "cigar") AND ("multiple sexual partners" OR "multiple sex partners" OR "numerous sexual partners" OR "number of sexual partners" OR "concurrent sexual partners" OR "concurrent sex partners" OR "commercial sex" OR "transactional sex" OR "female sex worker*" OR "male sex worker*" OR "sex trade" OR "sex work*" OR "prostitution" OR "exchange sex")  **Opioids and transactional sex and opioids and multiple sexual partners**  ("opioid" OR "opium" OR "opiate" OR "heroin") AND ("multiple sexual partners" OR "multiple sex partners" OR "numerous sexual partners" OR "number of sexual partners" OR "concurrent sexual partners" OR "concurrent sex partners" OR "commercial sex" OR "transactional sex" OR "female sex worker*" OR "male sex worker*" OR "sex trade" OR "sex work*" OR "prostitution" OR "exchange sex")  **Stimulants and transactional sex and stimulants and multiple sexual partners**  ("stimulant" OR "crank" OR "ice" OR "crystal" OR "speed" OR “meth” OR “methamphetamine” OR "cocaine" OR “crack” OR “coke”) AND ("multiple sexual partners" OR "multiple sex partners" OR "numerous sexual partners" OR "number of sexual partners" OR "concurrent sexual partners" OR "concurrent sex partners" OR "commercial sex" OR "transactional sex" OR "female sex worker*" OR "male sex worker*" OR "sex trade" OR "sex work*" OR "prostitution" OR "exchange sex")  **Depression and transactional sex and depression and multiple sexual partners**  ("depression" or "depressive" or "depressive symptoms" or "dysthymia") AND ("multiple sexual partners" OR "multiple sex partners" OR "numerous sexual partners" OR "number of sexual partners" OR "concurrent sexual partners" OR "concurrent sex partners" OR "commercial sex" OR "transactional sex" OR "female sex worker*" OR "male sex worker*" OR "sex trade" OR "sex work*" OR "prostitution" OR "exchange sex")  **Anxiety and transactional sex and anxiety and multiple sexual partners**  ("anxiety" OR "anxiety symptoms" OR "anxiousness" OR "anxiety disorder") AND ("multiple sexual partners" OR "multiple sex partners" OR "numerous sexual partners" OR "number of sexual partners" OR "concurrent sexual partners" OR "concurrent sex partners" OR "commercial sex" OR "transactional sex" OR "female sex worker*" OR "male sex worker*" OR "sex trade" OR "sex work*" OR "prostitution" OR "exchange sex")  **Pain and transactional sex and pain and multiple sexual partners**  ("pain" OR "pain disorder" OR "chronic pain") AND ("multiple sexual partners" OR "multiple sex partners" OR "numerous sexual partners" OR "number of sexual partners" OR "concurrent sexual partners" OR "concurrent sex partners" OR "commercial sex" OR "transactional sex" OR "female sex worker*" OR "male sex worker*" OR "sex trade" OR "sex work*" OR "prostitution" OR "exchange sex") |
| --- |

# Figure S1: Systematic review PRISMA diagram: alcohol and medication non-adherence


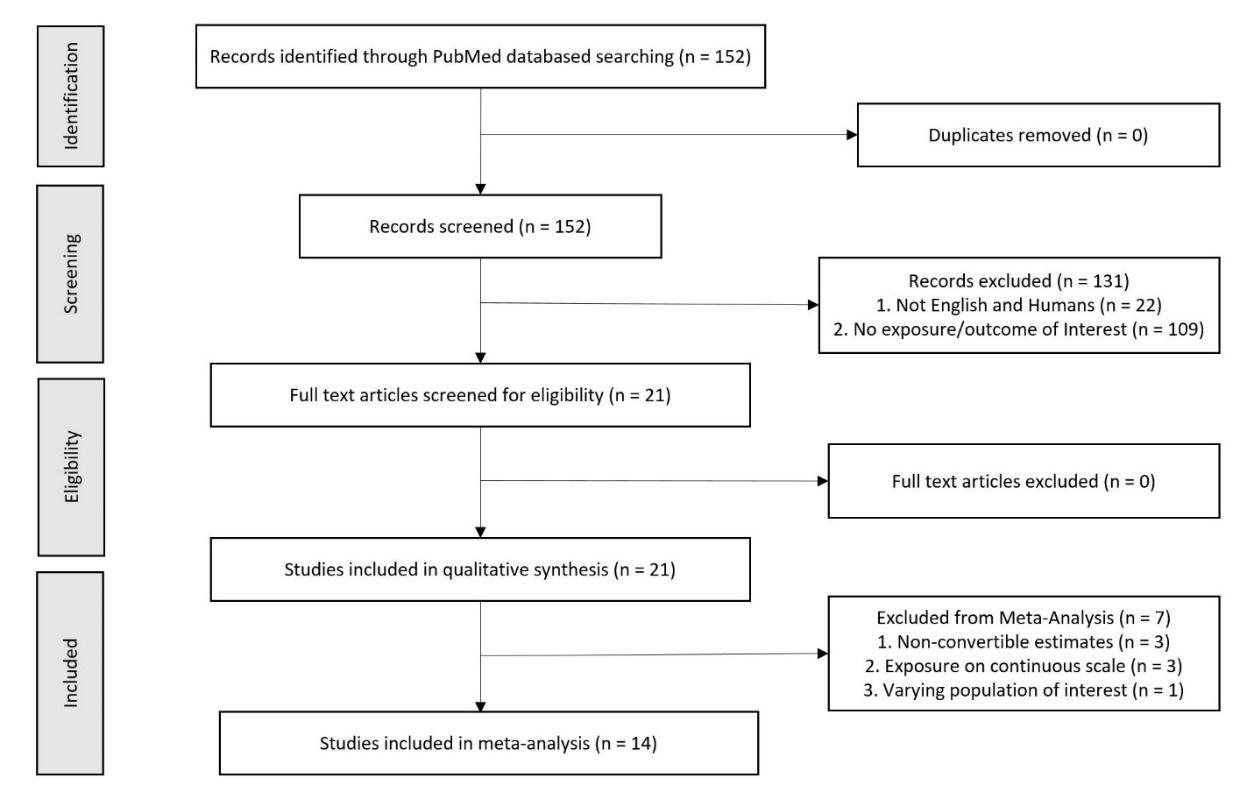


# Figure S2: Systematic review PRISMA diagram: depression and medication non-adherence


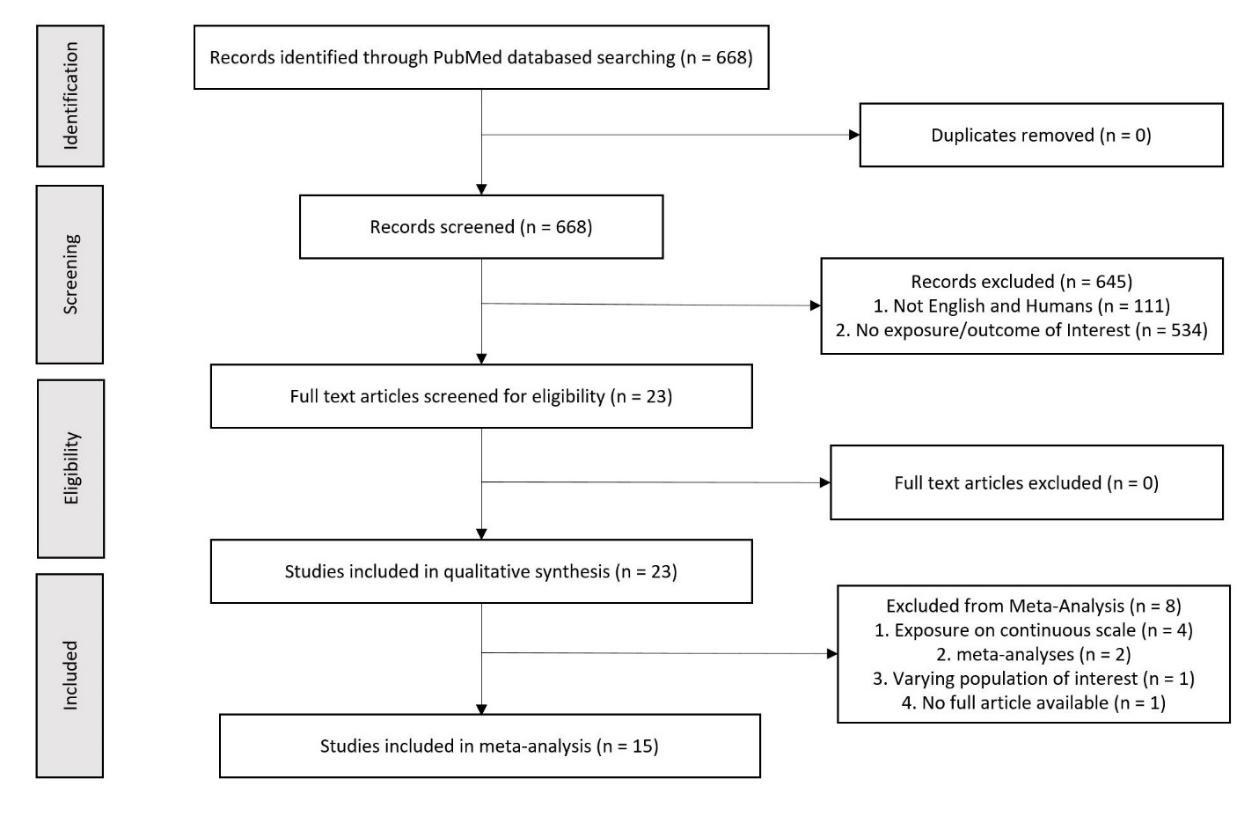


# Figure S3: Systematic review PRISMA diagram: anxiety and medication non-adherence


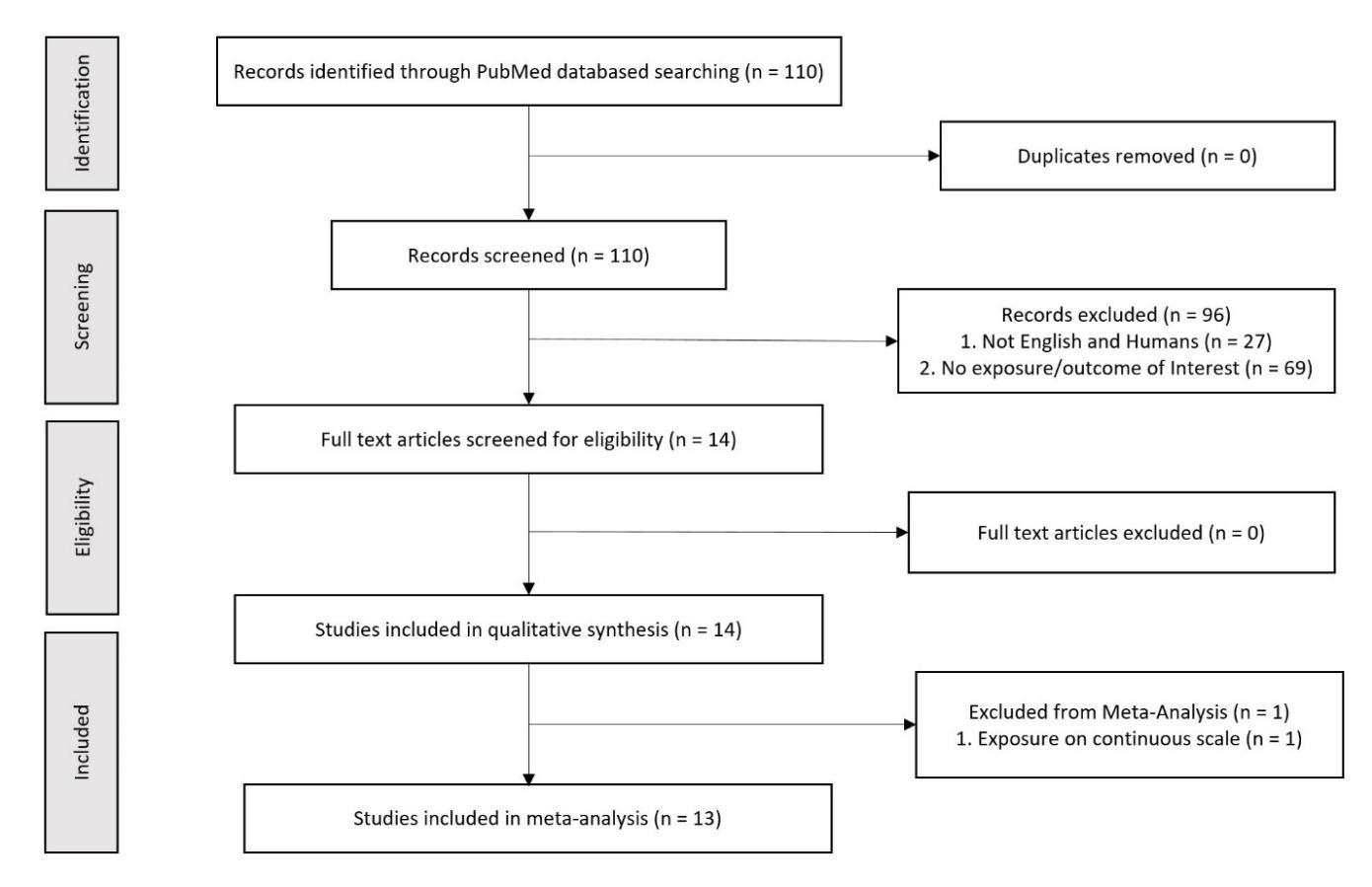


# Figure S4: Systematic review PRISMA diagram: pain and medication non-adherence


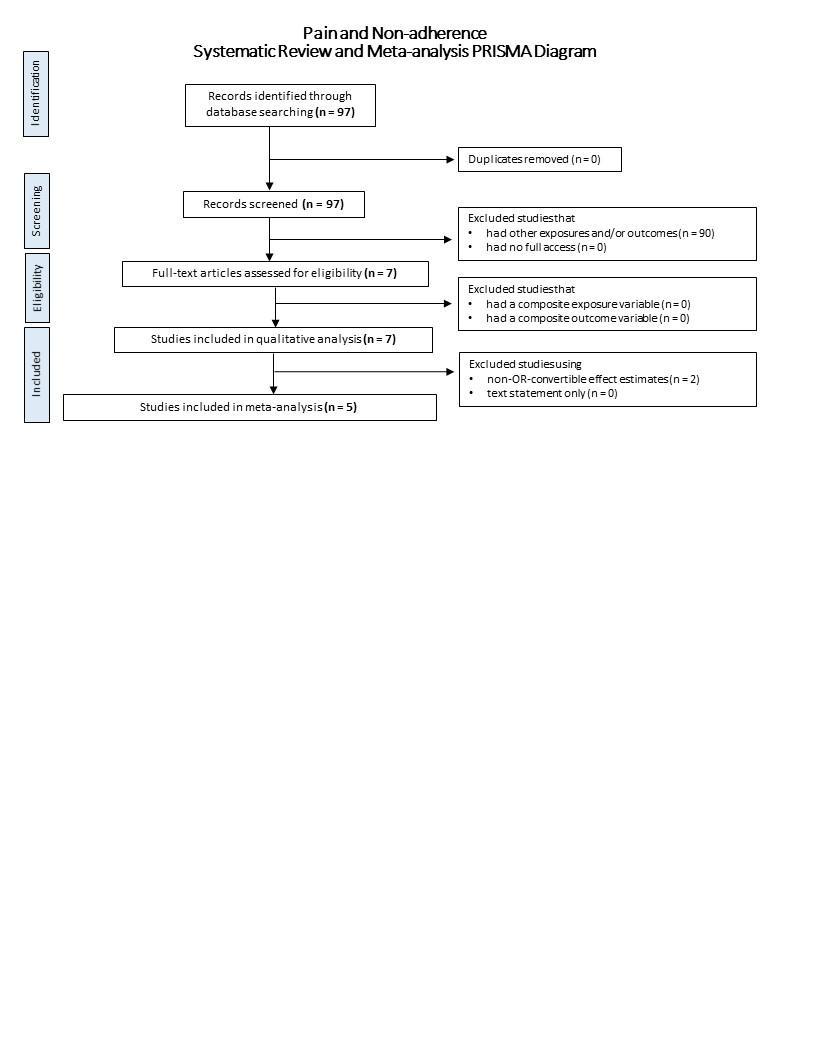


# Figure S5: Systematic review PRISMA diagram: tobacco and medication non-adherence


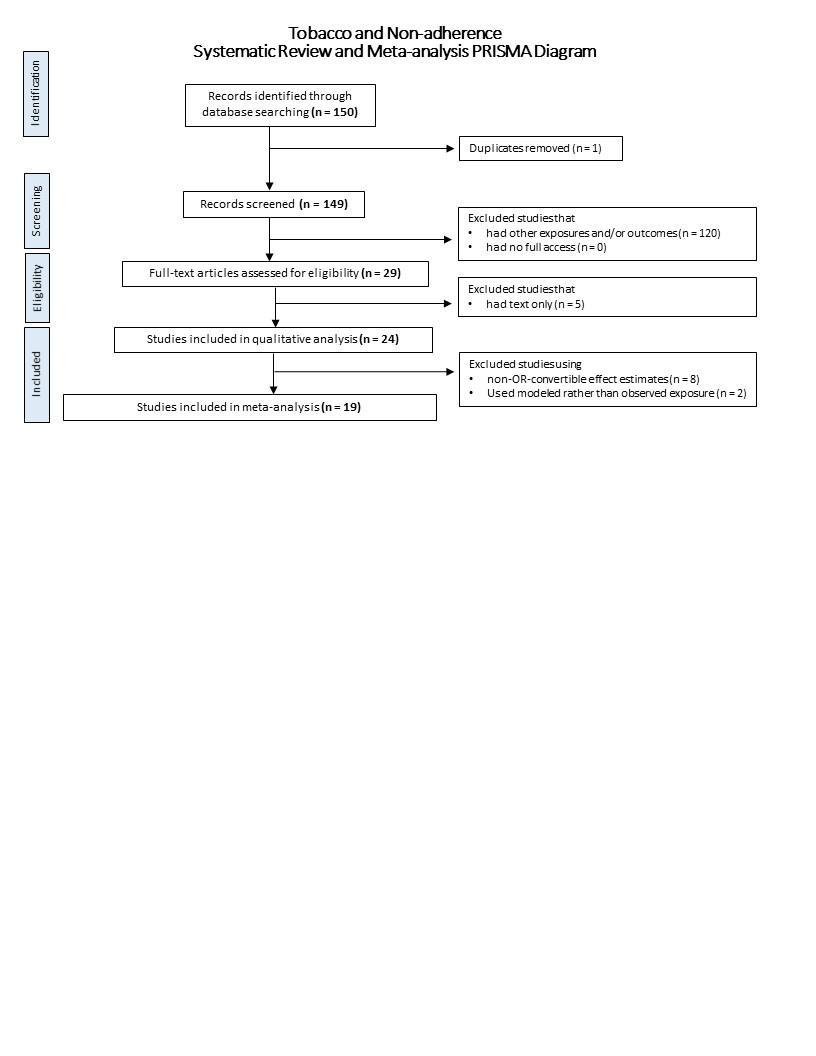


# Figure S6: Systematic review PRISMA diagram: opioids and medication non-adherence


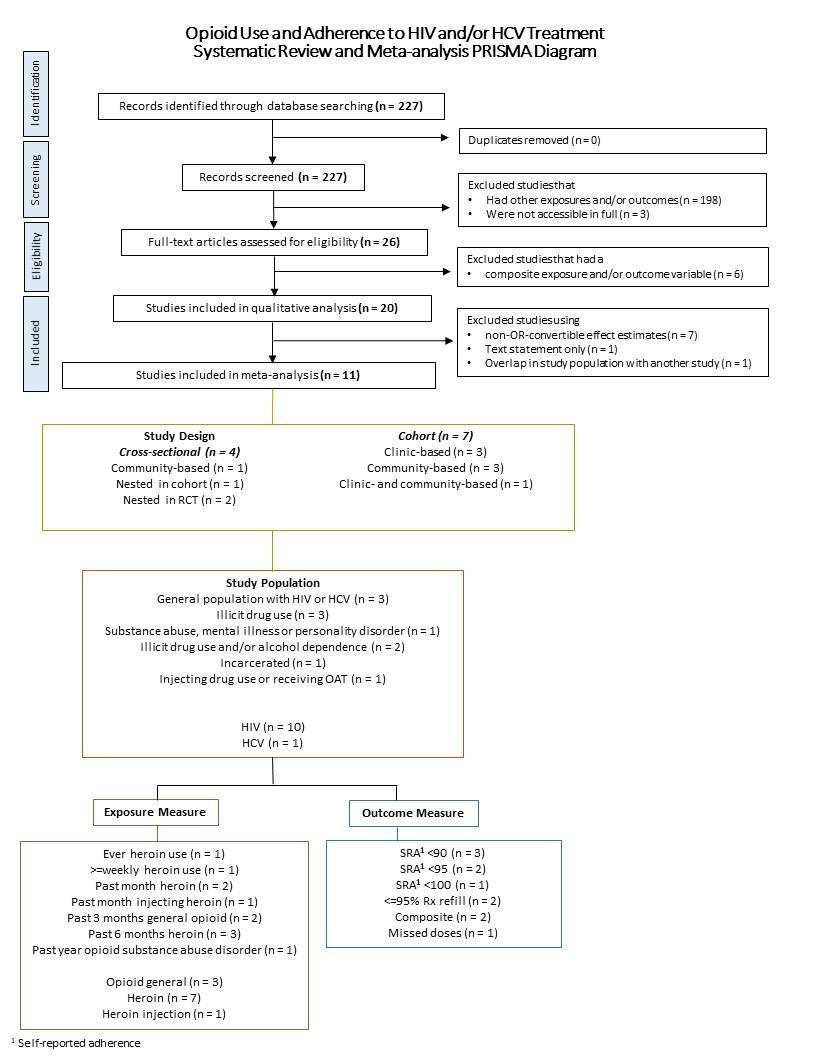


# Figure S7: Systematic review PRISMA diagram: stimulants and medication non-adherence


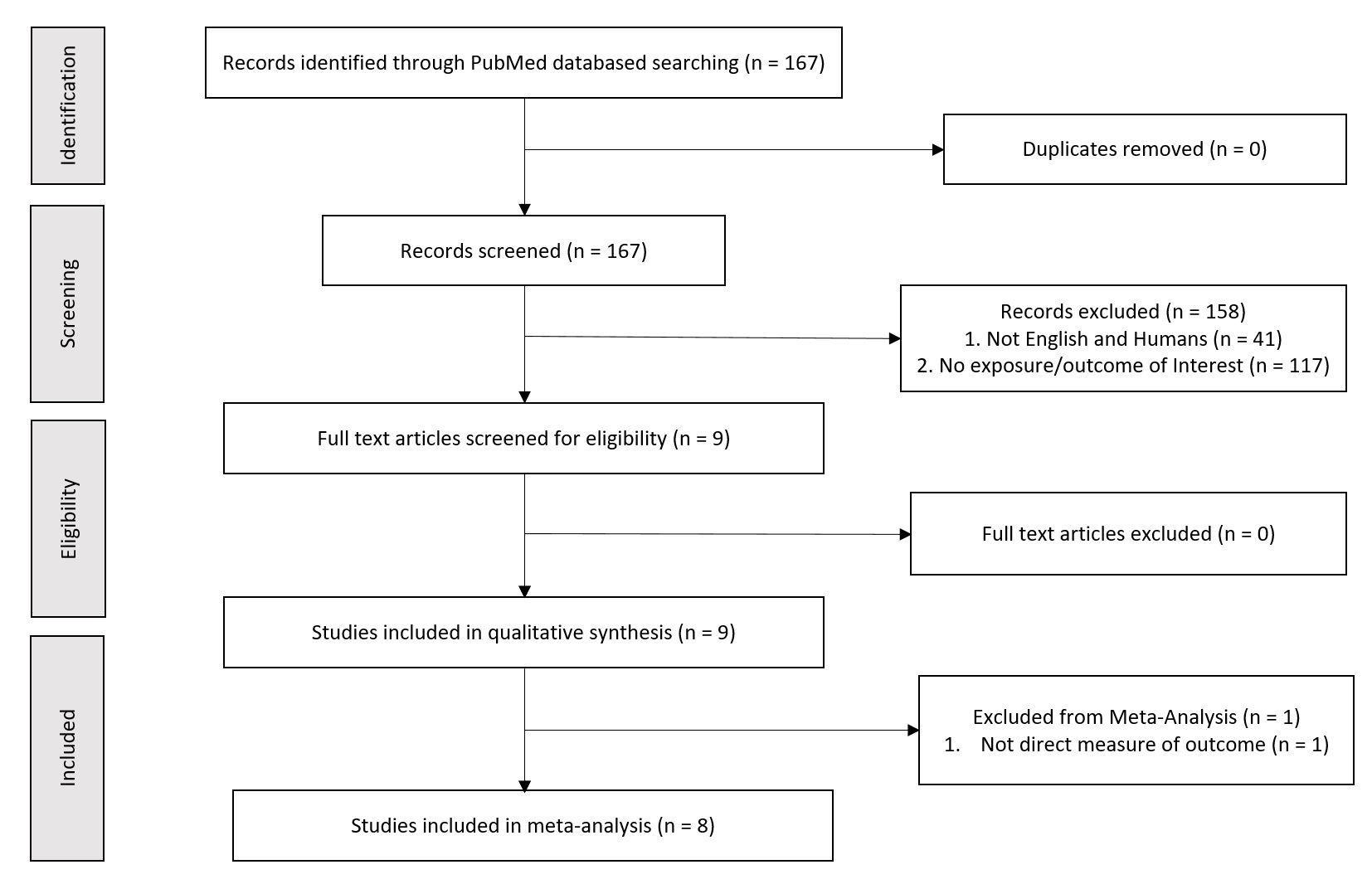


# Figure S8: Systematic review PRISMA diagram: alcohol and unprotected sex


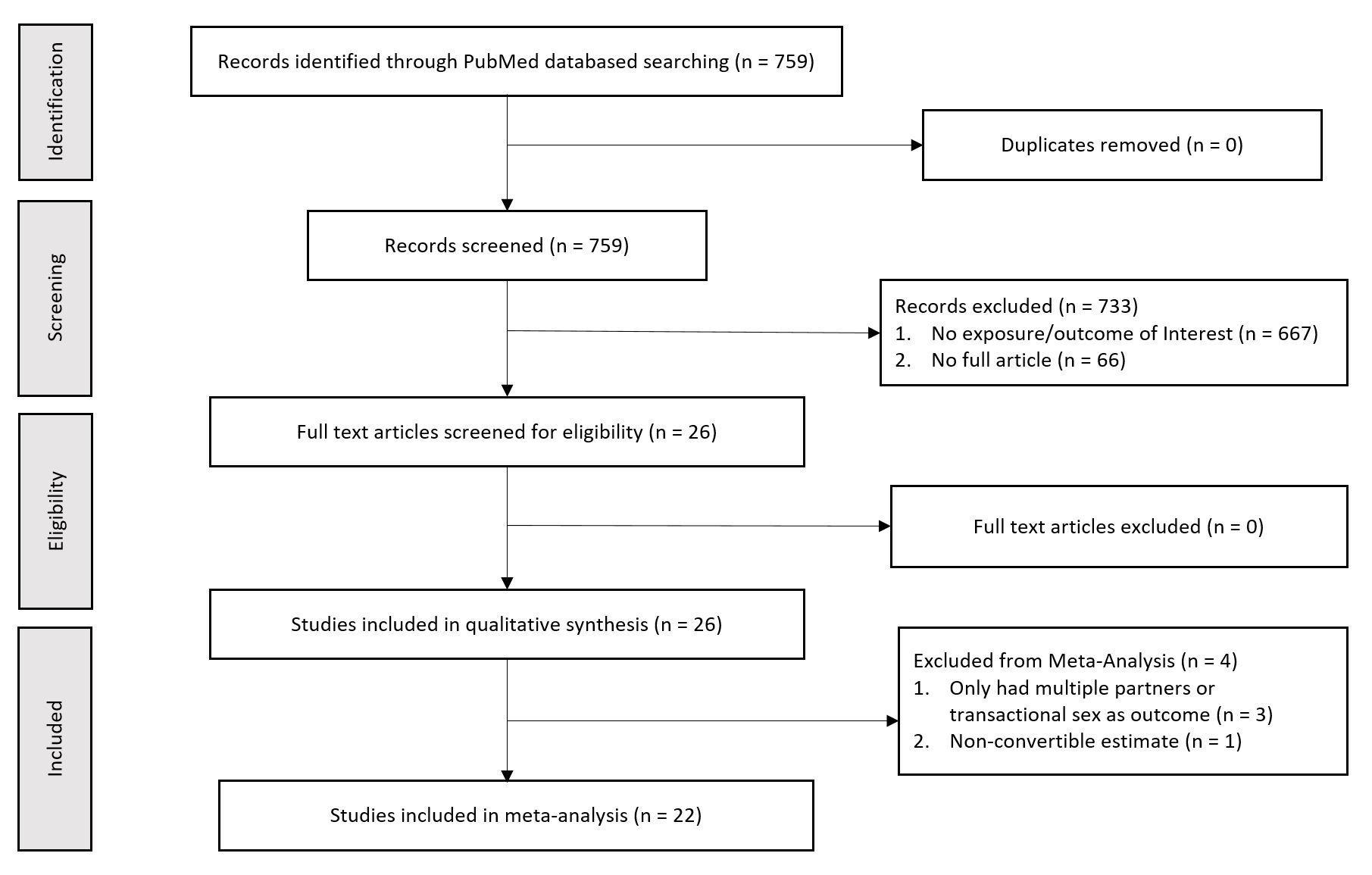


# Figure S9: Systematic review PRISMA diagram: depression and unprotected sex


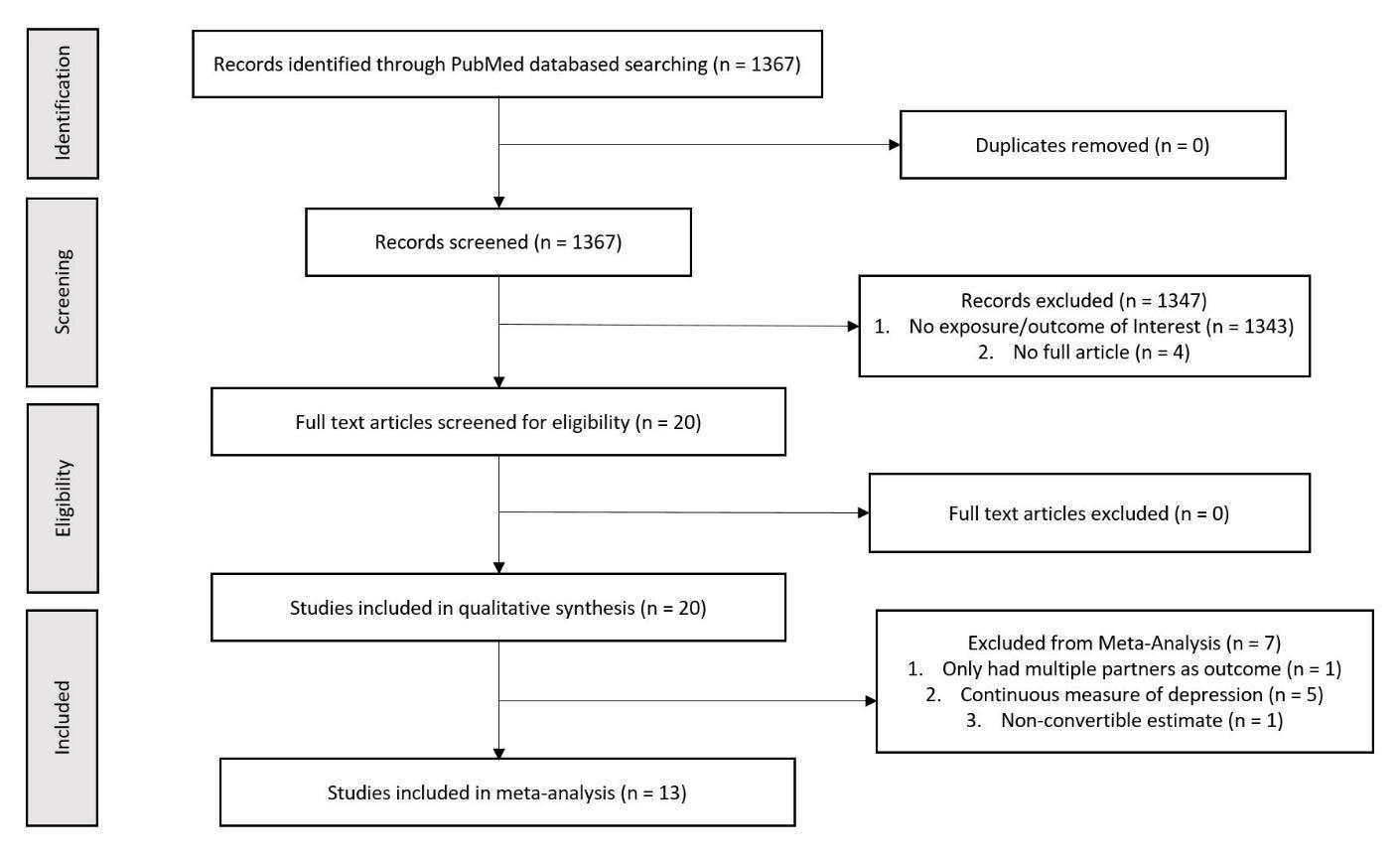


# Figure S10: Systematic review PRISMA diagram: anxiety and unprotected sex


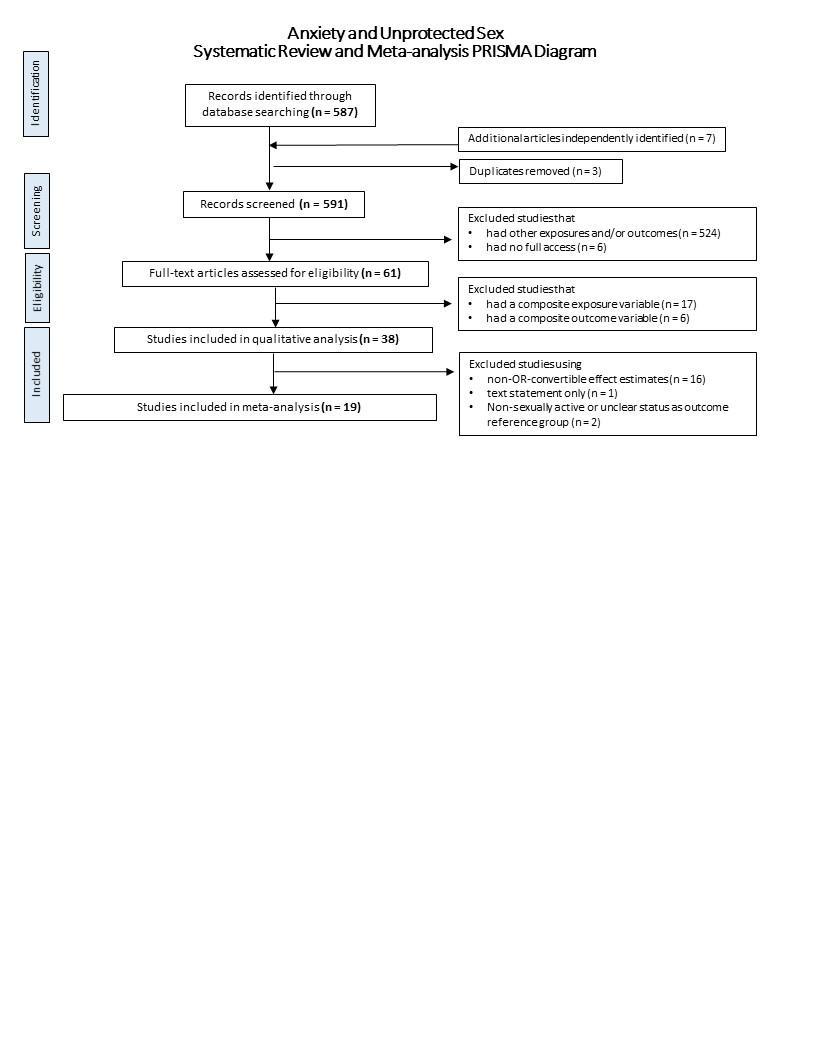


# Figure S11: Systematic review PRISMA diagram: pain and unprotected sex


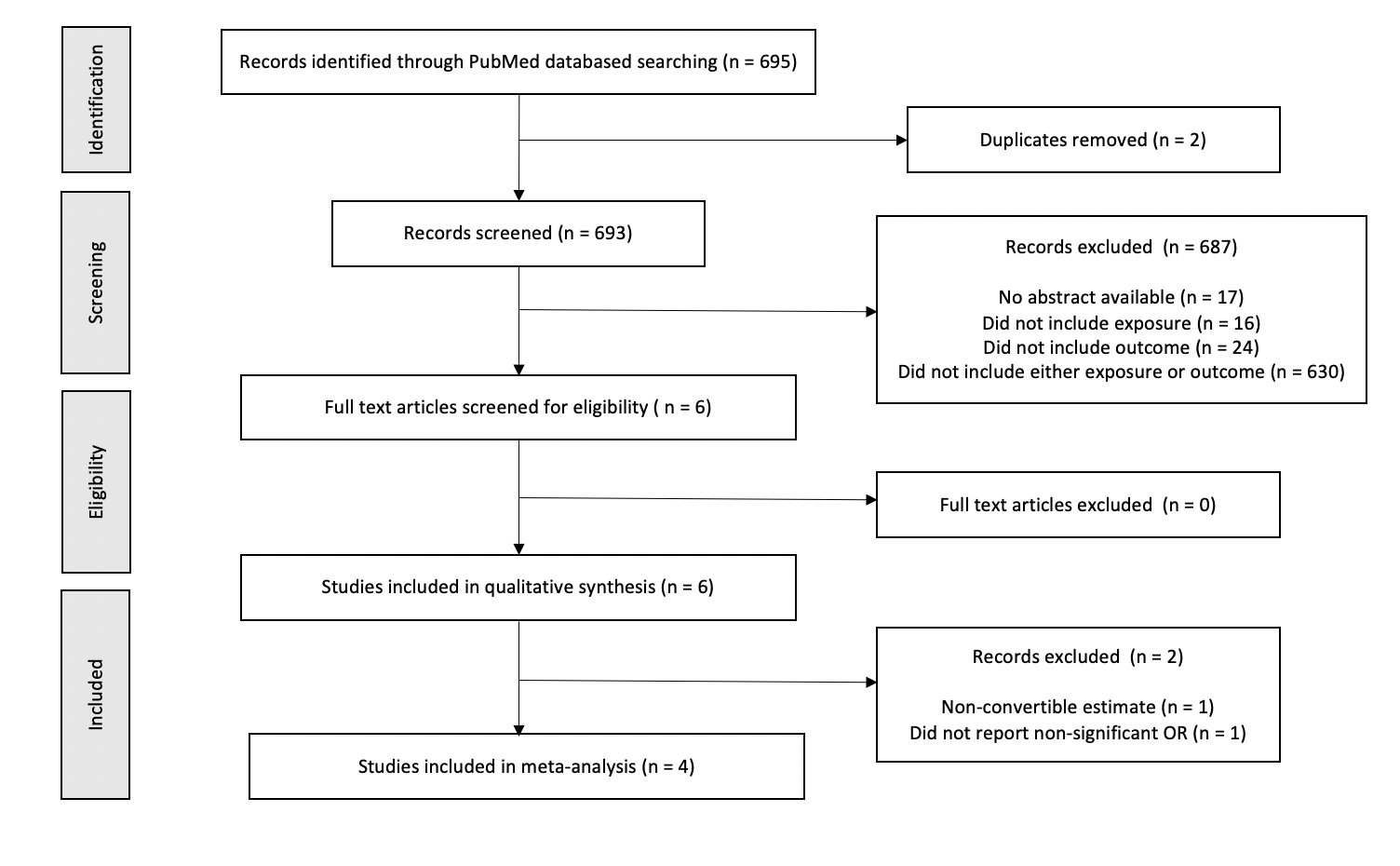


# Figure S12: Systematic review PRISMA diagram: tobacco and unprotected sex


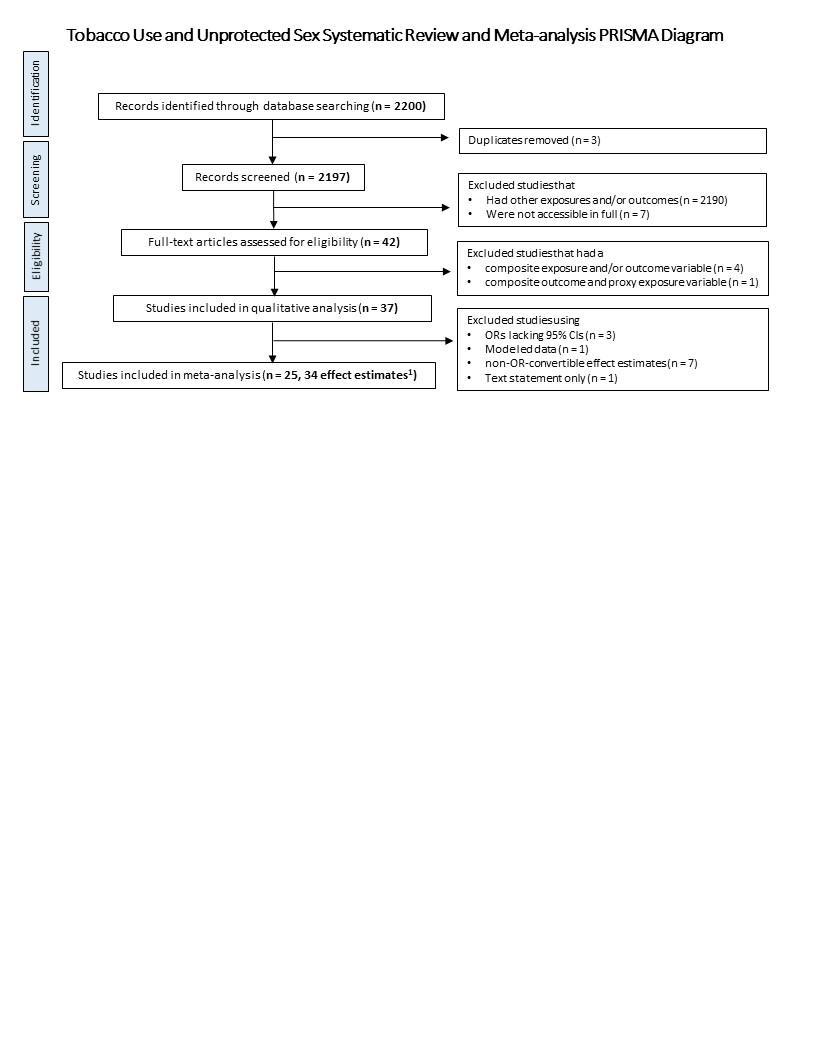


# Figure S13: Systematic review PRISMA diagram: opioids and unprotected sex


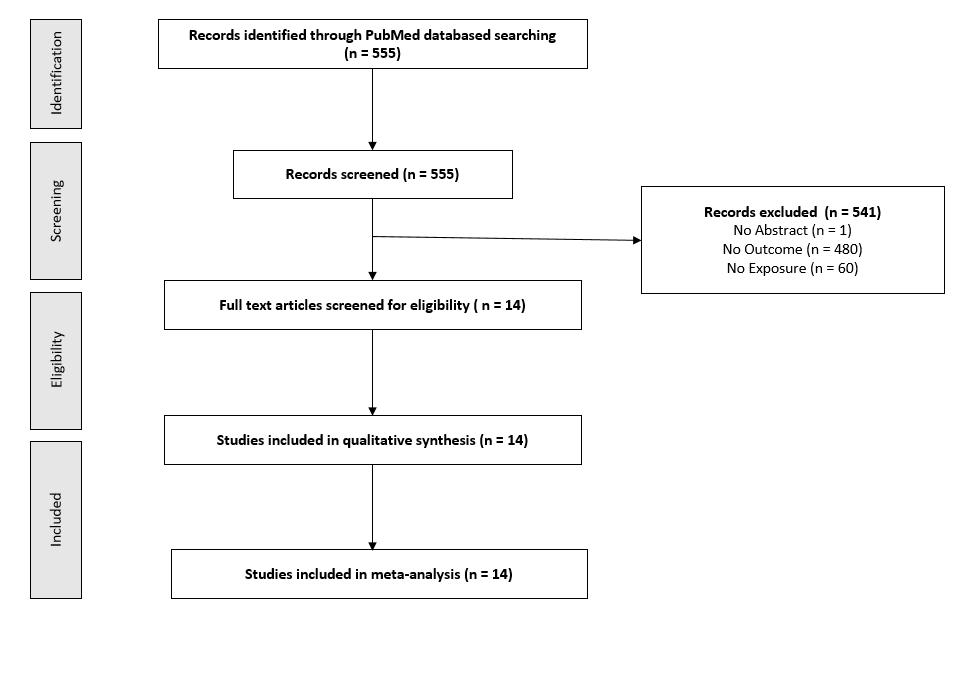


# Figure S14: Systematic review PRISMA diagram: stimulants and unprotected sex


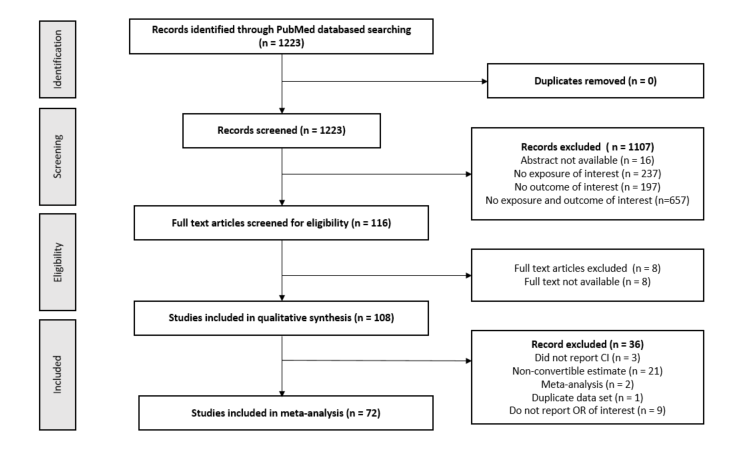


# Figure S15: Systematic review PRISMA diagram: alcohol and transactional sex


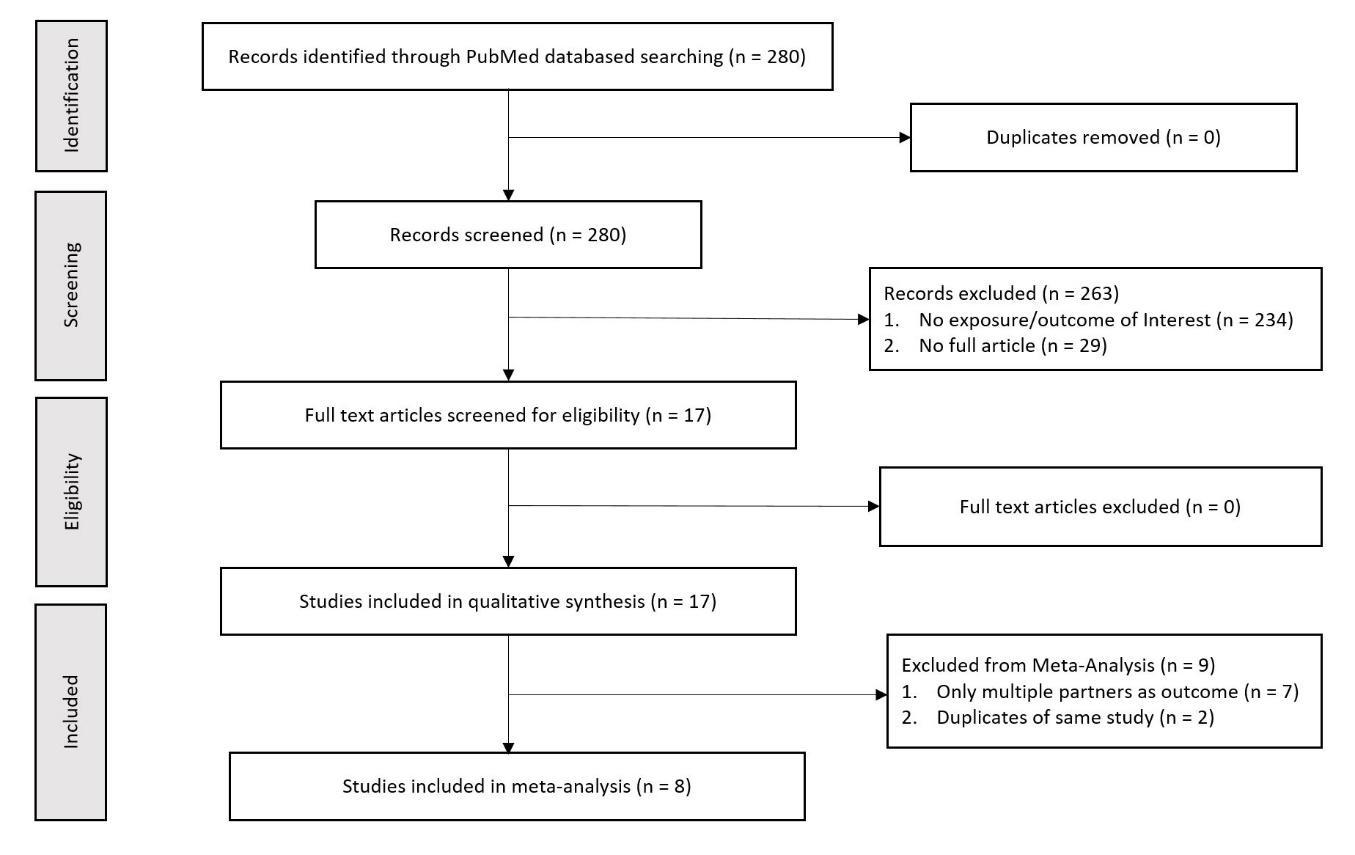


# Figure S16: Systematic review PRISMA diagram: depression and transactional sex


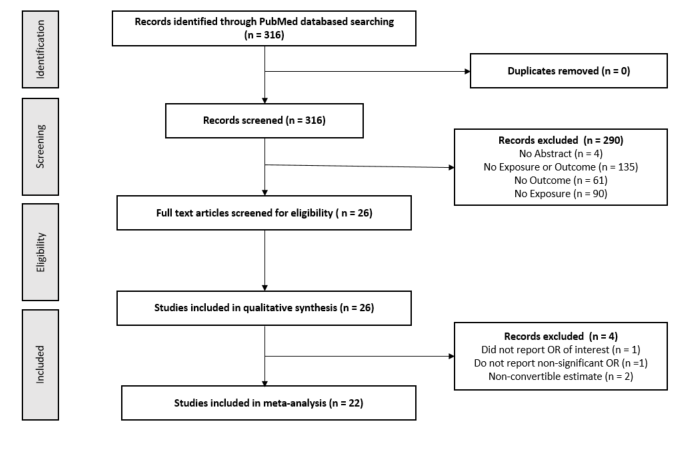


# Figure S17: Systematic review PRISMA diagram: anxiety and transactional sex


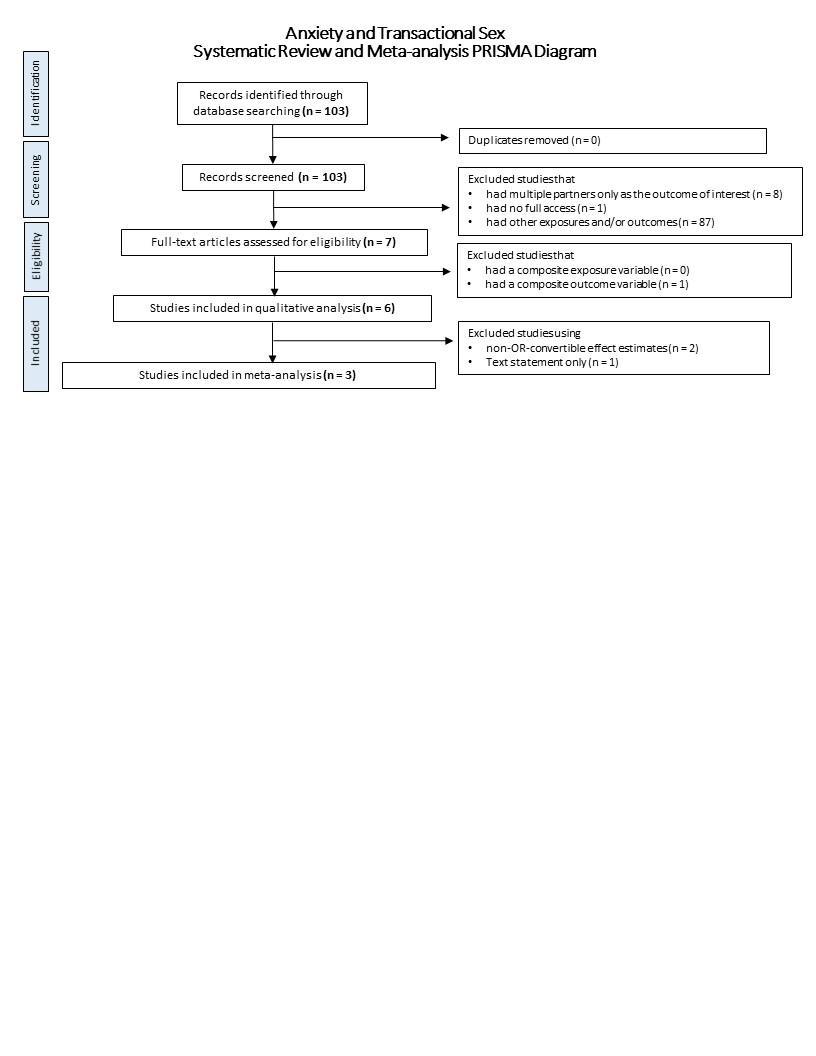


**NOTE:** Pain and transactional sex yielded no eligible studies.

# Figure S18: Systematic review PRISMA diagram: tobacco and transactional sex


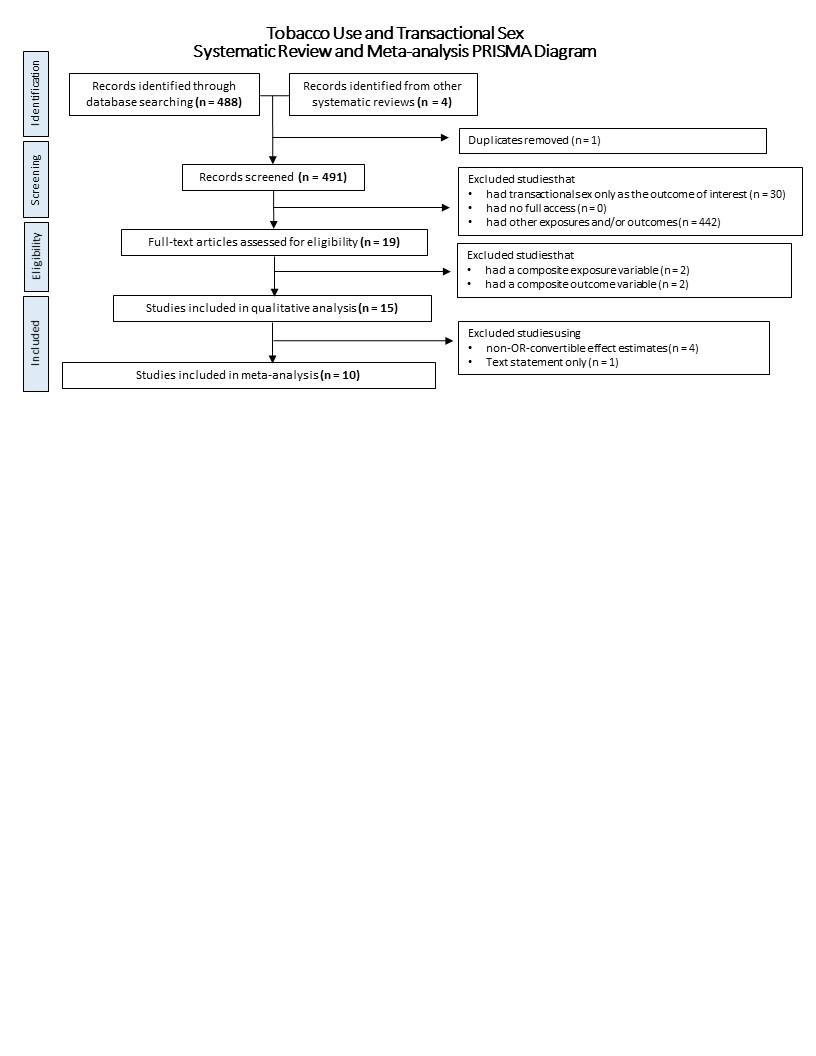


# Figure S19: Systematic review PRISMA diagram: opioids and transactional sex


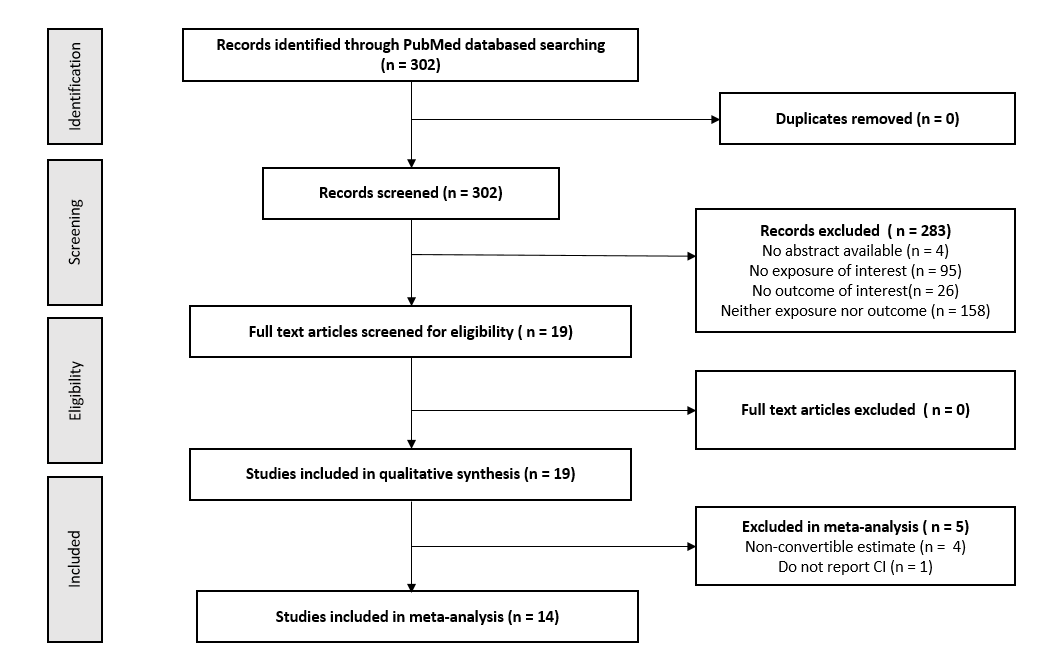


# Figure S20: Systematic review PRISMA diagram: stimulants and transactional sex


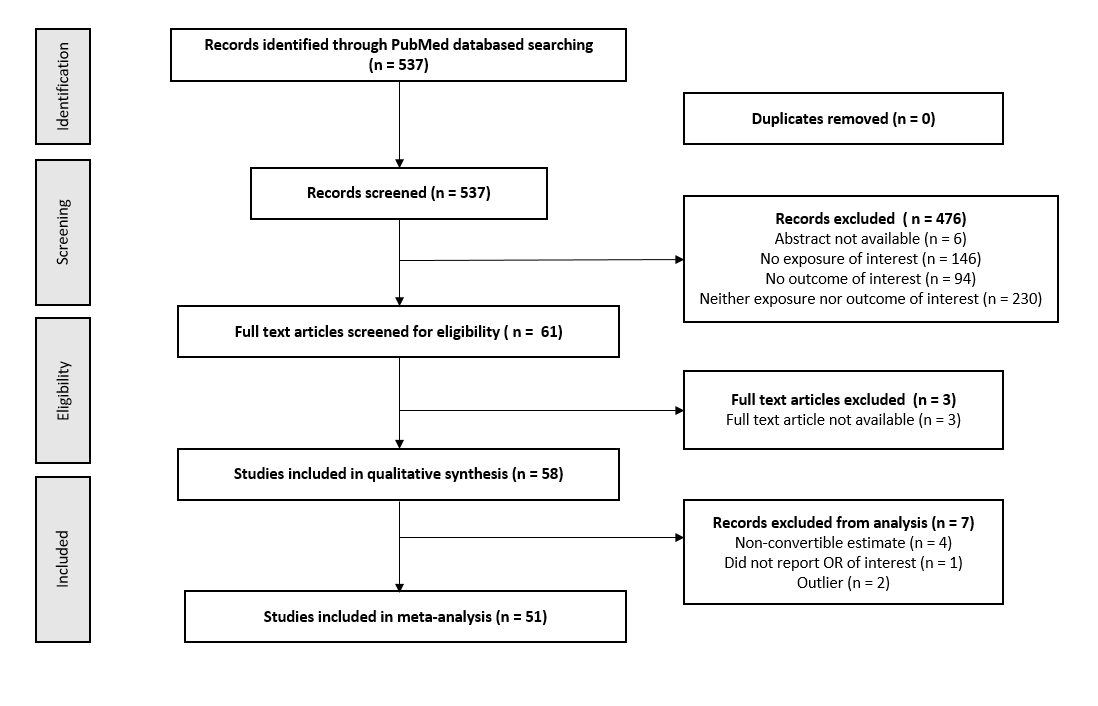


# Figure S21: Systematic review PRISMA diagram: alcohol and multiple sexual partners


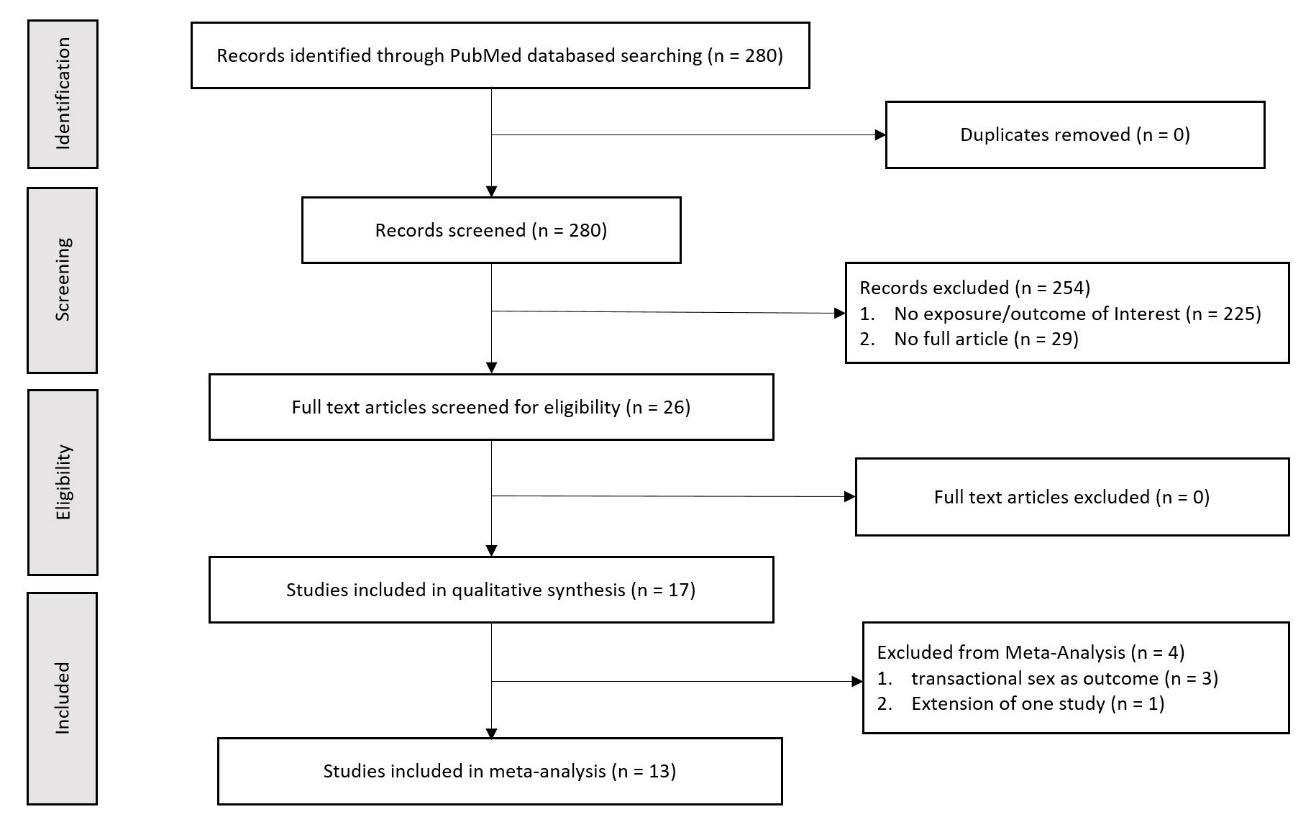


# Figure S22: Systematic review PRISMA diagram: depression and multiple sexual partners


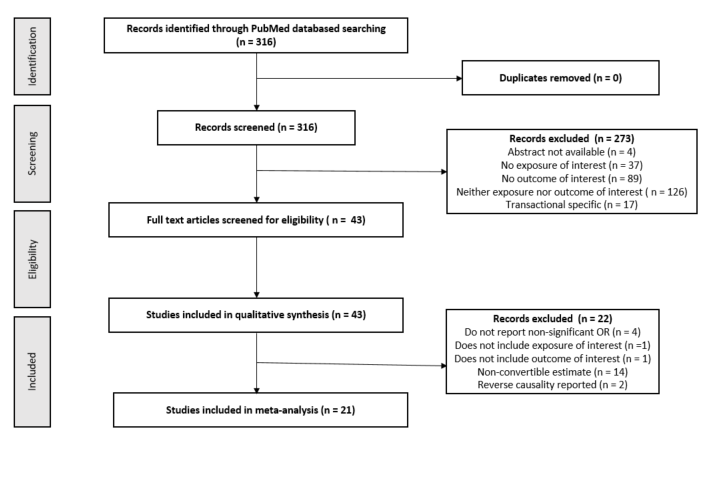


# Figure S23: Systematic review PRISMA diagram: anxiety and multiple sexual partners


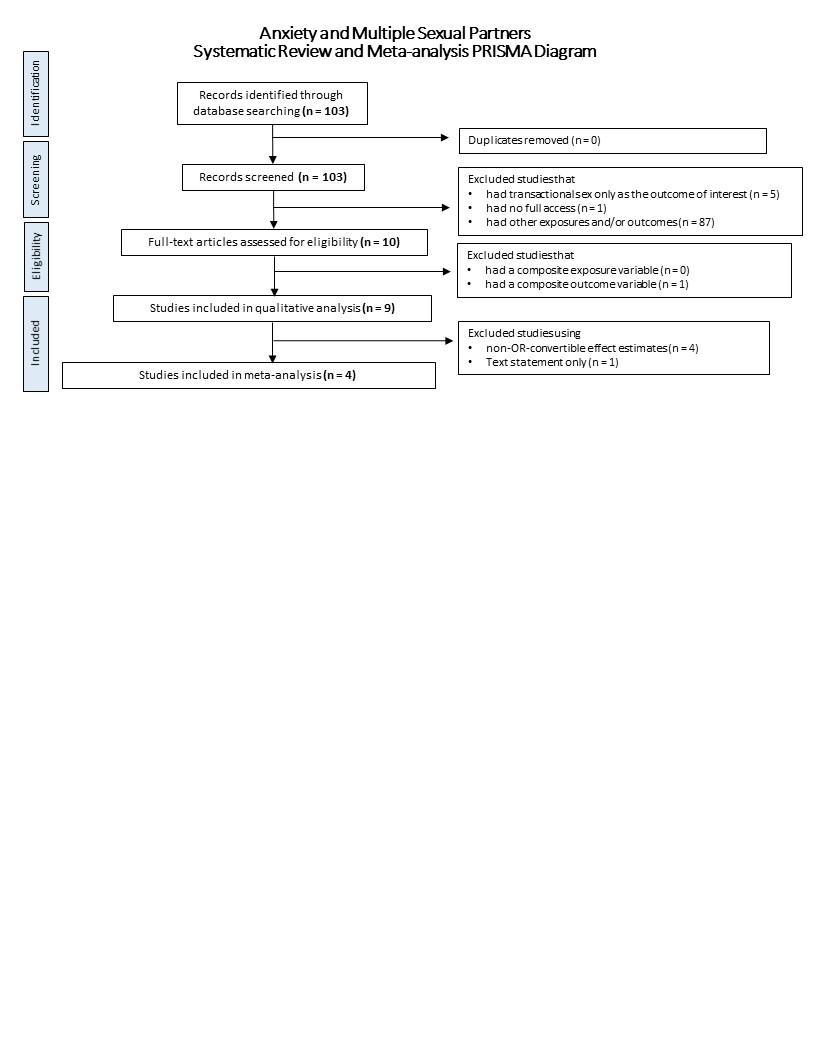


**NOTE:** Pain and multiple sexual partners yielded a single study.

# Figure S24: Systematic review PRISMA diagram: tobacco and multiple sexual partners


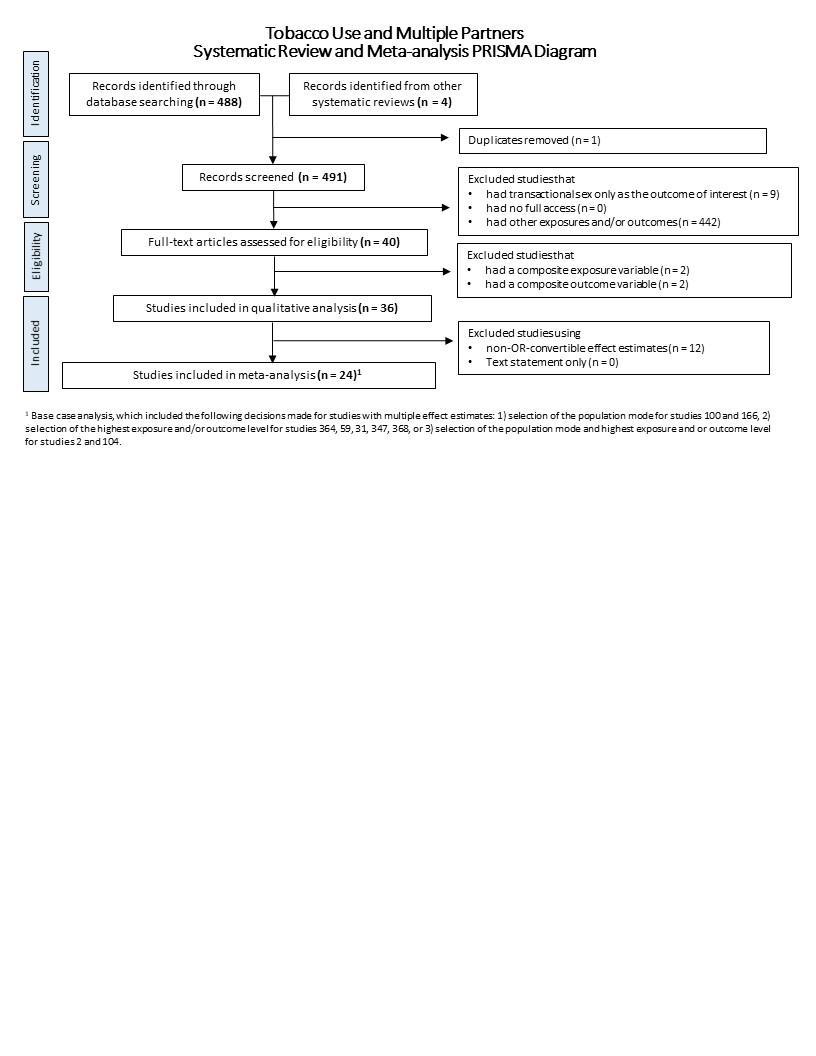


# Figure S25: Systematic review PRISMA diagram: opioids and multiple sexual partners


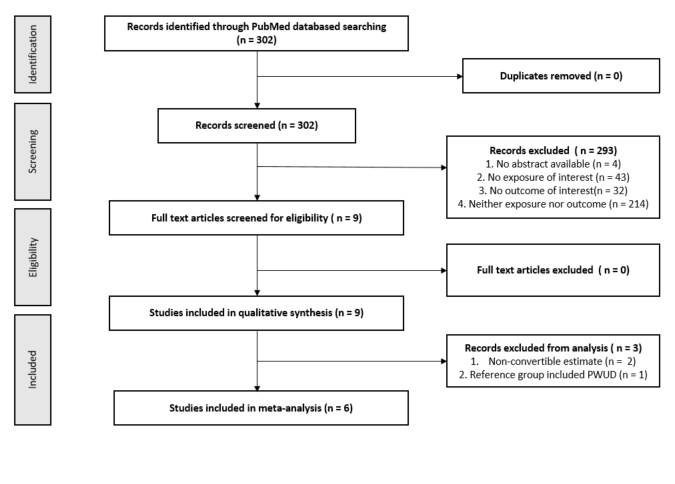


# Figure S26: Systematic review PRISMA diagram: stimulants and multiple sexual partners


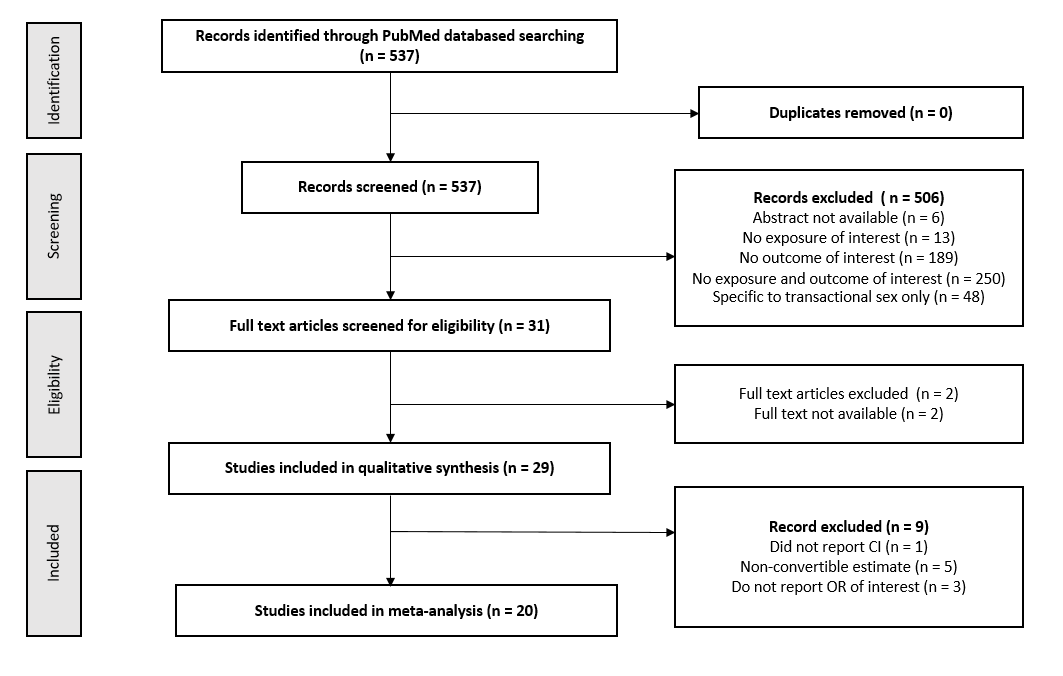


# Supplementary Box 2: Reference list of meta-analysis constituent studies

| **References for alcohol and medication non-adherence**  Denison JA, Koole O, Tsui S, et al. Incomplete adherence among treatment-experienced adults on antiretroviral therapy in Tanzania, Uganda and Zambia. *AIDS*. 2015;29(3):361-371. doi:10.1097/QAD.0000000000000543  Dever, J.B., Ducom, J.H., Ma, A. *et al.* Engagement in Care of High-Risk Hepatitis C Patients with Interferon-Free Direct-Acting Antiviral Therapies. *Dig Dis Sci* 62, 1472–1479 (2017). https://doi.org/10.1007/s10620-017-4548-4  Ferro EG, Weikum D, Vagenas P, et al. Alcohol use disorders negatively influence antiretroviral medication adherence among men who have sex with men in Peru. *AIDS Care*. 2015;27(1):93-104. doi:10.1080/09540121.2014.963013  Kim, Ji & Yang, Youngran & Kim, Hyun. (2018). The Impact of Alcohol Use on Antiretroviral-Therapy Adherence in Koreans Living with HIV. Asian Nursing Research. 12. 10.1016/j.anr.2018.10.002.  King RM, Vidrine DJ, Danysh HE, Fletcher FE, McCurdy S, Arduino RC, Gritz ER. Factors associated with nonadherence to antiretroviral therapy in HIV-positive smokers. AIDS Patient Care STDS. 2012 Aug;26(8):479-85. doi: 10.1089/apc.2012.0070. Epub 2012 May 21. PMID: 22612468; PMCID: PMC3407390.  Muessig KE, McLaughlin MM, Nie JM, Cai W, Zheng H, Yang L, Tucker JD. Suboptimal antiretroviral therapy adherence among HIV-infected adults in Guangzhou, China. AIDS Care. 2014;26(8):988-95. doi: 10.1080/09540121.2014.897912. Epub 2014 Mar 26. PMID: 24666239; PMCID: PMC4024070.  Paolillo EW, Gongvatana A, Umlauf A, Letendre SL, Moore DJ. At-Risk Alcohol Use is Associated with Antiretroviral Treatment Nonadherence Among Adults Living with HIV/AIDS. Alcohol Clin Exp Res. 2017 Aug;41(8):1518-1525. doi: 10.1111/acer.13433. Epub 2017 Jul 5. PMID: 28679147; PMCID: PMC5564671.  Peretti-Watel P, Spire B, Lert F, Obadia Y; VESPA Group. Drug use patterns and adherence to treatment among HIV-positive patients: evidence from a large sample of French outpatients (ANRS-EN12-VESPA 2003). Drug Alcohol Depend. 2006 Apr;82 Suppl 1:S71-9. doi: 10.1016/s0376-8716(06)80012-8. PMID: 16769450.  Roux P, Lions C, Cohen J, et al. Impact of HCV treatment and depressive symptoms on adherence to HAART among HIV-HCV-coinfected patients: results from the ANRS-CO13-HEPAVIH cohort. *Antivir Ther*. 2014;19(2):171-178. doi:10.3851/IMP2699  Schensul JJ, Ha T, Schensul S, Sarna A, Bryant K. Identifying the Intersection of Alcohol, Adherence and Sex in HIV Positive Men on ART Treatment in India Using an Adapted Timeline Followback Procedure. *AIDS Behav*. 2017;21(Suppl 2):228-242. doi:10.1007/s10461-017-1916-1  Suonpera, E., Matthews, R., Milinkovic, A. *et al.* Risky Alcohol Consumption and Associated Health Behaviour Among HIV-Positive and HIV-Negative Patients in a UK Sexual Health and HIV Clinic: A Cross-Sectional Questionnaire Study. *AIDS Behav* 24, 1717–1726 (2020). <https://doi.org/10.1007/s10461-019-02714-2>  Tang, A.M., Hamunime, N., Adams, R.A. *et al.* Introduction of an Alcohol-Related Electronic Screening and Brief Intervention (eSBI) Program to Reduce Hazardous Alcohol Consumption in Namibia’s Antiretroviral Treatment (ART) Program. *AIDS Behav* 23, 3078–3092 (2019). <https://doi.org/10.1007/s10461-019-02648-9>  Teixeira C, Dourado Mde L, Santos MP, Brites C. Impact of use of alcohol and illicit drugs by AIDS patients on adherence to antiretroviral therapy in Bahia, Brazil. AIDS Res Hum Retroviruses. 2013 May;29(5):799-804. doi: 10.1089/aid.2012.0296. Epub 2013 Feb 5. PMID: 23294471.  Vieira-Castro ACM, Oliveira LCM. Impact of alcohol consumption among patients in hepatitis C virus treatment. Arq Gastroenterol. 2017 Jul-Sept;54(3):232-237. doi: 10.1590/S0004-2803.201700000-33. Epub 2017 Jul 13. PMID: 28724048.  **References for tobacco and medication non-adherence**  Aye, W. L., Puckpinyo, A., & Peltzer, K. (2017). Non-adherence to anti-retroviral therapy among HIV infected adults in Mon State of Myanmar. *BMC public health*, *17*(1), 391. https://doi.org/10.1186/s12889-017-4309-5  Batista, J. d., Albuquerque, M., Santos, M. L., Miranda-Filho, D., Lacerda, H. R., Maruza, M., Moura, L. V., Coimbra, I., & Ximenes, R. A. (2014). Association between smoking, crack cocaine abuse and the discontinuation of combination antiretroviral therapy in Recife, Pernambuco, Brazil. *Revista do Instituto de Medicina Tropical de Sao Paulo*, *56*(2), 127–132. https://doi.org/10.1590/S0036-46652014000200007  Cioe, P. A., Gamarel, K. E., Pantalone, D. W., Monti, P. M., Mayer, K. H., & Kahler, C. W. (2017). Cigarette Smoking and Antiretroviral Therapy (ART) Adherence in a Sample of Heavy Drinking HIV-Infected Men Who Have Sex with Men (MSM). *AIDS and behavior*, *21*(7), 1956–1963. https://doi.org/10.1007/s10461-016-1496-5  Cohn, S. E., Umbleja, T., Mrus, J., Bardeguez, A. D., Andersen, J. W., & Chesney, M. A. (2008). Prior illicit drug use and missed prenatal vitamins predict nonadherence to antiretroviral therapy in pregnancy: adherence analysis A5084. *AIDS patient care and STDs*, *22*(1), 29–40. https://doi.org/10.1089/apc.2007.0053  Degroote, S., Vogelaers, D., Vermeir, P., Mariman, A., De Rick, A., Van Der Gucht, B., Pelgrom, J., Van Wanzeele, F., Verhofstede, C., Vancauwenberghe, J., & Vandijck, D. (2014). Determinants of adherence in a cohort of Belgian HIV patients: a pilot study. *Acta clinica Belgica*, *69*(2), 111–115. https://doi.org/10.1179/0001551214Z.00000000035  King, D., Grasso, C., Dant, L., Elsesser, S. A., Crane, H. M., Cropsey, K. L., Mayer, K. H., & O'Cleirigh, C. (2018). Treatment Outcomes Associated with Quitting Cigarettes Among Sexual Minority Men Living with HIV: Antiretroviral Adherence, Engagement in Care, and Sustained HIV RNA Suppression. *AIDS and behavior*, *22*(9), 2868–2876. https://doi.org/10.1007/s10461-018-2116-3  Mellins, C. A., Chu, C., Malee, K., Allison, S., Smith, R., Harris, L., Higgins, A., Zorrilla, C., Landesman, S., Serchuck, L., & Larussa, P. (2008). Adherence to antiretroviral treatment among pregnant and postpartum HIV-infected women. *AIDS care*, *20*(8), 958–968. https://doi.org/10.1080/09540120701767208  Murri, R., Guaraldi, G., Lupoli, P., Crisafulli, R., Marcotullio, S., von Schloesser, F., & Wu, A. W. (2009). Rate and predictors of self-chosen drug discontinuations in highly active antiretroviral therapy-treated HIV-positive individuals. *AIDS patient care and STDs*, *23*(1), 35–39. https://doi.org/10.1089/apc.2007.0248  Nguyen, N. T., Tran, B. X., Hwang, L. Y., Markham, C. M., Swartz, M. D., Vidrine, J. I., Phan, H. T., Latkin, C. A., & Vidrine, D. J. (2016). Effects of cigarette smoking and nicotine dependence on adherence to antiretroviral therapy among HIV-positive patients in Vietnam. *AIDS care*, *28*(3), 359–364. https://doi.org/10.1080/09540121.2015.1090535  Nolan, S., Walley, A. Y., Heeren, T. C., Patts, G. J., Ventura, A. S., Sullivan, M. M., Samet, J. H., & Saitz, R. (2017). HIV-infected individuals who use alcohol and other drugs, and virologic suppression. *AIDS care*, *29*(9), 1129–1136. https://doi.org/10.1080/09540121.2017.1327646  Ompad, D. C., Kingdon, M., Kupprat, S., Halkitis, S. N., Storholm, E. D., & Halkitis, P. N. (2014). Smoking and HIV-related health issues among older HIV-positive gay, bisexual, and other men who have sex with men. *Behavioral medicine (Washington, D.C.)*, *40*(3), 99–107. https://doi.org/10.1080/08964289.2014.889067  Sharma, S., Khadga, P., Dhungana, G. P., & Chitrakar, U. (2013). Medication adherence to antiretroviral therapy among patients visiting antiretroviral therapy center at Tribhuvan University Teaching Hospital, Kathmandu Nepal. *Kathmandu University medical journal (KUMJ)*, *11*(41), 50–53. https://doi.org/10.3126/kumj.v11i1.11027  Soares, R., Brito, A. M., Lima, K., & Lapa, T. M. (2019). Adherence to antiretroviral therapy among people living with HIV/AIDS in northeastern Brazil: a cross-sectional study. *Sao Paulo medical journal = Revista paulista de medicina*, *137*(6), 479–485. https://doi.org/10.1590/1516-3180.2019.0212170919  Spire, B., Duran, S., Souville, M., Leport, C., Raffi, F., Moatti, J. P., & APROCO cohort study group (2002). Adherence to highly active antiretroviral therapies (HAART) in HIV-infected patients: from a predictive to a dynamic approach. *Social science & medicine (1982)*, *54*(10), 1481–1496. https://doi.org/10.1016/s0277-9536(01)00125-3  **References for opioids and medication non-adherence**  Azar, P., Wood, E., Nguyen, P., Luma, M., Montaner, J., Kerr, T., & Milloy, M. J. (2015). Drug use patterns associated with risk of non-adherence to antiretroviral therapy among HIV-positive illicit drug users in a Canadian setting: a longitudinal analysis. *BMC infectious diseases*, *15*, 193. https://doi.org/10.1186/s12879-015-0913-0  Cohn, S. E., Jiang, H., McCutchan, J. A., Koletar, S. L., Murphy, R. L., Robertson, K. R., de St Maurice, A. M., Currier, J. S., & Williams, P. L. (2011). Association of ongoing drug and alcohol use with non-adherence to antiretroviral therapy and higher risk of AIDS and death: results from ACTG 362. *AIDS care*, *23*(6), 775–785. https://doi.org/10.1080/09540121.2010.525617 (study 185)  Culbert, G. J., Waluyo, A., Wang, M., Putri, T. A., Bazazi, A. R., & Altice, F. L. (2019). Adherence to Antiretroviral Therapy Among Incarcerated Persons with HIV: Associations with Methadone and Perceived Safety. *AIDS and behavior*, *23*(8), 2048–2058. https://doi.org/10.1007/s10461-018-2344-6  Cunningham, E. B., Hajarizadeh, B., Amin, J., Litwin, A. H., Gane, E., Cooper, C., Lacombe, K., Hellard, M., Read, P., Powis, J., Dalgard, O., Bruneau, J., Matthews, G. V., Feld, J. J., Dillon, J. F., Shaw, D., Bruggmann, P., Conway, B., Fraser, C., Marks, P., … SIMPLIFY and D3FEAT study groups (2020). Adherence to Once-daily and Twice-daily Direct-acting Antiviral Therapy for Hepatitis C Infection Among People With Recent Injection Drug Use or Current Opioid Agonist Therapy. *Clinical infectious diseases : an official publication of the Infectious Diseases Society of America*, *71*(7), e115–e124. <https://doi.org/10.1093/cid/ciz1089>  Ingersoll K. (2004). The impact of psychiatric symptoms, drug use, and medication regimen on non-adherence to HIV treatment. *AIDS care*, *16*(2), 199–211. https://doi.org/10.1080/09540120410001641048  Lambers, F. A., Stolte, I. G., van den Berg, C. H., Coutinho, R. A., & Prins, M. (2011). Harm reduction intensity-Its role in HAART adherence amongst drug users in Amsterdam. *The International journal on drug policy*, *22*(3), 210–218. https://doi.org/10.1016/j.drugpo.2011.02.004  Mimiaga, M. J., Reisner, S. L., Grasso, C., Crane, H. M., Safren, S. A., Kitahata, M. M., Schumacher, J. E., Mathews, W. C., & Mayer, K. H. (2013). Substance use among HIV-infected patients engaged in primary care in the United States: findings from the Centers for AIDS Research Network of Integrated Clinical Systems cohort. *American journal of public health*, *103*(8), 1457–1467. https://doi.org/10.2105/AJPH.2012.301162  Nolan, S., Walley, A. Y., Heeren, T. C., Patts, G. J., Ventura, A. S., Sullivan, M. M., Samet, J. H., & Saitz, R. (2017). HIV-infected individuals who use alcohol and other drugs, and virologic suppression. *AIDS care*, *29*(9), 1129–1136. https://doi.org/10.1080/09540121.2017.1327646  Palepu, A., Tyndall, M. W., Joy, R., Kerr, T., Wood, E., Press, N., Hogg, R. S., & Montaner, J. S. (2006). Antiretroviral adherence and HIV treatment outcomes among HIV/HCV co-infected injection drug users: the role of methadone maintenance therapy. *Drug and alcohol dependence*, *84*(2), 188–194. https://doi.org/10.1016/j.drugalcdep.2006.02.003  Paschen-Wolff, M. M., Campbell, A., Tross, S., Choo, T. H., Pavlicova, M., & Jarlais, D. D. (2020). DSM-5 substance use disorder symptom clusters and HIV antiretroviral therapy (ART) adherence. *AIDS care*, *32*(5), 645–650. https://doi.org/10.1080/09540121.2019.1686600  Shannon, K., Kerr, T., Lai, C., Ishida, T., Wood, E., Montaner, J. S., Hogg, R. S., & Tyndall, M. W. (2005). Nonadherence to antiretroviral therapy among a community with endemic rates of injection drug use. *Journal of the International Association of Physicians in AIDS Care (Chicago, Ill. : 2002)*, *4*(3), 66–72. https://doi.org/10.1177/1545109705284353  **References for stimulants and medication non-adherence**  Cohen MH, Cook JA, Grey D, et al. Medically eligible women who do not use HAART: the importance of abuse, drug use, and race. *Am J Public Health*. 2004;94(7):1147-1151. doi:10.2105/ajph.94.7.1147  De Boni RB, Shepherd BE, Grinsztejn B, Cesar C, Cortés C, Padgett D, Gotuzzo E, Belaunzarán-Zamudio PF, Rebeiro PF, Duda SN, McGowan CC. Substance Use and Adherence Among People Living with HIV/AIDS Receiving cART in Latin America. AIDS Behav. 2016 Nov;20(11):2692-2699. doi: 10.1007/s10461-016-1398-6. PMID: 27091028; PMCID: PMC5069110.  Hinkin CH, Barclay TR, Castellon SA, Levine AJ, Durvasula RS, Marion SD, Myers HF, Longshore D. Drug use and medication adherence among HIV-1 infected individuals. AIDS Behav. 2007 Mar;11(2):185-94. doi: 10.1007/s10461-006-9152-0. PMID: 16897351; PMCID: PMC2867605.  Ingersoll K. The impact of psychiatric symptoms, drug use, and medication regimen on non-adherence to HIV treatment. AIDS Care. 2004 Feb;16(2):199-211. doi: 10.1080/09540120410001641048. PMID: 14676026.  King RM, Vidrine DJ, Danysh HE, Fletcher FE, McCurdy S, Arduino RC, Gritz ER. Factors associated with nonadherence to antiretroviral therapy in HIV-positive smokers. AIDS Patient Care STDS. 2012 Aug;26(8):479-85. doi: 10.1089/apc.2012.0070. Epub 2012 May 21. PMID: 22612468; PMCID: PMC3407390.  Lucas GM, Gebo KA, Chaisson RE, Moore RD. Longitudinal assessment of the effects of drug and alcohol abuse on HIV-1 treatment outcomes in an urban clinic. AIDS. 2002 Mar 29;16(5):767-74. doi: 10.1097/00002030-200203290-00012. PMID: 11964533.  Roux P, Lions C, Cohen J, et al. Impact of HCV treatment and depressive symptoms on adherence to HAART among HIV-HCV-coinfected patients: results from the ANRS-CO13-HEPAVIH cohort. *Antivir Ther*. 2014;19(2):171-178. doi:10.3851/IMP2699  Tucker JS, Burnam MA, Sherbourne CD, Kung FY, Gifford AL. Substance use and mental health correlates of nonadherence to antiretroviral medications in a sample of patients with human immunodeficiency virus infection. Am J Med. 2003 May;114(7):573-80. doi: 10.1016/s0002-9343(03)00093-7. PMID: 12753881.  **References for depression and medication non-adherence**  Adeoti AO, Dada M, Elebiyo T, Fadare J, Ojo O. Survey of antiretroviral therapy adherence and predictors of poor adherence among HIV patients in a tertiary institution in Nigeria. Pan Afr Med J. 2019 Jul 31;33:277. doi: 10.11604/pamj.2019.33.277.18711. PMID: 31692880; PMCID: PMC6815489.  Camargo CC, Cavassan NRV, Tasca KI, Meneguin S, Miot HA, Souza LR. Depression and Coping Are Associated with Failure of Adherence to Antiretroviral Therapy Among People Living with HIV/AIDS. AIDS Res Hum Retroviruses. 2019 Nov/Dec;35(11-12):1181-1188. doi: 10.1089/aid.2019.0050. Epub 2019 Oct 8. PMID: 31592722.  Do, H.M., Dunne, M.P., Kato, M. *et al.* Factors associated with suboptimal adherence to antiretroviral therapy in Viet Nam: a cross-sectional study using audio computer-assisted self-interview (ACASI). *BMC Infect Dis* 13, 154 (2013). <https://doi.org/10.1186/1471-2334-13-154>  Gebrezgabher, B.B., Kebede, Y., Kindie, M. *et al.* Determinants to antiretroviral treatment non-adherence among adult HIV/AIDS patients in northern Ethiopia. *AIDS Res Ther* 14, 16 (2017). <https://doi.org/10.1186/s12981-017-0143-1>  Kinyanda E, Nakasujja N, Levin J, Birabwa H, Mpango R, Grosskurth H, Seedat S, Patel V. Major depressive disorder and suicidality in early HIV infection and its association with risk factors and negative outcomes as seen in semi-urban and rural Uganda. J Affect Disord. 2017 Apr 1;212:117-127. doi: 10.1016/j.jad.2017.01.033. Epub 2017 Jan 23. PMID: 28160684.  Letta S, Demissie A, Oljira L, Dessie Y. Factors associated with adherence to Antiretroviral Therapy (ART) among adult people living with HIV and attending their clinical care, Eastern Ethiopia. BMC Int Health Hum Rights. 2015 Dec 28;15:33. doi: 10.1186/s12914-015-0071-x. Erratum in: BMC Int Health Hum Rights. 2016;16:8. PMID: 26711659; PMCID: PMC4693416.  Li L, Lee SJ, Wen Y, Lin C, Wan D, Jiraphongsa C. Antiretroviral therapy adherence among patients living with HIV/AIDS in Thailand. Nurs Health Sci. 2010 Jun;12(2):212-20. doi: 10.1111/j.1442-2018.2010.00521.x. PMID: 20602694; PMCID: PMC2947817.  Memiah P, Shumba C, Etienne-Mesubi M, Agbor S, Hossain MB, Komba P, Niyang M, Biadgilign S. The effect of depressive symptoms and CD4 count on adherence to highly active antiretroviral therapy in sub-Saharan Africa. J Int Assoc Provid AIDS Care. 2014 Jul-Aug;13(4):346-52. doi: 10.1177/2325957413503368. PMID: 24114726.  Mohammed H, Kieltyka L, Richardson-Alston G, Magnus M, Fawal H, Vermund SH, Rice J, Kissinger P. Adherence to HAART among HIV-infected persons in rural Louisiana. AIDS Patient Care STDS. 2004 May;18(5):289-96. doi: 10.1089/108729104323076025. PMID: 15186712.  Moraes RP, Casseb J. Depression and adherence to antiretroviral treatment in HIV-positive men in São Paulo, the largest city in South America: Social and psychological implications. Clinics (Sao Paulo). 2017 Dec;72(12):743-749. doi: 10.6061/clinics/2017(12)05. PMID: 29319720; PMCID: PMC5738567.  Onono M, Odwar T, Abuogi L, Owuor K, Helova A, Bukusi E, Turan J, Hampanda K. Effects of Depression, Stigma and Intimate Partner Violence on Postpartum Women's Adherence and Engagement in HIV Care in Kenya. AIDS Behav. 2020 Jun;24(6):1807-1815. doi: 10.1007/s10461-019-02750-y. PMID: 31813076; PMCID: PMC7228848.  Royal SW, Kidder DP, Patrabansh S, Wolitski RJ, Holtgrave DR, Aidala A, Pals S, Stall R. Factors associated with adherence to highly active antiretroviral therapy in homeless or unstably housed adults living with HIV. AIDS Care. 2009 Apr;21(4):448-55. doi: 10.1080/09540120802270250. PMID: 19401865.  Tao J, Qian HZ, Kipp AM, Ruan Y, Shepherd BE, Amico KR, Shao Y, Lu H, Vermund SH. Effects of depression and anxiety on antiretroviral therapy adherence among newly diagnosed HIV-infected Chinese MSM. AIDS. 2017 Jan 28;31(3):401-406. doi: 10.1097/QAD.0000000000001287. PMID: 27677168; PMCID: PMC5233466.  Wagner GJ, Ghosh-Dastidar B, Mukasa B, Linnemayr S. Changes in ART Adherence Relate to Changes in depression as Well! Evidence for the Bi-directional Longitudinal Relationship Between Depression and ART Adherence from a Prospective Study of HIV Clients in Uganda. AIDS Behav. 2020 Jun;24(6):1816-1824. doi: 10.1007/s10461-019-02754-8. PMID: 31813077; PMCID: PMC7228829.  Yu, Y., Luo, D., Chen, X. *et al.* Medication adherence to antiretroviral therapy among newly treated people living with HIV. *BMC Public Health* 18, 825 (2018). <https://doi.org/10.1186/s12889-018-5731-z>  **References for anxiety and medication non-adherence**  Adejumo O, Oladeji B, Akpa O, et al. Psychiatric disorders and adherence to antiretroviral therapy among a population of HIV-infected adults in Nigeria. *Int J STD AIDS*. 2016;27(11):938-949. doi:10.1177/0956462415600582  Adeoti AO, Dada M, Elebiyo T, Fadare J, Ojo O. Survey of antiretroviral therapy adherence and predictors of poor adherence among HIV patients in a tertiary institution in Nigeria. Pan Afr Med J. 2019 Jul 31;33:277. doi: 10.11604/pamj.2019.33.277.18711. PMID: 31692880; PMCID: PMC6815489.  Been SK, Schadé A, Bassant N, Kastelijns M, Pogány K, Verbon A. Anxiety, depression and treatment adherence among HIV-infected migrants. AIDS Care. 2019 Aug;31(8):979-987. doi: 10.1080/09540121.2019.1601676. Epub 2019 Apr 8. PMID: 30957530.  Guimarães MD, Rocha GM, Campos LN, de Freitas FM, Gualberto FA, Teixeira Rd, de Castilho FM. Difficulties reported by HIV-infected patients using antiretroviral therapy in Brazil. Clinics (Sao Paulo). 2008 Apr;63(2):165-72. doi: 10.1590/s1807-59322008000200003. PMID: 18438569; PMCID: PMC2664217.  Ingersoll K. The impact of psychiatric symptoms, drug use, and medication regimen on non-adherence to HIV treatment. AIDS Care. 2004 Feb;16(2):199-211. doi: 10.1080/09540120410001641048. PMID: 14676026.  Joshi B, Chauhan S, Pasi A, Kulkarni R, Sunil N, Bachani D, Mankeshwar R; ART adherence study group. Level of suboptimal adherence to first line antiretroviral treatment & its determinants among HIV positive people in India. Indian J Med Res. 2014 Jul;140(1):84-95. PMID: 25222782; PMCID: PMC4181166.  Nel A, Kagee A. The relationship between depression, anxiety and medication adherence among patients receiving antiretroviral treatment in South Africa. AIDS Care. 2013 Aug;25(8):948-55. doi: 10.1080/09540121.2012.748867. Epub 2012 Dec 11. PMID: 23231527.  Nguyen NT, Tran BX, Hwang LY, Markham CM, Swartz MD, Vidrine JI, Phan HT, Latkin CA, Vidrine DJ. Effects of cigarette smoking and nicotine dependence on adherence to antiretroviral therapy among HIV-positive patients in Vietnam. AIDS Care. 2016;28(3):359-64. doi: 10.1080/09540121.2015.1090535. Epub 2015 Oct 13. PMID: 26461976; PMCID: PMC6691494.  Nilsson Schönnesson L, Williams ML, Ross MW, Bratt G, Keel B. Factors associated with suboptimal antiretroviral therapy adherence to dose, schedule, and dietary instructions. AIDS Behav. 2007 Mar;11(2):175-83. doi: 10.1007/s10461-006-9160-0. PMID: 16927178.  Pokhrel KN, Pokhrel KG, Sharma VD, Poudel KC, Neupane SR, Mlunde LB, Jimba M. Mental health disorders and substance use among people living with HIV in Nepal: their influence on non-adherence to anti-retroviral therapy. AIDS Care. 2019 Aug;31(8):923-931. doi: 10.1080/09540121.2019.1587365. Epub 2019 Mar 5. PMID: 30835503.  Prasithsirikul W, Chongthawonsatid S, Ohata PJ, Keadpudsa S, Klinbuayaem V, Rerksirikul P, Kerr SJ, Ruxrungtham K, Ananworanich J, Avihingsanon A; PROGRESS study team. Depression and anxiety were low amongst virally suppressed, long-term treated HIV-infected individuals enrolled in a public sector antiretroviral program in Thailand. AIDS Care. 2017 Mar;29(3):299-305. doi: 10.1080/09540121.2016.1201194. Epub 2016 Aug 10. PMID: 27683949.  Tucker JS, Burnam MA, Sherbourne CD, Kung FY, Gifford AL. Substance use and mental health correlates of nonadherence to antiretroviral medications in a sample of patients with human immunodeficiency virus infection. Am J Med. 2003 May;114(7):573-80. doi: 10.1016/s0002-9343(03)00093-7. PMID: 12753881.  Yu Y, Luo D, Chen X, Huang Z, Wang M, Xiao S. Medication adherence to antiretroviral therapy among newly treated people living with HIV. *BMC Public Health*. 2018;18(1):825. Published 2018 Jul 4. doi:10.1186/s12889-018-5731-z  **References for pain and medication non-adherence**  Denis, C. M., Morales, K. H., Wu, Q., Metzger, D. S., & Cheatle, M. D. (2019). Association Between Diagnoses of Chronic Noncancer Pain, Substance Use Disorder, and HIV-Related Outcomes in People Living With HIV. *Journal of acquired immune deficiency syndromes (1999)*, *82 Suppl 2*(2), S142–S147. https://doi.org/10.1097/QAI.0000000000002179  Johnson, M. O., Charlebois, E., Morin, S. F., Catz, S. L., Goldstein, R. B., Remien, R. H., Rotheram-Borus, M. J., Mickalian, J. D., Kittel, L., Samimy-Muzaffar, F., Lightfoot, M. A., Gore-Felton, C., Chesney, A., & NIMH Healthy Living Project Team (2005). Perceived adverse effects of antiretroviral therapy. *Journal of pain and symptom management*, *29*(2), 193–205. https://doi.org/10.1016/j.jpainsymman.2004.05.005  Merlin, J. S., Westfall, A. O., Raper, J. L., Zinski, A., Norton, W. E., Willig, J. H., Gross, R., Ritchie, C. S., Saag, M. S., & Mugavero, M. J. (2012). Pain, mood, and substance abuse in HIV: implications for clinic visit utilization, antiretroviral therapy adherence, and virologic failure. *Journal of acquired immune deficiency syndromes (1999)*, *61*(2), 164–170. https://doi.org/10.1097/QAI.0b013e3182662215  Nguyen, N. T., Tran, B. X., Hwang, L. Y., Markham, C. M., Swartz, M. D., Vidrine, J. I., Phan, H. T., Latkin, C. A., & Vidrine, D. J. (2016). Effects of cigarette smoking and nicotine dependence on adherence to antiretroviral therapy among HIV-positive patients in Vietnam. *AIDS care*, *28*(3), 359–364. https://doi.org/10.1080/09540121.2015.1090535  Shah, B., Walshe, L., Saple, D. G., Mehta, S. H., Ramnani, J. P., Kharkar, R. D., Bollinger, R. C., & Gupta, A. (2007). Adherence to antiretroviral therapy and virologic suppression among HIV-infected persons receiving care in private clinics in Mumbai, India. *Clinical infectious diseases : an official publication of the Infectious Diseases Society of America*, *44*(9), 1235–1244. https://doi.org/10.1086/513429  **References for alcohol and unprotected sex**  Bajunirwe F, Bangsberg DR, Sethi AK. Alcohol use and HIV serostatus of partner predict high-risk sexual behavior among patients receiving antiretroviral therapy in South Western Uganda. BMC Public Health. 2013 May 3;13:430. doi: 10.1186/1471-2458-13-430. PMID: 23641795; PMCID: PMC3645971.  Certain HE, Harahan BJ, Saewyc EM, Fleming MF. Condom use in heavy drinking college students: the importance of always using condoms. J Am Coll Health. 2009 Nov-Dec;58(3):187-94. doi: 10.1080/07448480903295284. PMID: 19959432; PMCID: PMC2789340.  Coldiron ME, Stephenson R, Chomba E, Vwalika C, Karita E, Kayitenkore K, Tichacek A, Isanhart L, Allen S, Haworth A. The relationship between alcohol consumption and unprotected sex among known HIV-discordant couples in Rwanda and Zambia. AIDS Behav. 2008 Jul;12(4):594-603. doi: 10.1007/s10461-007-9304-x. Epub 2007 Aug 18. Erratum in: AIDS Behav. 2008 Jul;12(4):604. PMID: 17705032.  Goodman-Meza D, Pitpitan EV, Semple SJ, Wagner KD, Chavarin CV, Strathdee SA, Patterson TL. Hazardous drinking and HIV-risk-related behavior among male clients of female sex workers in Tijuana, Mexico. Am J Addict. 2014 Sep-Oct;23(5):502-9. doi: 10.1111/j.1521-0391.2014.12138.x. Epub 2014 Jul 25. PMID: 25066863; PMCID: PMC4427019.  Herrera MC, Konda KA, Leon SR, Deiss R, Brown B, Calvo GM, Salvatierra HJ, Caceres CF, Klausner JD. Impact of alcohol use on sexual behavior among men who have sex with men and transgender women in Lima, Peru. Drug Alcohol Depend. 2016 Apr 1;161:147-54. doi: 10.1016/j.drugalcdep.2016.01.030. Epub 2016 Feb 16. PMID: 26896169; PMCID: PMC4807690.  Hutton HE, Lesko CR, Li X, et al. Alcohol Use Patterns and Subsequent Sexual Behaviors Among Women, Men who have Sex with Men and Men who have Sex with Women Engaged in Routine HIV Care in the United States. *AIDS Behav*. 2019;23(6):1634-1646. doi:10.1007/s10461-018-2337-5  Kahler CW, Wray TB, Pantalone DW, et al. Daily associations between alcohol use and unprotected anal sex among heavy drinking HIV-positive men who have sex with men. *AIDS Behav*. 2015;19(3):422-430. doi:10.1007/s10461-014-0896-7  Kerridge BT, Castor D, Tran P, Barnhart M, Pickering R. Association between intoxication at last sexual intercourse and unprotected sex among men and women in Uganda. *J Infect Dev Ctries*. 2014;8(11):1461-1469. Published 2014 Nov 13. doi:10.3855/jidc.4832  Liu Y, Ruan Y, Strauss SM, Yin L, Liu H, Amico KR, Zhang C, Shao Y, Qian HZ, Vermund SH. Alcohol misuse, risky sexual behaviors, and HIV or syphilis infections among Chinese men who have sex with men. Drug Alcohol Depend. 2016 Nov 1;168:239-246. doi: 10.1016/j.drugalcdep.2016.09.020. Epub 2016 Sep 30. PMID: 27723554; PMCID: PMC5523945.  Luchters S, Geibel S, Syengo M, Lango D, King'ola N, Temmerman M, Chersich MF. Use of AUDIT, and measures of drinking frequency and patterns to detect associations between alcohol and sexual behaviour in male sex workers in Kenya. BMC Public Health. 2011 May 25;11:384. doi: 10.1186/1471-2458-11-384. PMID: 21609499; PMCID: PMC3128017.  Massaro LTS, Abdalla RR, Laranjeira R, Caetano R, Pinsky I, Madruga CS. Alcohol misuse among women in Brazil: recent trends and associations with unprotected sex, early pregnancy, and abortion. Braz J Psychiatry. 2019 Mar-Apr;41(2):131-137. doi: 10.1590/1516-4446-2017-0024. Epub 2018 Oct 22. PMID: 30365669; PMCID: PMC6781687.  Metsch LR, Bell C, Pereyra M, Cardenas G, Sullivan T, Rodriguez A, Gooden L, Khoury N, Kuper T, Brewer T, del Rio C. Hospitalized HIV-infected patients in the era of highly active antiretroviral therapy. Am J Public Health. 2009 Jun;99(6):1045-9. doi: 10.2105/AJPH.2008.139931. Epub 2009 Apr 16. PMID: 19372520; PMCID: PMC2679801.  Muñoz-Laboy M, Martínez O, Guilamo-Ramos V, Draine J, Garg KE, Levine E, Ripkin A. Influences of Economic, Social and Cultural Marginalization on the Association Between Alcohol Use and Sexual Risk Among Formerly Incarcerated Latino Men. J Immigr Minor Health. 2017 Oct;19(5):1073-1087. doi: 10.1007/s10903-017-0554-z. PMID: 28197862; PMCID: PMC5557709.  Nkosi S, Sikweyiya Y, Kekwaletswe CT, Morojele NK. Male circumcision, alcohol use and unprotected sex among patrons of bars and taverns in rural areas of North-West province, South Africa. AIDS Care. 2015;27(5):612-7. doi: 10.1080/09540121.2014.983040. Epub 2014 Nov 27. PMID: 25428332.  Raj A, Reed E, Santana MC, Walley AY, Welles SL, Horsburgh CR, Flores SA, Silverman JG. The associations of binge alcohol use with HIV/STI risk and diagnosis among heterosexual African American men. Drug Alcohol Depend. 2009 Apr 1;101(1-2):101-6. doi: 10.1016/j.drugalcdep.2008.11.008. Epub 2008 Dec 30. PMID: 19117698.  Stein MD, Anderson B, Charuvastra A, Friedmann PD. Alcohol use and sexual risk taking among hazardously drinking drug injectors who attend needle exchange. Alcohol Clin Exp Res. 2001 Oct;25(10):1487-93. PMID: 11696669.  Theall KP, Clark RA, Powell A, Smith H, Kissinger P. Alcohol consumption, ART usage and high-risk sex among women infected with HIV. AIDS Behav. 2007 Mar;11(2):205-15. doi: 10.1007/s10461-006-9159-6. PMID: 16897350.  Tran BR, Davis A, Ito SI, Matchere F, Reader E, Nkhoma V, Grillo M, Banda AC. Alcohol and Cannabis Use and Sexual Risk Behaviors in the Malawi Defence Force. AIDS Behav. 2018 Sep;22(9):2851-2860. doi: 10.1007/s10461-018-2167-5. PMID: 29869734.  Wand H, Ward J, Bryant J, Delaney-Thiele D, Worth H, Pitts M, Kaldor JM. Individual and population level impacts of illicit drug use, sexual risk behaviours on sexually transmitted infections among young Aboriginal and Torres Strait Islander people: results from the GOANNA survey. BMC Public Health. 2016 Jul 19;16:600. doi: 10.1186/s12889-016-3195-6. PMID: 27435166; PMCID: PMC4950619.  Weiser SD, Leiter K, Heisler M, McFarland W, Percy-de Korte F, DeMonner SM, Tlou S, Phaladze N, Iacopino V, Bangsberg DR. A population-based study on alcohol and high-risk sexual behaviors in Botswana. PLoS Med. 2006 Oct;3(10):e392. doi: 10.1371/journal.pmed.0030392. PMID: 17032060; PMCID: PMC1592342.  White D, Wilson KS, Masese LN, Wanje G, Jaoko W, Mandaliya K, Richardson BA, Kinuthia J, Simoni JM, McClelland RS. Alcohol Use and Associations With Biological Markers and Self-Reported Indicators of Unprotected Sex in Human Immunodeficiency Virus-Positive Female Sex Workers in Mombasa, Kenya. Sex Transm Dis. 2016 Oct;43(10):642-7. doi: 10.1097/OLQ.0000000000000502. PMID: 27631360; PMCID: PMC5026390.  Wirtz AL, Zelaya CE, Latkin C, et al. Alcohol Use and Associated Sexual and Substance Use Behaviors Among Men Who Have Sex with Men in Moscow, Russia. *AIDS Behav*. 2016;20(3):523-536. doi:10.1007/s10461-015-1066-2  **References for tobacco and unprotected sex**  Al Rifai, R., Nakamura, K., Seino, K., Kizuki, M., & Morita, A. (2015). Unsafe sexual behaviour in domestic and foreign migrant male workers in multinational workplaces in Jordan: occupational-based and behavioural assessment survey. *BMJ open*, *5*(6), e007703. <https://doi.org/10.1136/bmjopen-2015-007703>  Barros, F. C., Melo, A. P., Cournos, F., Cherchiglia, M. L., Peixoto, E. R., & Guimarães, M. D. (2014). Cigarette smoking among psychiatric patients in Brazil. *Cadernos de saude publica*, *30*(6), 1195–1206. <https://doi.org/10.1590/0102-311x00027113>  Chen, R., Tao, F., Ma, Y., Zhong, L., Qin, X., & Hu, Z. (2014). Associations between social support and condom use among commercial sex workers in China: a cross-sectional study. *PloS one*, *9*(12), e113794. <https://doi.org/10.1371/journal.pone.0113794>  Choi, E. P., Wong, J. Y., Lo, H. H., Wong, W., Chio, J. H., & Fong, D. Y. (2016). The Impacts of Using Smartphone Dating Applications on Sexual Risk Behaviours in College Students in Hong Kong. *PloS one*, *11*(11), e0165394. <https://doi.org/10.1371/journal.pone.0165394>  Clark, T. C., Crengle, S., Sheridan, J., Rowe, D., & Robinson, E. (2014). Factors associated with consistent contraception and condom use among Māori secondary school students in New Zealand. *Journal of paediatrics and child health*, *50*(4), 258–265. <https://doi.org/10.1111/jpc.12450>  Edelman, N., Cassell, J. A., de Visser, R., Prah, P., & Mercer, C. H. (2017). Can psychosocial and socio-demographic questions help identify sexual risk among heterosexually-active women of reproductive age? Evidence from Britain's third National Survey of Sexual Attitudes and Lifestyles (Natsal-3). *BMC public health*, *17*(1), 5. <https://doi.org/10.1186/s12889-016-3918-8>  Everett, S. A., Malarcher, A. M., Sharp, D. J., Husten, C. G., & Giovino, G. A. (2000). Relationship between cigarette, smokeless tobacco, and cigar use, and other health risk behaviors among U.S. high school students. *The Journal of school health*, *70*(6), 234–240. <https://doi.org/10.1111/j.1746-1561.2000.tb07424.x>  Graves, K. L., & Leigh, B. C. (1995). The relationship of substance use to sexual activity among young adults in the United States. *Family planning perspectives*, *27*(1), 18–33  Hale, D. R., & Viner, R. M. (2016). The correlates and course of multiple health risk behaviour in adolescence. *BMC public health*, *16*, 458. <https://doi.org/10.1186/s12889-016-3120-z>  Hong, E., & Kang, Y. (2017). Gender Differences in Sexual Behaviors in Korean Adolescents. *Journal of pediatric nursing*, *37*, e16–e22. <https://doi.org/10.1016/j.pedn.2017.08.020>  Kalina, O., Geckova, A. M., Jarcuska, P., Orosova, O., van Dijk, J. P., & Reijneveld, S. A. (2009). Psychological and behavioural factors associated with sexual risk behaviour among Slovak students. *BMC public health*, *9*, 15. <https://doi.org/10.1186/1471-2458-9-15>  Lam, T. H., Stewart, S. M., & Ho, L. M. (2001). Smoking and high-risk sexual behavior among young adults in Hong Kong. *Journal of behavioral medicine*, *24*(5), 503–518. <https://doi.org/10.1023/a:1012227728232>  Li, S., Huang, H., Xu, G., Cai, Y., Huang, F., & Ye, X. (2013). Substance use, risky sexual behaviors, and their associations in a Chinese sample of senior high school students. *BMC public health*, *13*, 295. <https://doi.org/10.1186/1471-2458-13-295>  Novak, D. P., & Karlsson, R. B. (2005). Gender differed factors affecting male condom use. A population-based study of 18-year-old Swedish adolescents. *International journal of adolescent medicine and health*, *17*(4), 379–390. <https://doi.org/10.1515/ijamh.2005.17.4.379>  Nyembezi, A., Sifunda, S., Funani, I., Ruiter, R. A., Van Den Borne, B., & Reddy, P. S. (2009). Correlates of risky sexual behaviors in recently traditionally circumcised men from initiation lodges in the Eastern Cape, South Africa. *International quarterly of community health education*, *30*(2), 97–114. <https://doi.org/10.2190/IQ.30.2.b>  Oppong Asante, K., Meyer-Weitz, A., & Petersen, I. (2014). Substance use and risky sexual behaviours among street connected children and youth in Accra, Ghana. *Substance abuse treatment, prevention, and policy*, *9*, 45. <https://doi.org/10.1186/1747-597X-9-45>  Pahl, K., Brook, D. W., Morojele, N. K., & Brook, J. S. (2010). Nicotine dependence and problem behaviors among urban South African adolescents. *Journal of behavioral medicine*, *33*(2), 101–109. <https://doi.org/10.1007/s10865-009-9242-3>  Parkes, A., Wight, D., Henderson, M., & Hart, G. (2007). Explaining associations between adolescent substance use and condom use. *The Journal of adolescent health : official publication of the Society for Adolescent Medicine*, *40*(2), 180.e1–180.e1818. https://doi.org/10.1016/j.jadohealth.2006.09.012  Peixoto, E. R., Barros, F. C., & Guimarães, M. D. (2014). Factors associated with unprotected sexual practice among men and women with mental illnesses in Brazil. *Cadernos de saude publica*, *30*(7), 1475–1486. <https://doi.org/10.1590/0102-311x00029313>  Peltzer, K., & Pengpid, S. (2016). Risk and Protective Factors Affecting Sexual Risk Behavior Among School-Aged Adolescents in Fiji, Kiribati, Samoa, and Vanuatu. *Asia-Pacific journal of public health*, *28*(5), 404–415. <https://doi.org/10.1177/1010539516650725>  Richter, D. L., Valois, R. F., McKeown, R. E., & Vincent, M. L. (1993). Correlates of condom use and number of sexual partners among high school adolescents. *The Journal of school health*, *63*(2), 91–96. <https://doi.org/10.1111/j.1746-1561.1993.tb06087.x>  Saingam, D., Assanangkornchai, S., & Geater, A. F. (2012). Drinking-smoking status and health risk behaviors among high school students in Thailand. *Journal of drug education*, *42*(2), 177–193. <https://doi.org/10.2190/DE.42.2.d>  Yan, A. F., Chiu, Y. W., Stoesen, C. A., & Wang, M. Q. (2007). STD-/HIV-related sexual risk behaviors and substance use among U.S. rural adolescents. *Journal of the National Medical Association*, *99*(12), 1386–1394  Yi, S., Te, V., Pengpid, S., & Peltzer, K. (2018). Social and behavioural factors associated with risky sexual behaviours among university students in nine ASEAN countries: a multi-country cross-sectional study. *SAHARA J : journal of Social Aspects of HIV/AIDS Research Alliance*, *15*(1), 71–79. <https://doi.org/10.1080/17290376.2018.1503967>  Young, H., Burke, L., & Nic Gabhainn, S. (2018). Sexual intercourse, age of initiation and contraception among adolescents in Ireland: findings from the Health Behaviour in School-aged Children (HBSC) Ireland study. *BMC public health*, *18*(1), 362. <https://doi.org/10.1186/s12889-018-5217-z>  **References for opioids and unprotected sex**  Binswanger, I.A., Mueller, S., Beaty, B.L., Min, S., Corsi, K.F. (2014) Gender and risk behavior for HIV and sexually transmitted infections among recently released inmates: A prospective cohort study. AIDS Care, 26(7), 872-881.  Buttram, M.E., Kurtz, S.P., Surratt, H.L., Levi-Minzi, M.A. (2014). Health and social problems associated with prescription opioid misuse among a diverse sample of high risk substance-using MSM. Subst Use Misuse, 49(3), 277-284.  Carney, T., Petersen Williams, P. M., Plüddemann, A., & Parry, C. D. (2015). Sexual HIV risk among substance-using female commercial sex workers in Durban, South Africa. *African journal of AIDS research : AJAR*, *14*(2), 153–158. https://doi.org/10.2989/16085906.2015.1040811  Castilla, J., Barrio, G., Belza, M.J., de la Fuenta, L. (1999). Drug and alcohol consumption and sexual risk behavior among young adults: results from a national survey. DAD, 56, 47-53.  Chen, Y., Issema, R.S., Khanna, A.S., Pho, M.T., Schneider, J.A., (2019). Prescription opioids use in a population-based sample of young black men who have sex with men: a longitudinal cohort study. Subst Use Misuse., 54(12)  Cohen, L.R., Tross, S., Pavilcova, M., Hu, M., Campbell, A.N., Nunes, E.V., (2009) Substance use, childhood sexual abuse, and sexual risk behavior among women in methadone treatment. Amer. J. of Drug and Alc. Abuse, 35, 305-310.  Gossop M, Marsden J, Stewart D, Treacy S. Reduced injection risk and sexual risk behaviours after drug misuse treatment: Results from national treatment outcome research study. AIDS Care., 2002; 14(1):77-93.  Lemons, A., DeGroote, N., Perez, A., Craw, J., Nyaky, M., Broz, D., Mattson, C.L., Beer, L. (2019). Opioid misuse among HIV-positive adults in medical care: results from medical monitoring project, 2009-2014. J Acquir Immune Defic Syndr., 80(2), 127-134.  McNall M, Remafedi G. Relationship of amphetamine and other substance use to unprotected intercourse among young men who have sex with men. Arch Pediatr Adolesc Med., 1990; 153:1130-1135.  Mimiaga, M.J., Reisner, S.L., Grasso, C., Crane, H.M., Safren, S.A., Kitahata, M.M., Schumacher, J.E., Mathews, C., Mayer, K.H. (2013). Substance use among HIV-infected patients engaged in primary care in the United States: findings from the centers for AIDS research network of integrated clinical systems cohort. Am J Public Health, 103, 1457-1467  Raj, A., Saitz, R., Cheng, D.M., Winter, M., Samet, J.H. (2007). Associations between alcohol, heroin, and cocoaine use and high risk sexual behaviors among detoxification patients. Am J. Drug and Alcohol Abuse, 33, 169-178.  Suohu, K., Humtsoe, C., Saggurti, N., Sabarwal, S., Manapartra, B., Kermode, M. (2012). Understanding the association between injecting and sexual risk behavior of injecting drug users in Manipur and Nagaland, India. Harm Reduction Journal, 9(40).  Tross, S. Hanner, J., Mei-Chen, H., Paclicova, M., Campbell, A., Nunes, E.V. (2009). Substance use and high risk secual behaviors among women in psychosocial outpatient and methadone maintenance treatment programs. Am J Drug Alcohol Abuse, 35(5), 368-374.  Zule, W.A., Orsmasionwu, C., Evon, D., Hino, S., Doherty, I.A., Bobashev, G.V., Wechsberg, W.M. (2016). Event-level analyses of sex risk and injection risk behaviors among nonmedical prescription opioid users. Am J Drug Alcohol Abuse, 42(6), 689-697.  **References for stimulants and unprotected sex**  Bailey, S.L., Camlin, C.S., Ennett, S.T. (1998) Substance use and risky sexual behavior among homeless and runaway youth. J Adolesc Health, 23(6), 378-388.  Boone, M.R., Cook, S.H., Wilson, P. (2013). Substance use and sexual risk behavior in HIV-positive men who have sex with men: An episode-level analysis. AID Behav., 17, 1883-1887  Borders, T.F., Stewart, K.E., Wright, P.B., Leukefeld, C., Falck, R.S., Carlson, R.G., Booth, B.M. (2013). Risk sex in rural America: Longitudinal changes in a community-based cohort of methamphetamine and cocaine users. Am J Addict. 22(6), 535-542.  Brewer, D.D., Golden, M.R., Handsfield, H. H. (2006). Unsafe sexual behavior and correlated of risk in a probability sample of men who have sex with men in the era of highly active antiretroviral therapy. Sexually Transmitted Diseases, 33(4), 250-255  Brown, R.E., Turner, C., Hern, J., Santos, G. (2017). Partner-level substance use associated with increased sexual risk behaviors among men who have sex with men in San Francisco, CA. DAD, 176, 176-180.  Bruce, D., Bauermeister, J.A., Kahana, S.Y., Mendoza, E., Fernandez, M.I. (2018). Correlates of serodiscordant condomless anal intercourse among virologically detectable HIV-positive young men who have sex with men. AIDS Behav., 22(11), 3535-3539  Carey, J.W., Mejia, R., Bingham, T., Ciesielski, C., Gelaude, D., Herbst, J.H., Sinunu, M., Sey, E., Prachand, N., Jenkins, R.A., Stall, R. (2009). Drug use, high-risk sex behaviors, and increased risk for recent HIV infection among men who have sex with men in Chicago and Los Angeles. AIDS Behav., 13, 1084-1096  Castilla, J., Barrio, G., Belza, M.J., de la Fuenta, L. (1999). Drug and alcohol consumption and sexual risk behaviour among young adults: results from a national survey. DAD, 56, 47-53  CDC. (2006). Methamphetamine use and HIV risk behaviors among heterosexual men --- Preliminary results from five Northern California counties, December 2001-November 2003.  Celentano, D.D., Valleroy, L.A., Sifakis, F., Mackellar, D.A., Hylton, J., Thiede, H., McFarland, W., Shehan, D.A., Stoyanoff, S.R., Lalota, M., Koblin, B.A., Katz, M.H., Torian, L.V. (2006). Association between substance use and sexual risk among very young men who have sex with men. Sexually Transmitted Diseases, 33(4), 265-271  Chen, B., Zhu, Y., Guo, R., Ding, S., Zhang, Z., Huaying, C., Zhu, H., Wen, Y. (2016). HIV/AIDS-related knowledge awareness and risk behavior among injection drug users in Maanshan, China: a cross-sectional study  Cheng, T., Johnston, C., Kerr, T., Nguyen, P., Wood, E., DeBeck, K. (2016). Substance use patterns and unprotected sex among street-involved youth in a Canadian setting: a prospective cohort study. BMC Public Health, 16(4)  Chiasson, M., Hirshfield, S., Remien, R.H., Humberstone, M., Wong, T., Wolitski, R.J. (2007). A comparison of on-line and off-line sexual risk in men who have sex with men. J Acquir Immune Defic Syndr. 44, 235-243  Choi, K., Operario, D., Gregorich, S.E., McFarland, W., MacKellar, D., Valleroy, L. (2005). Substance use, substance choice, and unprotected anal intercourse among young Asian American and Pacific Islander men who have sex with men. AIDS Educ Prev., 17(5), 418-429.  Colfax, G.N., Mansergh, G., Guzman, R., Vittinghoff, E., Marks, G., Rader, M., Buchbinder, S. (2001). Drug use and sexual risk behavior among gay and bisexual men who attend circuit parties: A venue-based comparison. JAIDS, 28, 373-379.  Colfax, G., Vittinghoff, E., Husnik, M.J., McKirnan, D., Buchbinder, S., Koblin, B., Celum, C., Chesney, M., Huang, Y., Mayer, K., Bozeman, S., Judson, F.N., Bryant, K.J., Coates, T.J. (2004) Substance use and sexual risk: A participant- and episode-level analysis among a cohort of men who have sex with men. Am J Epidemiol, 159(10)  Cotton-Oldenburg, N.U., Jordan, B.K., Martin, S.L., Kupper, L. (1999). Women inmates' risk sex and drug behavior: Are they related. Am J Drug Alcohol Abuse, 25(1), 129-149.  Couture, M., Evans, J.L., Sothy, N., Stein, E.S., Sichan, K., Maher, L., Page, K. (2012). Correlates of amphetamine-type stimulants use and associations with HIV-related risks among young women engaged in sex work in Phnom Penh, Cambodia. DAD, 120(1-3), 119-126  Drumright, L.N., Little, S.J., Strathdee, S.A., Slymen, D.J., Araneta, M,G.,Malcarne, V.L., Daar, E.S., Gorbach, P.M. (2006). Unprotected anal intercourse and substance use among men who have sex with men with recent HIV infection. J Acquir Immune Defic Syndr., 43, 344-350.  Ferreira, L.O.C., de Oliveira, E.S., Raymond, H.F., Chen, S.Y., McFarland, W. (2008). Use of time-location sampling for systematic behavioral surveillance of truck drivers in Brazil. AIDS Behav., 12, S32-S38  Fullilove, M.T., Golden, E., Fullilove, R.E., Lennon, R., Porterfiled, D., Schwarcz, S., Bolan, G. (1993) Crack cocaine use and high-risk behaviors among sexually active Black adolescents. J Adolesc Health, 14, 295-300  Grov, C., Parsons, J.T., Bimbi, D.S. (2008). In the shadows of a prevention campaign: Sexual risk behavior in the absence of crystal methamphetamine  Halkitis, P.N., Green, K.A., Remien, R.H., Stirratt, M.J., Hoff, C.C., Wolitski, R.J., Parsons, J.T. (2005). Seroconcordant sexual partnerings of HIV-seropositive men who have sex with men. AIDS, 19, S77-S86  Hatfield, L.A., Horvath, K.J., Jacoby, S.M., Simon Rosser, B.R. (2009). Comparison of substance use and risky sexual behavior among a diverse sample of urban, HIV-positive men who have sex with men. J Addict Dis., 28(30), 208-218  Kral, A.H., Lorvick, J., Martinez, A., Lewis, M.A., Orr, A., Anderson, R., Flynn, N., Bluthenthal, R.N. (2011). HIV prevalence and risk among heterosexual methamphetamine injectors in California. Substa Use Misuse, 46(9)  Lackowsky, N.J., Tanner, Z., Sereda, P., Rich, A., Jollimore, J., Montaner, J.S.G., Hogg, R.S., Moore, D.M., Roth, E.A. (2016). An event-level analysis of condom use during anal intercourse among self-reported HIV-negative gay and bisexual men in a treatment as prevention environment. Sex Transm Dis., 43(12), 765-770.  Lambert, G., Cox, J., Hottes, T.S., Tremblay, C., Frigault, L.R., Alary, M., Otis, J., Remis, R.S. (2011). Correlates of unprotected anal sex at last sexual episode: Analysis from surveillance study of men who have sex with men in Montreal  Landovitz, R.J., Tran, T.T.T., Cohn, S.E., Ofotokun, I., Godfrey, C., Kuritzkes, D.R., Lennox, J.L., Currier, J.S., Ribaudo, H.J. (2016). HIV transmission and risk behavior in a cohort of HIV-infected treatment-naïve men and women in the United States. AIDS Behav., 20(12), 2983-2995  Liao, M., Jiang, Z., Zhang, X., Kang, D., Bi, Z., Liu, X., Fu, J., Zhang, N., Mao, W., Jiand, B., Jia, Y. (2011). Syphilis and methamphetamine use among female sex workers in Shandong, Province, China. Sexually Transmitted Disease, 38(1), 57-62  Lowry, R., Hotzman, D., Truman, B.I., Kann, L., Collins, J.L., Kolbe, L.J. (1994). Substance use and HIV-related sexual behaviors among US high school students: Are they related? Am J Public Health, 84(7), 1116-1120  Mansergh, G., Shouse, R.L., Marks, G., Guzman, R., Rader, M., Buchbinder, S., Colfax, G.N. (2006). Methamphetamine and sidenagil (Viagra) use are linked to unprotected receptive and insertive anal sex, respectively in a sample of men who have sex with men. Sex Tram Infect. (82), 131-134  Marshall, B.D.L., Wood, E., Shoveller, J.A., Patterson, T.L., Montaner, J.S.G., Kerr, T. (2011). Pathways to HIV risk and vulnerability among lesbian, gay, and bisexual, and transgendered methamphetamine users: A multi-cohort gender-based analysis. BMC Public Health, 11(20)  Mattison, A.M., Ross, M.W., Wolfson, T., Franklin, D. (2001). Circuit party attendance, club drug use, and unsafe sex in gay men. J Subst Abuse, 13, 119-126  Mburu, G., Tuot, S., Mun, P., Chhoun, P., Chann, N., Yi, S. (2019). Prevalence and correlates of amphetamine-type stimulant use among transgender women in Cambodia. Int J Drug Policy, 74, 136-143  McKetin, R., Lubman, D.I., Baker, A., Dawe, S., Ross, J., Mattick, R.P., Degenhardt, L. (2018). The relationship between methamphetamine use and heterosexual behavior: Evidence from a prospective longitudinal study. Addiction, 113, 1276-1285  McNall, M., Remafedi, G. (1999). Relationship of amphetamine and other substance use to unprotected intercourse among young men who have sex with men. Arch Pediatr Adolesc Med., 153, 1130-1135  Meade, C.S., Watt, M.H., Sikkema, K.J., Deng, L.X., Ranby, K.W., Skinner, D., Pieterse, D., Kalichmann, S.C. (2012). Methamphetamine use is associated with childhood sexual abuse and HIV sexual risk behaviors among patrons of alcohol-serving venues in Cape Town, South Africa. DAD, 126(1-2), 232-239  Melendez-Torres, G.J., Hickson, F., Reid, D., Weatherburn, P., Bonell, C. (2015) Nested event-level case-control study of drug use and sexual outcomes in multipartner encounters reported by men who have sex with men. AIDS Behav., 20, 646-654.  Metsch, L.R., Pereyra, M., Messinger, S., del Rio, C., Strathdee, S.A., Anderson-Mahoney, P., Rudy, E., Marks, G., Gardner, L. (2008) HIV transmission risk behaviors among HIV-infected persons who are successfully linked to care. Clin Infect Dis., 47(4), 577-584  Mimiaga, M.J., Reisner, S.L., Fontaine, Y., Bland, S.E., Driscoll, M.A., Isenberg, D., Cranston, K., Skeer, M.R., Mayer, K.H. (2010). Walking the line: Stimulant use during sex and HIV risk behavior among Black urban MSM. DAD, 110(0), 30-37  Mimiaga, M.J., Reisner, S.L., Grasso, C., Crane, H.M., Safren, S.A., Kitahata, M.M., Schumacher, J.E., Matthews, C., Mayer, K.H. (2013) Substance use among HIV-infected patients engaged in primary care in the United States: Findings from the Center for AIDS Research Network of integrated clinical systems cohort. Am J Public Health, 103(8), 1457-1467  Molitor, F., Traux, S.R., Ruiz, J.D., Sun, R.K. (1998) Association of methamphetamine use during sex with risky sexual behaviors and HIV infection among non-injection drug users. West J Med, 168(2), 93-97  Molitor, F., Ruiz, J.D., Flynn, N., Mikanda, J.N., Sun, R.K., Anderson, R. (1999) Methamphetamine use and sexual and injection risk behaviors among out-of-treatment injection drug users. Am J Drug Alcohol Abuse, 25(3), 475-493.  Munox, F.A., Rollini, R.A., Zuniga, M.L., Strathdee, S.A., Lozada, R., Martinez, G.A., Valles-Medine, A.M., Sirotin, N., Patterson, T.L. (2010) Condom access: Associations with consistent condom use among female sex workers in two northern border cities in Mexico. AIDS Educ Prev., 22(5), 455-465  Narvaez, J.C.M., Jansen, K., Pinheiro, R.T., Kapczinski, F., Silva, R.A., Pechansky, F., Magalhaes, P.V.S. (2014) Violent and sexual behaviors and lifetime use of crack cocaine: A population-based study in Brazil. Soc Psychiatry Psychiatr Epidemiol, 49, 1249-1255  Nerlander, L.M.C., Hoots, B.E., Bradley, H., Brox, D., Thorson, A., Paz-Bailey, G. (2018). HIV infection among MSM who inject methamphetamine in 8 US cities. DAD, 190, 216-223  Ober, A., Shoptaw, S., Wang, P., Gorbach, P., Weiss, R.E. (2009) Factors associated with event-level stimulant use during sex in a sample of older, low-income men who have sex with men in Los Angeles. DAD, 102(1-3), 123-129  Oldenburg, C.E., Jain, S., Mayer, K.H., Mimiaga, M.J. (2015) Post-exposure prophylaxis use and recurrent exposure to HIV among men who have sex with men who use crystal methamphetamine. DAD, 0, 75-80  Pantalone, D.W., Huh, D., Nelson, K.M., Pearson, C.R., Simoni, J.M. (2014) Prospective predictors of unprotected anal intercourse among HIV-seropositive men who have sex with men initiating antiretroviral therapy. AIDS Behav., 18(1)  Pines, H.A., Gorbach, P.M., Weiss, R.E., Reback, C.J., Landovitz, R.J., Mutchler, M.G., Mitsuyasu. (2016) Individual-level, partnership-level, and sexual-event level predictors of condom use during receptive anal intercourse among HIV-negative men who have sex with men in Los Angeles. AIDS Behav, 20(6), 1315-1326  Pylli, M., Middleton, N., Charalambous, A., Raftopoulos, V. (2014) HIV prevalence, sexual and HIV testing behaviors among men who have sex with men in the Republic of Cyprus: 2011-2012 data from a cross-sectional study  Rawstorne, P., Digiusto, E., Worth, H., Zablotska, I. (2007) Associations between crystal methamphetamine use and potentially unsafe sexual activity among gay men in Australia. Arch Sex Behav, 36, 646-654  Rhodes, S.D., Hergenrather, K.C., Yee, L.J., Knipper, E., Wilkin, A.M., Omli, M.R. (2007) Characteristics of a sample of men who have sex with men, recruited from gay bars and internet chat rooms, who report methamphetamine use. AIDS Patient Care STD, 21, 575-583  Santos, G., Coffin, P., Das, M., Matheson, T., DeMicco, E., Raiford, J.L., Vittinghoff, E., Dilley, J.W., Colfax, G., Herbst, J.H. (2013) Dose-relationship associations between number and frequency of substance use and high-risk sexual behaviors among HIV-negative substance-using men who have sex with men (SUMSM) in San Francisco. J Acquir Immune Defic Syndr. 63(4), 540-544.  Schwartz, S., Scheer, S., McFarland, W., Katz, M., Valleroy, L., Chen, S., Catania, J. (2007). Prevalence of HIV infection and predictors od high-transmission sexual risk behaviors among men who have sex with men. Am J Public Health, 97, 1067-1075  Sevelius, J.M., Reznick, O.G., Hart, S.L., Schwarcz, S. (2009) Informing interventions: The importance of contextual factors in the prediction of sexual risk behaviors among transgender women. AIDS Educ Prev., 21(2), 113-127.  Shrier, L.A., Emans, J., Woods, E.R., DuRant, R.H. (1996) The association of sexual risk behaviors and problem drug behaviors in high school students. J Adolesc Health, 20, 377-383  Springer , A.E., Peters, R.J., Shegog, R., White, D.L., Kelder, S.H. (2007) Methamphetamine use and sexual risk behaviors in the U.S. high school students: Findings from a national risk behavior survey. Prev Sci, 8, 103-113  Stahlman, S., Javanbakht, M., Stirland, A., Guerry, S., Gorbach, P.M. (2013). Methamphetamine use among women attending sexually transmitted disease clinics in Los Angeles county. Sex Tram Dis., 40(8), 632-638  Steinberg, J.K., Grella, C.E., Boudov, M.R., Kerndt, P.R., Kadrnka, C.M. (2011). Methamphetamine use and high-risk sexual behaviors among incarcerated female adolescents diagnosed with STD. J Urban Health, 88(2), 352-364  Stioicescu, C., Ameilia, R., Irwanto, Praptoraharjo, I., Mahanani, M. (2019). Syndemic and synergistic effects of intimate partner violence, crystal methamphetamine, and depression or HIV sexual risk behaviors among women who inject drugs in Indonesia. J Urban Health, 97, 477-496  Szwarcwald, C.L., Barbosa-Junior, A., Pascom, A.R., de Souza-Junior, P.R. (2005) Knowledge, practices and behaviours related to HIV transmission among Brazilian populations in the 15-54 years age group, 2004. AIDS, 19 (suppl 4), S51-S58  Tobin, K.E., German, D., Spikes, P., Patterson, J., Latkin, C. (2011) A Comparison of the social and sexual networks of crack-using and non-crack using African American men who have sex with men. J Urban Health, 88(60, 1052-  Tross, S., Hanner, J., Hu, M., Pavlicova, M., Campbell, A., Nunes. E.V. (2009) Substance use and high risk sexual behaviors among women in psychosocial outpatient and methadone maintenance treatment programs. Am J Drug Alcohol Abuse, 35(5), 368-374  Tross, S., Feaster, D.J., Thornes, G., Duan, R., Gomex, Z., Pavlicova, M., Hu, M., Kyle, T., Erickson, S., Spector, A., Haynes, L., Metsch, L.R. (2015) Substance use, depression and sociodemographic determinants of HIV sexual risk behavior in outpatient substance abuse treatment patients. J Addict Med, 96(6), 457-463  Turner, C.M., Santos, G., Arayasirikul, S., Wilson, E.C. (2017) Psychosocial predictors of engagement in sexual risk behavior among trans*female youth ages 16-24 years in San Francisco. J Aquir Immune Defic. Syndr., 74(3), 258-264.  Wechsberg, W.M., Jones, H.E., Zule, W.A., Myers, B.J., Browne, F.A., Kaufman, M.R., Luseno, W., Flisher, A.J., Parry, C.D.H. (2010) Methamphetamine ("tik") use and its association with condom use among out-of-school females in Cape Town, South Africa. Am J Drug Alcohol Abuse, 36(4), 208-213  Woody, G.E., Donnell, D., Seage, G.R., Metzger, D., Marmor, M., Koblin, B.A., Buchbinder, S., Gross, M., Stone, B., Judson, F.N. (1999) Non-injection substance use correlates with risky sex among men having sex with men: Data from HIVNET. DAD, 53, 197-205  Woolf-King, S.E., Rice, T.M., Truong, H.M., Woods, W.J., Jermone, R.C., Carrico, A.W. (2013) Substance use and HIV risk behavior among men who have sex with men: The role of sexual compulsivity. J Urban Health, 90(5), 948-952  Word, C.O., Bowser, B. (1997) Background to crack cocaine addiction and HIV high-risk behavior: The next epidemic. Am J Drug Alcohol Abuse, 23(1), 67-77  Yan, A.F., Chiu, Y., Stoesen, C.A., Wang, M.Q. (2007) STD-/HIV-related sexual risk behaviors and substance use among U.S. rural adolescents. J Natl Med Assoc, 99(12), 1386-1394  Zule, W.A., Costenbader, E.C., Mayers, W.J., Wchsberg, W.M. (2007) Methamphetamine use and risky sexual behavior during heterosexual encounters. Sex Trans Dis, 34(9), 689-694  **References for depression and unprotected sex**  Brickman C, Propert KJ, Voytek C, Metzger D, Gross R. Association Between Depression and Condom Use Differs by Sexual Behavior Group in Patients with HIV. *AIDS Behav*. 2017;21(6):1676-1683. doi:10.1007/s10461-016-1610-8  Brown A, Yung A, Cosgrave E, Killackey E, Buckby J, Stanford C, Godfrey K, McGorry P. Depressed mood as a risk factor for unprotected sex in young people. Australas Psychiatry. 2006 Sep;14(3):310-2. doi: 10.1080/j.1440-1665.2006.02291.x. PMID: 16923045.  Card KG, Lachowsky NJ, Armstrong HL, et al. The additive effects of depressive symptoms and polysubstance use on HIV risk among gay, bisexual, and other men who have sex with men. *Addict Behav*. 2018;82:158-165. doi:10.1016/j.addbeh.2018.03.005  Carney B, White J, Xu X, Sunil T, Daniels C, Byrne M, Ganesan A, Deiss R, Macalino G, Agan BK, Okulicz JF. Relationship between depression and risk behaviors in a US Military population with HIV infection. AIDS Care. 2019 Sep;31(9):1152-1156. doi: 10.1080/09540121.2019.1595522. Epub 2019 Mar 27. PMID: 30917666.  Coyle RM, Lampe FC, Miltz AR, et al. Associations of depression and anxiety symptoms with sexual behaviour in women and heterosexual men attending sexual health clinics: a cross-sectional study. *Sex Transm Infect*. 2019;95(4):254-261. doi:10.1136/sextrans-2018-053689  Kim H, He Y, Pham R, et al. Analyzing the Association Between Depression and High-Risk Sexual Behavior Among Adult Latina Immigrant Farm Workers in Miami-Dade County. *Int J Environ Res Public Health*. 2019;16(7):1120. Published 2019 Mar 28. doi:10.3390/ijerph16071120  Mazzaferro KE, Murray PJ, Ness RB, Bass DC, Tyus N, Cook RL. Depression, stress, and social support as predictors of high-risk sexual behaviors and STIs in young women. J Adolesc Health. 2006 Oct;39(4):601-3. doi: 10.1016/j.jadohealth.2006.02.004. Epub 2006 Jul 10. PMID: 16982400.  Millar BM, Starks TJ, Grov C, Parsons JT. Sexual Risk-Taking in HIV-Negative Gay and Bisexual Men Increases with Depression: Results from a U.S. National Study. *AIDS Behav*. 2017;21(6):1665-1675. doi:10.1007/s10461-016-1507-6  Mimiaga MJ, Biello KB, Sivasubramanian M, Mayer KH, Anand VR, Safren SA. Psychosocial risk factors for HIV sexual risk among Indian men who have sex with men. *AIDS Care*. 2013;25(9):1109-1113. doi:10.1080/09540121.2012.749340  Seth P, Patel SN, Sales JM, DiClemente RJ, Wingood GM, Rose ES. The impact of depressive symptomatology on risky sexual behavior and sexual communication among African American female adolescents. *Psychol Health Med*. 2011;16(3):346-356. doi:10.1080/13548506.2011.554562  Wim, V.B., Christiana, N. & Marie, L. Syndemic and Other Risk Factors for Unprotected Anal Intercourse Among an Online Sample of Belgian HIV Negative Men Who have Sex with Men. *AIDS Behav* 18, 50–58 (2014). https://doi.org/10.1007/s10461-013-0516-y  Zhan W, Shaboltas AV, Skochilov RV, Kozlov AP, Krasnoselskikh TV, Abdala N. Depressive symptoms and unprotected sex in St. Petersburg, Russia. *J Psychosom Res*. 2012;72(5):371-375. doi:10.1016/j.jpsychores.2012.01.015  **References for anxiety and unprotected sex**  Armstrong, G., Jorm, A. F., Samson, L., Joubert, L., Nuken, A., Singh, S., & Kermode, M. (2013). Association of depression, anxiety, and suicidal ideation with high-risk behaviors among men who inject drugs in Delhi, India. *Journal of acquired immune deficiency syndromes (1999)*, *64*(5), 502–510  Beer, L., Tie, Y., Padilla, M., Shouse, R. L., & Medical Monitoring Project (2019). Generalized anxiety disorder symptoms among persons with diagnosed HIV in the United States. *AIDS (London, England)*, *33*(11), 1781–1787. https://doi.org/10.1097/QAD.0000000000002286  Bucek, A., Mellins, C. A., Leu, C. S., Dolezal, C., Korich, R., Wiznia, A., & Abrams, E. J. (2020). Psychiatric disorders and young adult milestones in HIV-exposed, uninfected youth. *AIDS care*, *32*(4), 420–428. https://doi.org/10.1080/09540121.2019.1668535  Cardoso, L. D., & Malbergier, A. (2015). Who is not using condoms among HIV-positive patients in treatment in the largest city in Brazil?. *AIDS care*, *27*(5), 629–636. https://doi.org/10.1080/09540121.2014.986047  Cecil, C., McCrory, E. J., Barker, E. D., Guiney, J., & Viding, E. (2018). Characterising youth with callous-unemotional traits and concurrent anxiety: evidence for a high-risk clinical group. *European child & adolescent psychiatry*, *27*(7), 885–898. https://doi.org/10.1007/s00787-017-1086-8  Hill, L. M., Maman, S., Kilonzo, M. N., & Kajula, L. J. (2017). Anxiety and depression strongly associated with sexual risk behaviors among networks of young men in Dar es Salaam, Tanzania. *AIDS care*, *29*(2), 252–258. https://doi.org/10.1080/09540121.2016.1210075  Jain, S., Oldenburg, C. E., Mimiaga, M. J., & Mayer, K. H. (2016). High Levels of Concomitant Behavioral Health Disorders Among Patients Presenting for HIV Non-occupational Post-exposure Prophylaxis at a Boston Community Health Center Between 1997 and 2013. *AIDS and behavior*, *20*(7), 1556–1563. https://doi.org/10.1007/s10461-015-1021-2  Kugbey, N., Ayanore, M. A., Amu, H., Oppong Asante, K., & Adam, A. (2018). International note: Analysis of risk and protective factors for risky sexual behaviours among school-aged adolescents. *Journal of adolescence*, *68*, 66–69. https://doi.org/10.1016/j.adolescence.2018.06.013  Kutner, B. A., Nelson, K. M., Simoni, J. M., Sauceda, J. A., & Wiebe, J. S. (2017). Factors Associated with Sexual Risk of HIV Transmission Among HIV-Positive Latino Men Who have Sex with Men on the U.S.-México Border. *AIDS and behavior*, *21*(3), 923–934. https://doi.org/10.1007/s10461-016-1449-z  Li, D., Li, C., Wang, Z., & Lau, J. T. (2015). Prevalence and associated factors of unprotected anal intercourse with regular male sex partners among HIV negative men who have sex with men in China: a cross-sectional survey. *PloS one*, *10*(3), e0119977. https://doi.org/10.1371  Liu, J., Zhong, X., Lu, Z., Peng, B., Zhang, Y., Liang, H., Dai, J., Zhang, J., & Huang, A. (2020). Anxiety and Depression Associated with Anal Sexual Practices among HIV-Negative Men Who Have Sex with Men in Western China. *International journal of environmental research and public health*, *17*(2), 464. https://doi.org/10.3390/ijerph17020464  Mota, N. P., Cox, B. J., Katz, L. Y., & Sareen, J. (2010). Relationship between mental disorders/suicidality and three sexual behaviors: results from the National Comorbidity Survey Replication. *Archives of sexual behavior*, *39*(3), 724–734. https://doi.org/10.1007/s10508-008-9463-5  Pantalone, D. W., Huh, D., Nelson, K. M., Pearson, C. R., & Simoni, J. M. (2014). Prospective predictors of unprotected anal intercourse among HIV-seropositive men who have sex with men initiating antiretroviral therapy. *AIDS and behavior*, *18*(1), 78–87. https://doi.org/10.1007/s10461-013-0477-1  Parsons, J. T., Lelutiu-Weinberger, C., Botsko, M., & Golub, S. A. (2013). Predictors of day-level sexual risk for young gay and bisexual men. *AIDS and behavior*, *17*(4), 1465–1477. https://doi.org/10.1007/s10461-012-0206-1  Reyes, J. C., Robles, R. R., Colón, H. M., Marrero, C. A., Matos, T. D., Calderón, J. M., & Shepard, E. W. (2007). Severe anxiety symptomatology and HIV risk behavior among Hispanic injection drug users in Puerto Rico. *AIDS and behavior*, *11*(1), 145–150. https://doi.org/10.1007/s10461-006-9090-x  Sun, W., Wu, M., Qu, P., Lu, C., & Wang, L. (2014). Psychological well-being of people living with HIV/AIDS under the new epidemic characteristics in China and the risk factors: a population-based study. *International journal of infectious diseases : IJID : official publication of the International Society for Infectious Diseases*, *28*, 147–152. https://doi.org/10.1016/j.ijid.2014.07.010  Thai, T. T., Jones, M. K., Harris, L. M., & Heard, R. C. (2017). The association between symptoms of mental disorders and health risk behaviours in Vietnamese HIV positive outpatients: a cross-sectional study. *BMC public health*, *17*(1), 250. https://doi.org/10.1186/s12889-017-4162-6  Turner, A. K., Latkin, C., Sonenstein, F., & Tandon, S. D. (2011). Psychiatric disorder symptoms, substance use, and sexual risk behavior among African-American out of school youth. *Drug and alcohol dependence*, *115*(1-2), 67–73. https://doi.org/10.1016/j.drugalcdep.2010.10.012  Wang, Y., Wang, Z., Jia, M., Liang, A., Yuan, D., Sun, Z., Gan, F., Wang, Y., Cai, Y., & Zhang, Z. (2017). Association between a syndemic of psychosocial problems and unprotected anal intercourse among men who have sex with men in Shanghai, China. *BMC infectious diseases*, *17*(1), 46. https://doi.org/10.1186/s12879-016-2132-8  **References for pain and unprotected sex**  Geibel, S., Luchters, S., King'Ola, N., Esu-Williams, E., Rinyiru, A., & Tun, W. (2008). Factors associated with self-reported unprotected anal sex among male sex workers in Mombasa, Kenya. *Sexually transmitted diseases*, *35*(8), 746–752. https://doi.org/10.1097/OLQ.0b013e318170589d  Gibbs, A., Hatcher, A., Jewkes, R., Sikweyiya, Y., Washington, L., Dunkle, K., Magni, S., Peacock, D., Khumalo, M., Christofides, N. (2019) HIV-Risk behaviors among young men living in informal settlements in South Africa: A cross-sectional analysis and structure equation model. J Acquir Immune Defic Syndr, 81(2), 193-201.  E., Auvert, B., Cherge, J., Sukwa, T., Glynn, J.R., Weiss, H.A., Akam, E., Laourou, M., Carael, M., Buve, A. (2001). Condom use and its association with HIV/sexually transmitted diseases in four urban communities of sub-Saharan Africa, AIDS, 15(suppl 4), S71-S78.  Tsui, J.I., Cheng, D.M., Coleman, S.M., Blokhina, E., Gnatienko, N., Bryant, K., Krupitsky,E., Zvartau, E., Samet, J.H. (2017). Pain and risk behavior among HIV-infected persons in St. Petersburg, Russia. AIDS Behav., 21(6), 1775-1781  **References for alcohol and transactional sex**  Bello B, Moultrie H, Somji A, Chersich MF, Watts C, Delany-Moretlwe S. Alcohol use and sexual risk behaviour among men and women in inner-city Johannesburg, South Africa. *BMC Public Health*. 2017;17(Suppl 3):548. Published 2017 Jul 4. doi:10.1186/s12889-017-4350-4  Lin D, Li X, Yang H, Fang X, Stanton B, Chen X, Abbey A, Liu H. Alcohol intoxication and sexual risk behaviors among rural-to-urban migrants in China. Drug Alcohol Depend. 2005 Jul;79(1):103-12. doi: 10.1016/j.drugalcdep.2005.01.003. Epub 2005 Feb 12. PMID: 15943949; PMCID: PMC1965498.  Liu Y, Ruan Y, Strauss SM, Yin L, Liu H, Amico KR, Zhang C, Shao Y, Qian HZ, Vermund SH. Alcohol misuse, risky sexual behaviors, and HIV or syphilis infections among Chinese men who have sex with men. Drug Alcohol Depend. 2016 Nov 1;168:239-246. doi: 10.1016/j.drugalcdep.2016.09.020. Epub 2016 Sep 30. PMID: 27723554; PMCID: PMC5523945.  Magni S, Christofides N, Johnson S, Weiner R. Alcohol Use and Transactional Sex among Women in South Africa: Results from a Nationally Representative Survey. *PLoS One*. 2015;10(12):e0145326. Published 2015 Dec 18. doi:10.1371/journal.pone.0145326  Raj A, Reed E, Santana MC, Walley AY, Welles SL, Horsburgh CR, Flores SA, Silverman JG. The associations of binge alcohol use with HIV/STI risk and diagnosis among heterosexual African American men. Drug Alcohol Depend. 2009 Apr 1;101(1-2):101-6. doi: 10.1016/j.drugalcdep.2008.11.008. Epub 2008 Dec 30. PMID: 19117698.  Tran BR, Davis A, Ito SI, Matchere F, Reader E, Nkhoma V, Grillo M, Banda AC. Alcohol and Cannabis Use and Sexual Risk Behaviors in the Malawi Defence Force. AIDS Behav. 2018 Sep;22(9):2851-2860. doi: 10.1007/s10461-018-2167-5. PMID: 29869734.  Tran BR, Davis A, Sloan M, Macera C, Mbuyi AM, Kabanda GK. Alcohol use and sexual risk behaviors in the Armed Forces of the Democratic Republic of the Congo. *BMC Public Health*. 2019;19(1):1394. Published 2019 Oct 28. doi:10.1186/s12889-019-7794-x  Weiser SD, Leiter K, Heisler M, McFarland W, Percy-de Korte F, DeMonner SM, Tlou S, Phaladze N, Iacopino V, Bangsberg DR. A population-based study on alcohol and high-risk sexual behaviors in Botswana. PLoS Med. 2006 Oct;3(10):e392. doi: 10.1371/journal.pmed.0030392. PMID: 17032060; PMCID: PMC1592342.  **References for tobacco and transactional sex**  Berg, C. J., Nehl, E. J., Wong, F. Y., He, N., Huang, Z. J., Ahluwalia, J. S., & Zheng, T. (2011). Prevalence and correlates of tobacco use among a sample of MSM in Shanghai, China. *Nicotine & tobacco research: official journal of the Society for Research on Nicotine and Tobacco*, *13*(1), 22–28. <https://doi.org/10.1093/ntr/ntq193>  Buttmann, N., Nielsen, A., Munk, C., Liaw, K. L., & Kjaer, S. K. (2011). Sexual risk taking behaviour: prevalence and associated factors. A population-based study of 22,000 Danish men. *BMC public health*, *11*, 764. <https://doi.org/10.1186/1471-2458-11-764>  Cameron, P., Landess, T., & Cameron, K. (2005). Homosexual sex as harmful as drug abuse, prostitution, or smoking. *Psychological reports*, *96*(3 Pt 2), 915–961. <https://doi.org/10.2466/pr0.96.3c.915-961>  Cohan, D. L., Kim, A., Ruiz, J., Morrow, S., Reardon, J., Lynch, M., Klausner, J. D., Molitor, F., Allen, B., Green Ajufo, B., Ferrero, D., Bell Sanford, G., Page-Shafer, K., Delgado, V., McFarland, W., & Young Women's Survey Team (2005). Health indicators among low income women who report a history of sex work: the population based Northern California Young Women's Survey. *Sexually transmitted infections*, *81*(5), 428–433. <https://doi.org/10.1136/sti.2004.013482>  Lam, T. H., Stewart, S. M., & Ho, L. M. (2001). Smoking and high-risk sexual behavior among young adults in Hong Kong. *Journal of behavioral medicine*, *24*(5), 503–518. <https://doi.org/10.1023/a:1012227728232>  Oppong Asante, K., Meyer-Weitz, A., & Petersen, I. (2014). Substance use and risky sexual behaviours among street connected children and youth in Accra, Ghana. *Substance abuse treatment, prevention, and policy*, *9*, 45. <https://doi.org/10.1186/1747-597X-9-45>  Parrish, D. E., von Sternberg, K., Velasquez, M. M., Cochran, J., Sampson, M., & Mullen, P. D. (2012). Characteristics and factors associated with the risk of a nicotine exposed pregnancy: expanding the CHOICES preconception counseling model to tobacco. *Maternal and child health journal*, *16*(6), 1224–1231. <https://doi.org/10.1007/s10995-011-0848-z>  Pedersen, P. V., Arnfred, A., Algren, M. H., & Juel, K. (2016). Comparison of health behaviors among women brothel workers to those of the general population of women in Denmark. *Women & health*, *56*(4), 376–394. <https://doi.org/10.1080/03630242.2015.1101734>  Philpot, C. R., Harcourt, C. L., & Edwards, J. M. (1989). Drug use by prostitutes in Sydney. *British journal of addiction*, *84*(5), 499–505. <https://doi.org/10.1111/j.1360-0443.1989.tb00606.x>  Wan, X., Shin, S. S., Wang, Q., Raymond, H. F., Liu, H., Ding, D., Yang, G., & Novotny, T. E. (2011). Smoking among young rural to urban migrant women in China: a cross-sectional survey. *PloS one*, *6*(8), e23028. <https://doi.org/10.1371/journal.pone.0023028>  **References for opioids and transactional sex**  Cheng T, Small W, Dong H, Nosova E, Hayashi K, DeBeck K. An age-based analysis of nonmedical prescription opioid use among people who use illegal drugs in Vancouver, Canada. *Subst Abuse Treat Prev Policy*., 2018; 13:41.  Clatts MC, Giang LE, Goldsamt LA, Ti H. Male sex work and HIV risk among young heroin users in Hanoi, Vietnam. *Sex Health*., 2007; 4(4): 261-267.  Clingan SE, Fisher DG, Pedersen WC, Reynolds GL, Xandre P. Impulsiveness, and trait displaced aggression among drug using female sex traders. *Addict Behav*., 2016; 60:24-231.  Matusiewicz, A.K., Ilgen, M.A., Bonar, E.E., Price, A., Bohnert, A.S.B. (2016). The relationship between non-medical use of prescription opioids and sex work among adults in residential substance use treatment. *Journal of Substance Abuse Treatment*, 64, 24-28.  Ngor C, Sopheab H, Mam S, Gorbach P, Chhea C. Factors associated with sexual risk behavior among people who use drugs in communities in Cambodia. *Asia Journal of Public Health*, 2019; 31(4), 335-347.  Pedersen W, Hegna K. Children and adolescents who sell sex: a community study. *Soc Sci Med*., 2003; 56:135-147.  Philpot CR, Harcourt CL, Edwards JM. Drug use by prostitutes in Sydney. *Br J Addict.,* 1989; 84; 499-505.  Reuben J, Serio-Chapman C, Welsh C, Matens R, Sherman SG. Correlates of current transactional sex among a sample of female exotic dancers in Baltimore, MD. *J Urban Health*., 2011; 88(2).  Sawanpanyalert P, Moolphate S, Saksoong P, Piyaworawong S. (2002). Sexual risk behaviors of male current and ex-opiate users in Chiang, Rai Thailand. *J Epidemiol*., 2002; 12(5) 345-350.  Schilling R, El-Bassel N, Ivanoff A, Gilbert A, Su S, Safyer S. Sexual risk behavior of incarcerated, drug use women, 1992. *Public Health Reports*, 1994; 109(4), 539-547.  Spittal PM, Bruneau J, Craib KJP, Miller C, Lamothe F, Weber AE, Li K, Tyndall MW, O'Shaughnessy MV, Schechter MT. Surviving the sex trade: A comparison of HIV risk behaviours among street-involved women in two Canadian cities who inject drugs. *AIDS Care*., 2003; 15(2):187-195.  Suoho K, Humtsoe C, Saggurti N, Sabarwal S, Mahapartra B, Kermoda M. Understanding the associate between injecting and sexual risk behaviors of injecting drug users in Manipur and Nagaland, India. *Harm Reduction Journal*, 2012; 9(40).  Weber AE, Boivin J, Blais L, Haley N, Roy E. Predictors of initiation into prostitution among female street youths. *J Urban Health*., 2004; 81(4):584-595.  Weiser SD, Dilworth SE, Neilands TB, Cohen J, Bangsberg DR, Riley ED. Gender-specific correlates of sex trade among homeless and marginally housed individuals in San Francisco. *J Urban Health*., 2006; 83(4)  **References for stimulants transactional sex**  Baseman J, Ross M, Williams M. Sale of sex for drugs and drugs for sex: An economic context of sexual risk behaviors for STDs. Sex Transm Dis., 1999; 26(8): 444-449.  Bellis MA, Hughes K, Calafat A, Juan M, Ramon A, Rodriguez JA, Mendes F, Schnitzer S, Phillips-Howard P. Sexual uses of alcohol and drugs and the associated health risks: A cross-sectional study of young people in nine European cities. BMC Public Health, 2008; 8:155.  Berg RC, Weatherburn P, Marcus U, Schmidt AJ. Links between transactional sex and HIV/STI-risk and substance use among a large sample of European men who have sex with men. BMC Infectious Disease, 2019; 19:686.  Campsmith ML, Nakashima AK, Jones JL. Association between crack cocaine use and high-risk sexual behaviors after HIV diagnosis. JAIDS, 2000; 25:192-198.  Chettiar J, Shannon K, Woods E, Zhang R, Kerr T. Survival sex work involvement among street-involved youth who use drugs in a Canadian setting. J Public Health (Oxf), 2010; 32(3): 322-327.  Clatts MC, Giang LM, Goldsamt LA, Yi H. Male sex work and HIV risk among young heroin users in Hanoi, Vietnam. Sex Health, 2007; 4(4): 261-267.  de Souza CTV, Diaz T, Sutmoller F, Bastos FI. The association of socioeconomic status and use of crack/cocaine with unprotected anal sex in a cohort of men who have sex with men in Rio de Janeiro, Brazil. JAIDS, 2002; 29:95-100.  El-Bassel N, Schilling RF, Irwin KL, Faruque S, Gilbert L, Von Bargen J, Serrano Y, Edlin BR. Sex trading and psychological distress among women recruited from the streets of Harlem. Am J Public Health, 19997; 87(1): 66-70.  El-Bassel N, Simoni JM, Cooper DK, Gilbert L, Schilling RF. Sex trading and psychological distress among women on methadone. Pscyhol Addict Behav, 2001;15(3):177-184.  Fischer B, Rehm J, Patra J, Kalousek K, Haydon E, Tyndall M, El-Guebaly N. Crack across Canada: Comparing crack users and crack non-users in Canadian multi-city cohort of illicit opioid users. Addiction, 2006;101:1760-1770.  Gilchrist G, Singleton N, Donmall M, Jones A. Prevalence and factors associated with sex trading in the year prior to entering treatment for drug misuse in England. Drug and Alcohol Dependence, 2015;152:116-122.  Golder S, Logan TK. Correlates and predictors of women's sex trading over time among a sample of out-of-treatment drug abusers. AIDS Behav, 2007;11:628-640.  Guimarães, R. A., Rodovalho, A. G., Fernandes, I. L., Silva, G. C., de Felipe, R. L., Vera, I., Gregório, V. D., & Lucchese, R. (2016). Transactional Sex among Noninjecting Illicit Drug Users: Implications for HIV Transmission. *TheScientificWorldJournal*, *2016*, 4690628. https://doi.org/10.1155/2016/4690628  Gunn RA, Montes JM, Toomey KE, Rolfs RT, Graanspan JR, Spitters CE, Waterman SH. Syphilis in San Dieago county 1983-1992: Crack cocaine, prostitution, and the limitations of partner notification. Sex Trams Dis., 1994; 22(1):60-66.  Hood JE, Buskin SE, Golden MR, Glick SN, Banta-Green C, Dombrowski JC. The changing burden of HIV attributable to methamphetamine among men who have sex with men in King County, Washington. AIDS Patient Care and STDs, 2018;32(6): 223-233.  Kuo I, Greenberg AE, Magnus M, Phillips G, Rawls A, Peterson J, Hamilton F, West-Ojo T, Hader S. High prevalence of substance use among heterosexuals living in communities with high rates of AIDS and poverty in Washington, DC. Drug and Alcohol Dependence, 2011;117:139-144.  Kuyper LM, Lampinen TM, Spittal PM, Hogg RS, Schechter MT, Wood E. Factors associated with sex trade involvement among male participants in a prospective study of injection drug users. Sex Trams Infect., 2004; 80:531-535.  Latkin CA, Hua W, Forman VL. The relationship between social network characteristics and exchanging sex for drugs or money among drug users in Baltimore, MD, USA. Int J STD AIDS, 2004; 14:700-775.  Lloyd-Smith E, Wood E, Li K, Montaner JSG, Kerr T. Incidence and determinants of initiation into cocaine injection and correlates of frequent cocaine injectors. Drug Alcohol Depend., 2009; 99(1-3):176-182.  Mburu, G., Tuot, S., Mun, P., Chhoun, P., Chann, N., Yi, S. (2019). Prevalence and correlates of amphetamine-type stimulant use among transgender women in Cambodia. Int J Drug Policy, 74, 136-143.  Mehrabadi A, Craib KJP, Patterson K, Adam W, Moniruzzaman A, Ward-Burkitt B, Schechter MT, Pittal PM. The cedar project: A comparison of HIV-related vulnerabilities amongst young Aboriginal women surviving drug use and sex work in two Canadian cities. Int J Drug Policy, 2008; 19:159-168.  Molitor F, Traux SR, Ruiz JD, Sun RK. Association of methamphetamine use during sex with risky sexual behaviors and HIV infection among non-injection drug users. West J Med, 1998; 168(2): 93-97.  Moltor, F., Ruiz, J.D., Flynn, N., Mikanda, J.N., Sun, R.K., Anderson, R. (1999) Methamphetamine use and sexual and injection risk behaviors among out-of-treatment injection drug users. Am J Drug Alcohol Abuse, 25(3), 475-493.  Newman PA, Rhodes F, Weiss RE. Correlates of sex trading among drug-using men who have sex with men. Am J Public Health, 2004; 94(11):1998-2003.  Ober A, Shoptaw S, Wang P, Gorbach P, Weiss RE. Factors associated with event-level stimulant use during sex in a sample of older, low-income men who have sex with men in Los Angeles. Drug and Alcohol Depend., 2009; 102(1-3):123-129.  Philpot CR, Harcourt CL, Edwards JM. Drug use by prostitutes in Sydney. Br J Addict., 1989; 84:499-505.  Reuben J, Serio-Chapman C, Wlesh C, Matens R, Sherman SG. Correlates of current transactional sex among a sample of female exotic dancers in Baltimore, MD. J Urban Health, 2011; 88(2):342-351.  Rolfs RT, Goldberg M, Sharrar RG. Risk factors for syphilis: Cocaine use and prostitution. Am J Public Health, 1990;80(7):853-857.  Rusch ML, Lozada R, Pollini RA, Vera A, Patterson TL, Case P, Strathdee SA. Polydrug use among IDUs in Tijuana, Mexico: Correlates of methamphetamine use and route of administration by gender. J Urban Health, 2009; 85(6):760-775.  Saw YM, Saw TN, Wai KM, Poudel KC, Win HH. Correlates of sex trading among male non-injecting drug users in Myanmar: a cross-sectional study. Harm Reduction Journal, 2016;13:34.  Schilling R, El-Bassel N, Ivanoff A, Gilbert L, Su K, Safyer SM. Sexual risk behavior of incarcerated, drug-using women, 1992. Public Health Report, 1994; 109(4):539-547.  Sevelius, J.M., Reznick, O.G., Hart, S.L., Schwarcz, S. (2009) Informing interventions: The importance of contextual factors in the prediction of sexual risk behaviors among transgender women. AIDS Educ Prev., 21(2), 113-127.  Sherman SG, Reuben J, Chapman CS, Lilleston P. Risks associated with crack cocaine smoking among exotic dancers in Baltimore, MD. Drug and Alcohol Depend., 2011; 114(0):249-252.  Sherman SG, Hast M, Park JU, Decker MR, Flynn C, German D. Correlates of exchange sex among a population-based sample of low-income women who have heterosexual sex in Baltimore. AIDS Care, 2018; 30(10):1273-1281.  Spittal PM, Bruneau J, Craib KJP, Miller C, Lamothe F, Weber AE, Li K, Tyndall MW, O'Shaughnessy MV, Schechter MT. Surviving the sex trade: A comparison of HIV risk behaviors among street-involved women in two Canadian cities who inject drugs. AIDS Care, 2003; 15(2):187-195.  Stahlman, S., Javanbakht, M., Stirland, A., Guerry, S., Gorbach, P.M. (2013). Methamphetamine use among women attending sexually transmitted disease clinics in Los Angeles County. Sex Tram Dis., 40(8), 632-638.  Steinberg, J.K., Grella, C.E., Boudov, M.R., Kerndt, P.R., Kadrnka, C.M. (2011). Methamphetamine use and high-risk sexual behaviors among incarcerated female adolescents diagnosed with STD. J Urban Health, 88(2), 352-364.  Tucker JS, Wenzel SL, Kennedy DP, Golinelli D, Ewing B. Sex trade behavior among heterosexually active homeless men. Arch Sex Behav., 2013; 42(8).  Uhlmann S, DeBeck K, Simo A, Kerr T, Montaner JSG, Wood E. Health and social harms associated with crystal methamphetamine use among street-involved youth in a Canadian setting. Am J Addict., 2014; 23(4):393-393.  Urada LA, Strathdee SA, Morisky DE, Schilling RF, Simbulan NP, Estacio LR, Raj A. Sex work and its associations with alcohol and methamphetamine use among female bar and spa workers in the Phillippines. Asia Pac J Public Health., 2014; 26(2):138-146.  Vivancos R, Maskrey V, Rumball D, Harvey I, Holland R. Crack/cocaine use in rural county of England. J Public Health (Oxf)., 2006; 28(2):96-103.  Vu NTT, Holt M, Phan HTT, Le HT, La LT, Tran GM, Doan TT, Nguyen TNN, de Wit J. Amphetamine-type stimulant use among men who have sex with men (MSM) in Vietnam: Results from a socio-ecological community-based study. Drug and Alchol Depend., 2016; 158:110-117.  Wagner KD, Pitpitan EV, Chavarin CV, Magis-Rodriguez C, Patterson TL. Drug-using male clients of female sex workers who report being paid for sex: HIV/STI, demographic and drug use correlates. Sex Transm Dis., 2013; 40(8).  Walters SM, Rivera AV, Reilly KH, Anderson BJ, Bolden B, Wogayehu A, Neaigus A, Braunstein S. Exchange sex among persons who inject drugs in the New York metropolitan area: The importance of local context, gender, and sexual identity. AIDS Behav., 2018; 22:2773-2787.  Walters SM, Brakmajer A, Coston B, Yoon I, Grov C, Downing MJ, Teran R, Hirschfield S. A syndemic model of exchange sex among HIV-positive men who have sex with men. Arch Sex Behav., 2020; 49(6):1965-1978.  Weber AE, Craib KJP, Chan K, Martindale S, Miller M, Schechter MT, Hogg RS. Sex trade involvement and rates of human immunodeficiency virus positivity among young gay and bisexual men. Int J Epidemiol., 2001; 30:1449-1454.  Weber AE, Boivin J, Blais L, Haley N, Roy E. HIV risk profile and prostitution among female street youth. J Urban Health., 2002; 79(4):525-535.  Weeks MR, Grier M, Romero-Daza N, Puglisi-Vasquez M, Singer M. Streets, drugs, and the economy of sex in the age of AIDS. Women & Health, 1998; 27(1-2):205-229.  Wei C, Guadamuz TE, Lim SH, Huang Y, Koe S. Patterns and levels of illicit drug use among men who have sex with men in Asia. Drug Alcohol Depend., 2012; 120(1-3):246-249.  Weiser SD, Dilworth SE, Neilands TB, Cohen J, Bangsberg DR, Riley ED. Gender-specific correlates of sex trade among homeless and marginally house individuals in San Francisco. J Urban Health., 2006; 83(4): 736-740.  Windle M. The trading of sex for money or drugs, sexually transmitted diseases (STDs), and HIV-related behaviors among multisubtsance using alcoholic inpatients. *Drug Alcohol Depend.*¸1997; 49:33-38.  **References for depression and transactional sex**  Burnette ML, Lucas E, Ilgen M, Frayne SM, Mayo J, Weitlauf JC. Prevalence and health correlates of prostitution among patients entering treatment for substance use disorders. Arch Gen Psychiatry, 2008; 65(3):337-344.  Gilchrist G, Gruer L, Atikinson J. Comparison of drug use and psychiatric morbidity between prostitute and non-prostitute female drug users in Glasgow, Scotland. Addict Behav., 2005; 30:1019-1023.  Hutton HE, Lyketsos CG, Zenilman JM, Thompson RE, Erbelding EJ. Depression and HIV risk behavior among patients in a sexually transmitted disease clinic. Am J Psychiatry., 2004; 161:912-914.  Javanbakht M, Shoptaw S, Ragsdale A, Brookmeyer R, Bolan R, Gorbach PM. Depressive symptoms and substance use: Changes overtime among a cohort of HIV-positive and HIV-negative MSM. Drug and Alcohol Depend., 2020; 207: 107770.  Korhonen C, Kimani M, wahome E, Otieno F, Lorwar RR, Doshi M, Mathenge J, Kimani J, Sander EJ, Graham SM. Depressive symptoms and problematic alcohol and other substance use in 1476 gay, bisexual, and other MSM at three research sites in Kenya. AIDS., 2018; 32:1507-1515.  Larsen A, Kinuthia J, Lagat H, Sila J, Abuna F, Kohler P, John-Stewart G, Pintye J. Depression and HIV risk behaviors among adolescent girls and young women seeking family planning services in Western Kenya. Int J STD AIDS., 2020; 31(7):652-664.  Logie CH, Marcus N, Wang Y, Lacombe-Duncan A, Levermore K, Jones N, Bryan N, Back R, Marshall A. Contextualising sexual health practices among lesbian and bisexual women in Jamaica: a multi-methods study. Reprod Health Matters., 2018; 26(52):109-127.  Logie CH, Wang Y, Lacombe-Duncan A, Jones N, Ahmed U, Levermore K, Neil A, Ellis T, Bryan N, Marshall A, Newman PA. Factors associated with sex work involvement among transgender women in Jamaica: a cross-sectional study. J Int AIDS Soc., 2017; 20:21422.  Nduna M, Jewkes RK, Dunkle KL, Jama Shai NP, Colman I. Associations between depressive symptoms, sexual behaviour and relationship characteristics: a prospective cohort study of young women and men in Eastern Cape, South Africa. J Int AIDS Soc., 2010; 13:44.  Okafor CN, Christodoulou J, Bantjes J, Qondela T, Stewart J, Shoptaw S, Tomlinson M, Rotherman-Borus M. Understanding HIV risk behaviors among young men in South Africa: A syndemic approach. AIDS Behav., 2018; 22(12):3962-3970.  Olaiya O, Nerlander L, Mattson CL, Beer L. Exchange sex among people receiving medical care for HIV in the United States - medical monitoring project 2009-2013. AIDS Care., 2018; 30(10):1315-1321.  Patton RA, Cunningham RM, Blow FC, Zimmerman MA, Booth BM, Walton MA. Transactional sex involvement: Exploring risk and promotive factors among substance-using youth in an urban emergency department. J Stud Alcohol Drugs., 2014; 75:573-579.  Rael CT, Davis A. Elevated depression symptoms and key associated factors in female sex workers and women living with HIV/AIDS in the Dominican Republic. Int J STD AIDS., 2017; 28(5):433-440.  Safren SA, Thomas BE, Mimiaga MJ, Chandrasekaran V, Menon S, Swaminathan S, Mayer KH. Depressive symptoms and human immunodeficiency virus risk behavior among men who have sex with men in Chennai, India. Psychol Health Med., 2009; 14(6):705-715.  Sileo KM, Kershaw TS, Callands TA. A syndemic of psychosocial and mental health problems in Liberia: Examining the link to transactional sex among young pregnant women. Glob Public Health., 2019; 14(10):1442-1453.  Smit J, Myer L, Middelkoop K, Seedat S, Wood R, Bekker LG, Stein DJ. Mental health and sexual risk behaviours in a South African township: A community-based cross-sectional study. Public Health, 2006; 120:534-542.  Stiffman AR, Dore P, Earls F, Cunningham R. The influence of mental health problems on AIDS-related risk behaviors in young adults. J Nerv Ment Dis., 1992; 180(5):314-320.  Stoicescu C, Ameilia R, Irwanto, Praptoraharjo I, Mahanani M. Syndemic and synergistic effects of intimate partner violence, crystal methamphetamine, and depression on HIV sexual risk behaviors among women who inject drugs in Indonesia. J Urban Health., 2019; 96:477-496.  Tucker JS, Hu J, Golinelli D, Kennedy DP, Green HD, Wenzel SL. Social network and individual correlates of sexual risk behavior among homeless MSM youth. J Adolesc Health., 2012; 51(4):386-392.  Walters SM, Braksmajer A, Coston B, Yoon I, Grov C, Downing MJ, Teran R, Hirshfield S. A syndemic model of exchange sex among HIV-positive men who have sex with men. Arch Sex Behav., 2020; 49(6):1965-1978.  Yang H, Li X, Stanton B, Chen X, Liu H, Fang X, Lin D, Mao R. HIV-related risk factors associated with commercial sex among female migrants in China. Health Care Women Int., 2005; 26(2): 134-148.  Yates GL, Mackenzie RG, Pennbridge J, Swofford A. A risk profile comparison of homeless youth involved in prostitution and homeless youth not involved. J Adolesc Health., 1991; 12:545-548.  **References for anxiety and transactional sex**  Bauermeister, J. A., Eaton, L., Meanley, S., Pingel, E. S., & UHIP Partnership (2017). Transactional Sex With Regular and Casual Partners Among Young Men Who Have Sex With Men in the Detroit Metro Area. *American journal of men's health*, *11*(3), 498–507. <https://doi.org/10.1177/1557988315609110>  Burnette, M. L., Lucas, E., Ilgen, M., Frayne, S. M., Mayo, J., & Weitlauf, J. C. (2008). Prevalence and health correlates of prostitution among patients entering treatment for substance use disorders. *Archives of general psychiatry*, *65*(3), 337–344. <https://doi.org/10.1001/archpsyc.65.3.337>  Huang, W., Operario, D., Dong, Y., Zaller, N., Song, D., He, H., Tao, H., Xia, J., & Zhang, H. (2014). HIV-related risk among female migrants working in entertainment venues in China. *Prevention science : the official journal of the Society for Prevention Research*, *15*(3), 329–339. <https://doi.org/10.1007/s11121-013-0423-5>  **References for pain and transactional sex** *(no eligble studies)*  **References for alcohol and multiple sexual partners**  Abdala N, White E, Toussova OV, et al. Comparing sexual risks and patterns of alcohol and drug use between injection drug users (IDUs) and non-IDUs who report sexual partnerships with IDUs in St. Petersburg, Russia. *BMC Public Health*. 2010;10:676. Published 2010 Nov 5. doi:10.1186/1471-2458-10-676  Bello B, Moultrie H, Somji A, Chersich MF, Watts C, Delany-Moretlwe S. Alcohol use and sexual risk behaviour among men and women in inner-city Johannesburg, South Africa. *BMC Public Health*. 2017;17(Suppl 3):548. Published 2017 Jul 4. doi:10.1186/s12889-017-4350-4  Choudhry V, Agardh A, Stafström M, Östergren PO. Patterns of alcohol consumption and risky sexual behavior: a cross-sectional study among Ugandan university students. *BMC Public Health*. 2014;14:128. Published 2014 Feb 6. doi:10.1186/1471-2458-14-128  Ghebremichael M, Paintsil E, Larsen U. Alcohol abuse, sexual risk behaviors, and sexually transmitted infections in women in Moshi urban district, northern Tanzania. *Sex Transm Dis*. 2009;36(2):102-107. doi:10.1097/OLQ.0b013e31818b20e6  Gordon KS, Edelman EJ, Justice AC, et al. Minority Men Who Have Sex with Men Demonstrate Increased Risk for HIV Transmission. *AIDS Behav*. 2017;21(5):1497-1510. doi:10.1007/s10461-016-1590-8  Kalina O, Geckova AM, Jarcuska P, Orosova O, van Dijk JP, Reijneveld SA. Psychological and behavioural factors associated with sexual risk behaviour among Slovak students. *BMC Public Health*. 2009;9:15. Published 2009 Jan 13. doi:10.1186/1471-2458-9-15  Lin D, Li X, Yang H, Fang X, Stanton B, Chen X, Abbey A, Liu H. Alcohol intoxication and sexual risk behaviors among rural-to-urban migrants in China. Drug Alcohol Depend. 2005 Jul;79(1):103-12. doi: 10.1016/j.drugalcdep.2005.01.003. Epub 2005 Feb 12. PMID: 15943949; PMCID: PMC1965498.  Liu Y, Ruan Y, Strauss SM, Yin L, Liu H, Amico KR, Zhang C, Shao Y, Qian HZ, Vermund SH. Alcohol misuse, risky sexual behaviors, and HIV or syphilis infections among Chinese men who have sex with men. Drug Alcohol Depend. 2016 Nov 1;168:239-246. doi: 10.1016/j.drugalcdep.2016.09.020. Epub 2016 Sep 30. PMID: 27723554; PMCID: PMC5523945.  Pham QD, Nguyen TV, Nguyen PD*, et al*. Men who have sex with men in southern Vietnam report high levels of substance use and sexual risk behaviours but underutilise HIV testing services: a cross-sectional study *Sexually Transmitted Infections*2015;91:178-182.  Tran BR, Davis A, Ito SI, Matchere F, Reader E, Nkhoma V, Grillo M, Banda AC. Alcohol and Cannabis Use and Sexual Risk Behaviors in the Malawi Defence Force. AIDS Behav. 2018 Sep;22(9):2851-2860. doi: 10.1007/s10461-018-2167-5. PMID: 29869734.  Tran BR, Davis A, Sloan M, Macera C, Mbuyi AM, Kabanda GK. Alcohol use and sexual risk behaviors in the Armed Forces of the Democratic Republic of the Congo. *BMC Public Health*. 2019;19(1):1394. Published 2019 Oct 28. doi:10.1186/s12889-019-7794-x  Weiser SD, Leiter K, Heisler M, McFarland W, Percy-de Korte F, DeMonner SM, Tlou S, Phaladze N, Iacopino V, Bangsberg DR. A population-based study on alcohol and high-risk sexual behaviors in Botswana. PLoS Med. 2006 Oct;3(10):e392. doi: 10.1371/journal.pmed.0030392. PMID: 17032060; PMCID: PMC1592342.  Wirtz AL, Zelaya CE, Latkin C, et al. Alcohol Use and Associated Sexual and Substance Use Behaviors Among Men Who Have Sex with Men in Moscow, Russia. *AIDS Behav*. 2016;20(3):523-536. doi:10.1007/s10461-015-1066-2  **References for tobacco and multiple sexual partners**  Buttmann, N., Nielsen, A., Munk, C., Liaw, K. L., & Kjaer, S. K. (2011). Sexual risk taking behaviour: prevalence and associated factors. A population-based study of 22,000 Danish men. *BMC public health*, *11*, 764. <https://doi.org/10.1186/1471-2458-11-764>  Cameron, P., Landess, T., & Cameron, K. (2005). Homosexual sex as harmful as drug abuse, prostitution, or smoking. *Psychological reports*, *96*(3 Pt 2), 915–961. <https://doi.org/10.2466/pr0.96.3c.915-961>  Cavazos-Rehg, P. A., Krauss, M. J., Spitznagel, E. L., Schootman, M., Cottler, L. B., & Bierut, L. J. (2011). Number of sexual partners and associations with initiation and intensity of substance use. *AIDS and behavior*, *15*(4), 869–874. <https://doi.org/10.1007/s10461-010-9669-0>  Doku D. (2012). Substance use and risky sexual behaviours among sexually experienced Ghanaian youth. *BMC public health*, *12*(1), 571. <https://doi.org/10.1186/1471-2458-12-571>  Duan, S., Jin, Z., Liu, X., Yang, Y., Ye, R., Tang, R., Gao, M., Ding, Y., & He, N. (2017). Tobacco and alcohol use among drug users receiving methadone maintenance treatment: a cross-sectional study in a rural prefecture of Yunnan Province, Southwest China. *BMJ open*, *7*(3), e014643. <https://doi.org/10.1136/bmjopen-2016-014643>  Edelman, N., Cassell, J. A., de Visser, R., Prah, P., & Mercer, C. H. (2017). Can psychosocial and socio-demographic questions help identify sexual risk among heterosexually-active women of reproductive age? Evidence from Britain's third National Survey of Sexual Attitudes and Lifestyles (Natsal-3). *BMC public health*, *17*(1), 5. <https://doi.org/10.1186/s12889-016-3918-8>  Escobedo, L. G., Reddy, M., & DuRant, R. H. (1997). Relationship between cigarette smoking and health risk and problem behaviors among US adolescents. *Archives of pediatrics & adolescent medicine*, *151*(1), 66–71. <https://doi.org/10.1001/archpedi.1997.02170380070011>  Holly, E. A., Cress, R. D., Ahn, D. K., Aston, D. A., Kristiansen, J. J., & Felton, J. S. (1992). Characteristics of women by smoking status in the San Francisco Bay Area. *Cancer epidemiology, biomarkers & prevention : a publication of the American Association for Cancer Research, cosponsored by the American Society of Preventive Oncology*, *1*(6), 491–497  Howard, D. E., & Wang, M. Q. (2004). Multiple sexual-partner behavior among sexually active US adolescent girls. *American journal of health behavior*, *28*(1), 3–12. <https://doi.org/10.5993/ajhb.28.1.1>  Järvelaid M. (2004). Adolescent tobacco smoking and associated psychosocial health risk factors. *Scandinavian journal of primary health care*, *22*(1), 50–53. <https://doi.org/10.1080/02813430310000988>  Kalina, O., Geckova, A. M., Jarcuska, P., Orosova, O., van Dijk, J. P., & Reijneveld, S. A. (2009). Psychological and behavioural factors associated with sexual risk behaviour among Slovak students. *BMC public health*, *9*, 15. <https://doi.org/10.1186/1471-2458-9-15>  Kvaavik, E., Lund, I., Nygård, M., & Hansen, B. T. (2016). Lifestyle Correlates of Female Snus Use and Smoking: A Large Population-Based Survey of Women in Norway. *Nicotine & tobacco research : official journal of the Society for Research on Nicotine and Tobacco*, *18*(4), 431–436. <https://doi.org/10.1093/ntr/ntv126>  Lam, T. H., Stewart, S. M., & Ho, L. M. (2001). Smoking and high-risk sexual behavior among young adults in Hong Kong. *Journal of behavioral medicine*, *24*(5), 503–518. https://doi.org/10.1023/a:1012227728232  Nischan, P., Ebeling, K., & Schindler, C. (1988). Smoking and invasive cervical cancer risk. Results from a case-control study. *American journal of epidemiology*, *128*(1), 74–77. <https://doi.org/10.1093/oxfordjournals.aje.a114960>  Oppong Asante, K., Meyer-Weitz, A., & Petersen, I. (2014). Substance use and risky sexual behaviours among street connected children and youth in Accra, Ghana. *Substance abuse treatment, prevention, and policy*, *9*, 45. <https://doi.org/10.1186/1747-597X-9-45>  Pahl, K., Brook, D. W., Morojele, N. K., & Brook, J. S. (2010). Nicotine dependence and problem behaviors among urban South African adolescents. *Journal of behavioral medicine*, *33*(2), 101–109. <https://doi.org/10.1007/s10865-009-9242-3>  Parazzini, F., Cavalieri d'Oro, L., Negri, E., & La Vecchia, C. (1992). Determinants of sexual habits in Italian females. *Genitourinary medicine*, *68*(6), 394–398. <https://doi.org/10.1136/sti.68.6.394>  Parrish, D. E., von Sternberg, K., Velasquez, M. M., Cochran, J., Sampson, M., & Mullen, P. D. (2012). Characteristics and factors associated with the risk of a nicotine exposed pregnancy: expanding the CHOICES preconception counseling model to tobacco. *Maternal and child health journal*, *16*(6), 1224–1231. <https://doi.org/10.1007/s10995-011-0848-z>  Peltzer, K., & Pengpid, S. (2016). Risk and Protective Factors Affecting Sexual Risk Behavior Among School-Aged Adolescents in Fiji, Kiribati, Samoa, and Vanuatu. *Asia-Pacific journal of public health*, *28*(5), 404–415. https://doi.org/10.1177/1010539516650725  Richter, D. L., Valois, R. F., McKeown, R. E., & Vincent, M. L. (1993). Correlates of condom use and number of sexual partners among high school adolescents. *The Journal of school health*, *63*(2), 91–96. <https://doi.org/10.1111/j.1746-1561.1993.tb06087.x>  Saha, A., Chaudhury, A. N., Bhowmik, P., & Chatterjee, R. (2010). Awareness of cervical cancer among female students of premier colleges in Kolkata, India. *Asian Pacific journal of cancer prevention : APJCP*, *11*(4), 1085–1090  Sivasithamparam, J., Visk, C. A., Cohen, E. E., & King, A. C. (2013). Modifiable risk behaviors in patients with head and neck cancer. *Cancer*, *119*(13), 2419–2426. <https://doi.org/10.1002/cncr.27993>  Valois, R. F., Oeltmann, J. E., Waller, J., & Hussey, J. R. (1999). Relationship between number of sexual intercourse partners and selected health risk behaviors among public high school adolescents. *The Journal of adolescent health : official publication of the Society for Adolescent Medicine*, *25*(5), 328–335. <https://doi.org/10.1016/s1054-139x(99)00051-8>  Yan, A. F., Chiu, Y. W., Stoesen, C. A., & Wang, M. Q. (2007). STD-/HIV-related sexual risk behaviors and substance use among U.S. rural adolescents. *Journal of the National Medical Association*, *99*(12), 1386–1394  **References for opioids and multiple sexual partners**  Castilla J, Barrio G, Belza MJ, de la FuentaL. Drug and alcohol consumption and sexual risk behavior among young adults: results from a national survey. Drug and Alcohol Dep., 1999; 56, 47-53.  Luo X, Zhao P, Gong X, Zhang L, Tang W, Zou X, Chen W, Ling L. Concurrent heroin use and correlates among methadone maintenance treatment clients: a 12-month follow-up study in Guangdong province, China. Int J Environ Res Public Health., 2016; 13(305).  Quian H, Hao C, Ruan Y, Cassell HM, Chen K, Qin G, Yin L, Schumacher JE, Liang S, Shao Y. Impact of methadone on drug use and risky sex in China. J Subst Abuse Treat., 2008; 34:391-397.  Rotily M, Galinier-Pujol A, Vernay-Vaisse C. Risk behaviours of inmates in south-eastern France. AIDS Care., 1994; 7(1).  Shava E, Lipira LE, Beauchamp GG, Donnell DJ, Lockman S, Ruan Y, Shao Y. Risky sexual behavior among individuals receiving buprenorphine/naloxone opiate dependency treatment: HIV prevention trial network (HPTN) 058. J Acquir Immune Defic Syndr., 2018; 78(3):300-307.  Suoho K, Humtsoe C, Saggurti N, Sabarwal S, Mahapartra B, Kermode M. Understanding the associate between injecting and sexual risk behaviors of injecting drug users in Manipur and Nagaland, India. Harm Reduction Journal, 2021; 9(40).  **References for stimulants and multiple sexual partners**  Bellis MA, Hughes K, Calafat A, Juan M, Ramon A, Rodriguez JA, Mendes F, Schnitzer S, Phillips-Howard P. Sexual uses of alcohol and drugs and the associated health risks: A cross-sectional study of young people in nine European cities. *BMC Public Health*, 2008; 8:155.  Borders TF, Stewart KE, Wright PB, Leukefeld C, Falck RS, Carlson RG, Booth BM. Risky sex in rural America: Longitudinal changes in a community-based cohort of methamphetamine and cocaine users. *Am J Addict.*, 2013; 22(6):535-542.  Campsmith ML, Nakashima AK, Jones JL. Association between crack cocaine use and high-risk sexual behaviors after HIV diagnosis. *JAIDS*, 2000; 25:192-198.  Castilla J, Barrio G, Belza MJ, de la Fuenta L. Drug and alcohol consumption and sexual risk behaviour among young adults: Results from a national survey. *Drug and Alcohol Dependence*, 1999; 56:47-53.  Couture M, Evans JL, Sothy N, Stein ES, Sichan K, Maher L, Page K. Correlates of amphetamine-type stimulants use and associations with HIV-related risks among young women engaged in sex work in Phnom Penh, Cambodia. *DAD*, 2012; 120(1-3): 119-126  Flom PL, Friedman SR, Kottiri BJ, Neaigus A, Curtis R, Des Jarlais DC, Sandoval M, Zenilam JM. Stigmatized drug use, sexual partner concurrency, and other sex risk network and behavior characteristics of 18- to 24-year-old youth in a high risk neighborhood. *Sex Trams Dis.*, 2001; 28(10):598-607.  Greenberg J, Schnell D, Conlon R. Behaviors of crack cocaine users and their impact on early syphilis intervention. *Sex Trams Dis.*, 1992; 19(6):346-350.  Gunn RA, Montes JM, Toomey KE, Rolfs RT, Granspan JR, Spitters CE, Waterman SH. Syphilis in San Diego county 1983-1992: Crack cocaine, prostitution, and the limitations of partner notification. *Sex Trams Dis*., 1994; 22(1):60-66.  Kuo I, Greenberg AE, Magnus M, Phillips G, Rawls A, Peterson J, Hamilton F, West-Ojo T, Hader S. High prevalence of substance use among heterosexuals living in communities with high rates of AIDS and poverty in Washington, DC. *Drug and Alcohol Dependence*, 2011; 117:139-144.  Lorvick J, Martinez A, Gee L, Kral AH. Sexual and injection risk among women who inject methamphetamine in San Francisco. *J Urban Health*, 2006; 83(3): 497-505.  McKetin R, Lubman DI, Baker A, Dawe S, Ross J, Mattick RP, Degenhardt L. The relationship between methamphetamine use and heterosexual behavior: Evidence from a prospective longitudinal study. *Addiction*, 2018; 113:1276-1285.  Meade CS, Bevilacqua LA, Moore ED, Griffin ML, Gardin JG, Potter JS, Hatch0Maillette M, Weiss RD. Concurrent substance abuse is associated with sexual risk behavior among adults seeking treatment for prescription opioid dependence. *Am J Addict.*, 2014; 23(1):27-33.  Roberts AC, Wechsberg WM, Zule W, Burroughs AR. Contextual factors and other correlates of sexual risk of HIV among African-American crack-abusing women. *Addictive Behaviors*, 2003; 28:523-536.  Rolfs RT, Goldberg M, Sharrar RG. Risk factors for syphilis: Cocaine use and prostitution. *Am J Public Health*, 1990; 80(7):853-857.  Shokoohi M, Karamouzian M, Sharifi H, Rahimi-Movaghar A, Carrico AW, Hooshyar SH, Mirzazadeh A. Crystal methamphetamine use and its correlates in women engaged in sex work in a developing country setting. *Drug and Alcohol Dependence*, 2018; 185:260-265.  Taylor MM, Aynalem G, Smith LV, Montoya J, Kerndt P. Methamphetamine use and sexual risk behaviours among men who have sex with men diagnosed with early syphilis in Los Angeles county. *Int J STD AIDS*, 2007; 18(2:93-97.  Weatherby NL, Shultz JM, Chitwood DD, McCoy HV, Ludwig DD, Edlin BR. Crack cocaine use and sexual activity in Miami, Florida. *J Psychoactive Drugs*, 1992; 24(4):373-380.  Wingwood GM, DiClemente RJ. The influence of psychosocial factors, alcohol use, drug use on African-American women's high-risk sexual behavior. *Am J Prev Med*, 1998; 15(1):54-59.  Yan AF, Chiu Y, Stoesen CA, Wang MQ. STD-/HIV-related sexual risk behaviors and substance use among U.S. rural adolescents. *J Natl Med Assoc*, 2007; 99(12):1386-1394.  **References for depression and multiple sexual partners**  Berg J, Nyamathi A, Christiani A, Morisky D, Leake B. Predictors of screening results for depressive symptoms among homeless adults in Los Angeles with latent tuberculosis. Res Nurs Health., 2005; 28(3):220-229.  Hutton HE, Lyketsos CG, Zenilman JM, Thompson RE, Erbelding EJ. Depression and HIV risk behavior among patients in a sexually transmitted disease clinic. Am J Psychiatry., 2004; 161:912-914.  Jiang H, Li J, Tan Z, Chen X, Cheng W, Gong X, Yang Y. Syndemic factors and HIV risk among men who have sex with men in Guangzhou, China: Evidence from synergy and moderated analyses. Arch Sex Behav., 2020; 49:311-320.  Khan MR, Kaufman JS, Pence BW, Gaynes BN, Adimora AA, Weir SS, Miller WC. Depression, sexually transmitted infection, and the sexual risk behavior among young adults in the United States. Arch Pediatr Adolesc Med., 2009; 163(7):644-652.  Kosunen E, Kaltiala-Heino R, Rimpela M, Laippala P. Risk-taking sexual behavior and self-reported depression in middle adolescence - a school based survey. Child Care Health Dev., 2013; 29(5):337-344.  Larsen A, Kinuthia J, Lagat H, Sila J, Abuna F, Kohler P, John-Stewart G, Pintye J. Depression and HIV risk behaviors among adolescent girls and young women seeking family planning services in Western Kenya. Int J STD AIDS., 2020; 31(7):652-664.  Lavan H, Johnson JG. The association between axis I and axis II psychiatric symptoms and high-risk sexual behavior during adolescence. J Pers Disord., 2002; 16(1):73-94.  Lerand SJ, Ireland M, Blum BW. Individual and environmental impacts on sexual health of Caribbean youth. ScienctificWorldJournal., 2006; 6:707-717.  Martinez I, Kershaw TS, Lewis JB, Stasko EC, Tobin JN. Ickovics JR. Between synergy and travesty: A sexual risk syndemic among pregnant Latina immigrant and non-immigrant adolescents. AIDS Behav., 20187; 21:858-869.  McCusker J, Goldstein R, Bigelow C, Zorn M. Psychiatric status and HIV risk reduction among residential drug abuse treatment clients. Addiction., 1995; 90:1377-1387.  Nduna M, Jewkes RK, Dunkle KL, Jama Shai NP, Colman I. Associations between depressive symptoms, sexual behaviour and relationship characteristics: a prospective cohort study of young women and men in Eastern Cape, South Africa. J Int AIDS Soc., 2010; 13:44.  Newville H, Haller DL. Relationship of axis II pathology to sex- and drug-related risk behaviors among patients in HIV primary care. AIDS Care., 2012; 24(6):763-768.  Nyamathi AM, Bennett C, Leake B. Predictors of maintained high-risk behaviors among impoverished women. Public Health Rep., 1995; 110:600-606.  Okafor CN, Christodoulou J, Bantjes J, Qondela T, Stewart J, Shoptaw S, Tomlinson M, Rotherman-Borus M. Understanding HIV risk behaviors among young men in South Africa: A syndemic approach. AIDS Behav., 2018; 22(12):3962-3970.  Sabri B, McFall AM, Solomon SS, Srikrishnan AK, Vasudevan CK, Anand S, Celentano DD, Mehta SH, Kumar S, Lucas GM. Gender differences in factors related to HIV risk behaviors among people who inject drugs in North-East India. PLoS One., 2017.  Savoija H, Helminen M, Frojd S, Marttunen M, Kaltiala-Heino R. Deliquency and sexual experiences across adolescence: Does depression play a role? Eur J Contracept Reprod Health Care., 2017; 22(4):298-304.  Seth P, Patel SN, Sales JM, DiCelemente RJ, Wingood GM, Rose ES. The impact of depressive symptomatology on risky sexual behavior and sexual communication among African American female adolescents. Psychol Health Med., 2011; 16(3):346-356.  Smit J, Myer L, Middelkoop K, Seedat S, Wood R, Bekker LG, Stein DJ. Mental health and sexual risk behaviours in a South African township: A community-based cross-sectional study. Public Health, 2006; 120:534-542.  Turner AK, Latkin C, Sonenstein F, Tandon SD. Psychiatric disorder symptoms, substance use, and sexual risk behavior among African-American out of school youth. Drug Alcohol Depend., 2011; 115(1-2):67-73.  Wang Z, Zhao X, Zhang Z, Luo M, Shen Q, Dong Y, Wang Y, Cai Y. Co-occuring psychological problems and multiple sexual partners among men who have sex with men in Shanghai, China: A syndemic approach. J Sex Res., 2018; 55(7):892-901.  Wickrama T, Wickrama KAS. Heterogeneity in adolescent depressive symptom trajectories: Implications for young adults' risk lifestyle. J Adolesc Health., 2010; 47:407-413.  **References for anxiety and multiple sexual partners**  Kugbey, N., Ayanore, M. A., Amu, H., Oppong Asante, K., & Adam, A. (2018). International note: Analysis of risk and protective factors for risky sexual behaviours among school-aged adolescents. *Journal of adolescence*, *68*, 66–69. <https://doi.org/10.1016/j.adolescence.2018.06.013> (study 7)  Ramrakha, S., Paul, C., Bell, M. L., Dickson, N., Moffitt, T. E., & Caspi, A. (2013). The relationship between multiple sex partners and anxiety, depression, and substance dependence disorders: a cohort study. *Archives of sexual behavior*, *42*(5), 863–872. <https://doi.org/10.1007/s10508-012-0053-1> (study 12)  Turner, A. K., Latkin, C., Sonenstein, F., & Tandon, S. D. (2011). Psychiatric disorder symptoms, substance use, and sexual risk behavior among African-American out of school youth. *Drug and alcohol dependence*, *115*(1-2), 67–73. <https://doi.org/10.1016/j.drugalcdep.2010.10.012> (study 16)  Lavan, H., & Johnson, J. G. (2002). The association between axis I and II psychiatric symptoms and high-risk sexual behavior during adolescence. *Journal of personality disorders*, *16*(1), 73–94. <https://doi.org/10.1521/pedi.16.1.73.22559> (study 42)  **Reference for pain and multiple sexual partners**  Vu, T.M.T., Boggiano, V.L., Tran, B.X., Nguyen, L.H., Tran, T.T., Latkin, C.A., Ho, C.S.H., Ho, R.C.M. (2018). Sexually risk behavior of patients with HIV/AIDS over the course of antiretroviral treatment in northern Vietnam. Int. J. Environ. Res. Public Health, 15, 1106. |
| --- |

# Figure S27: Meta-analysis forest plot: alcohol and medication non-adherence

**
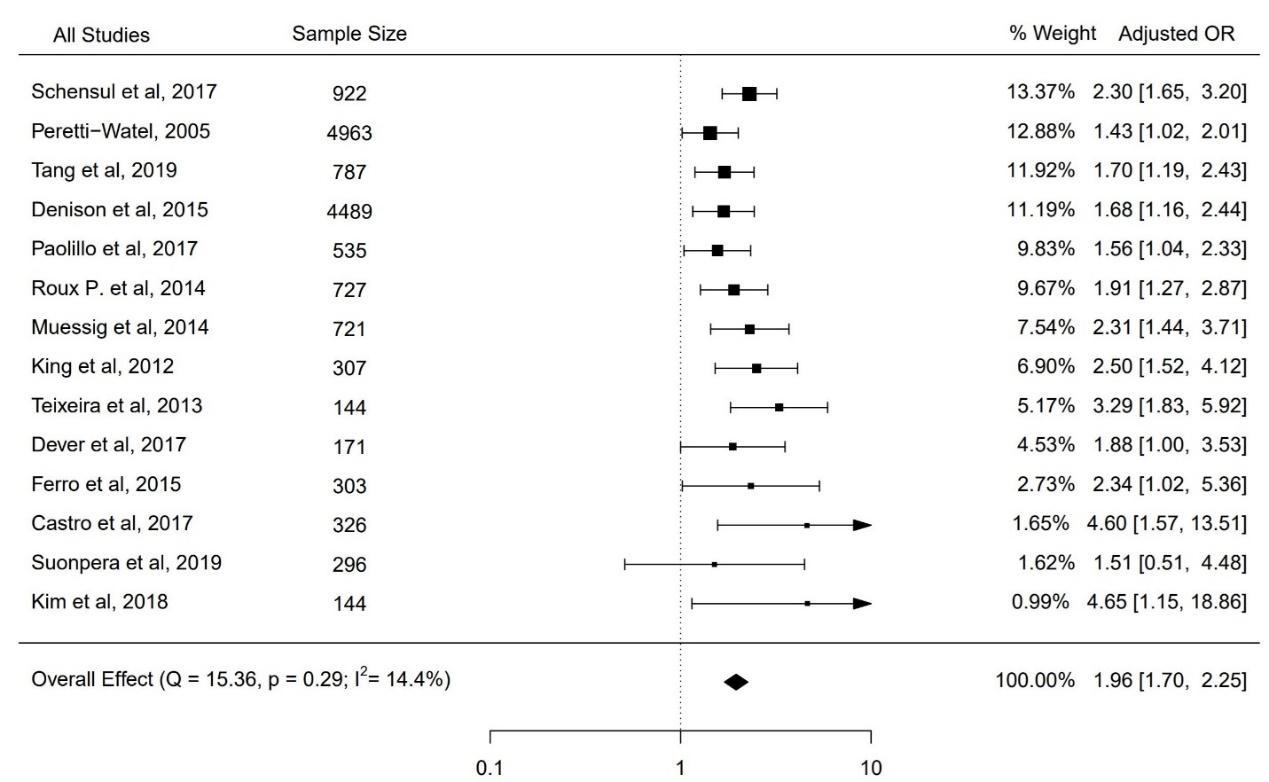
**

# Figure S28: Meta-analysis forest plot: depression and medication non-adherence

**
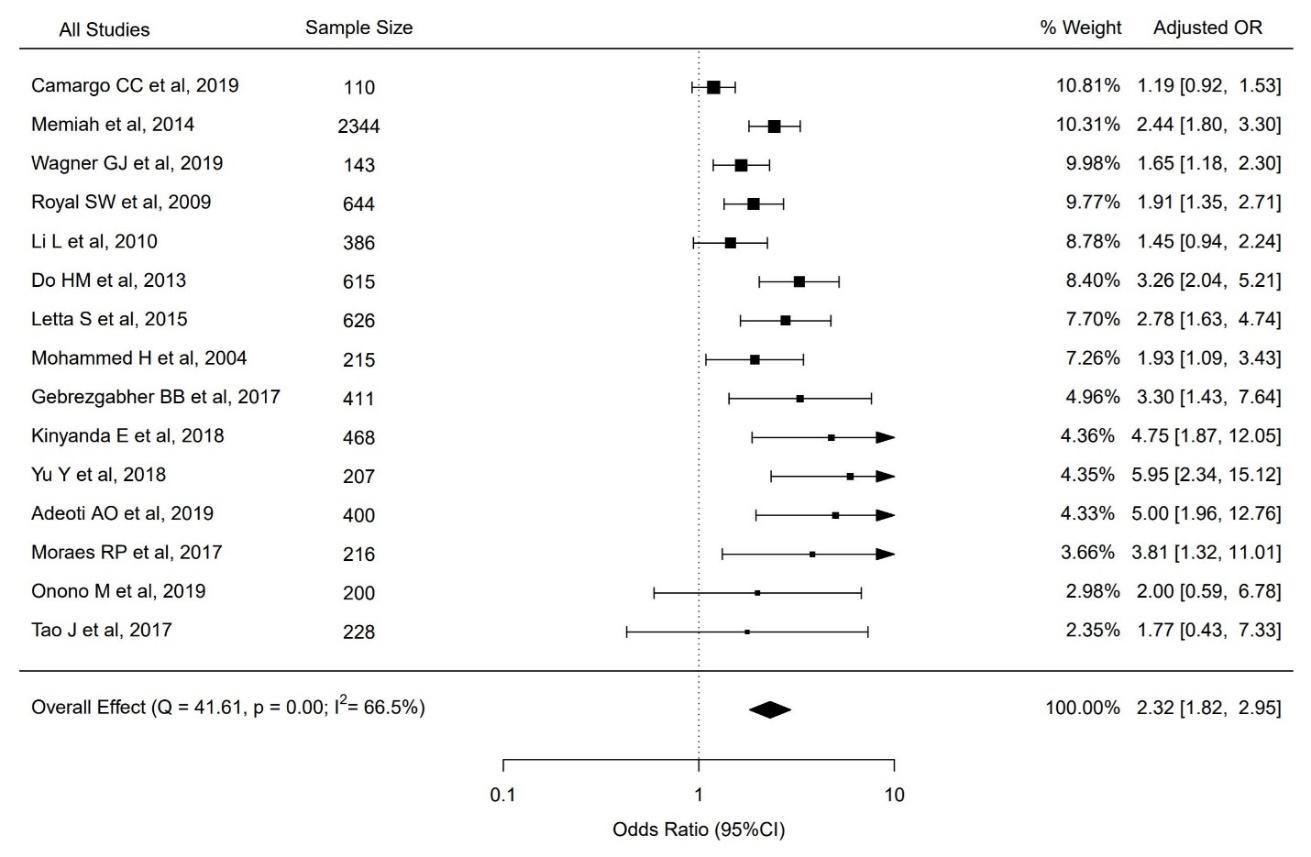
**

# Figure S29: Meta-analysis forest plot: anxiety and medication non-adherence

**
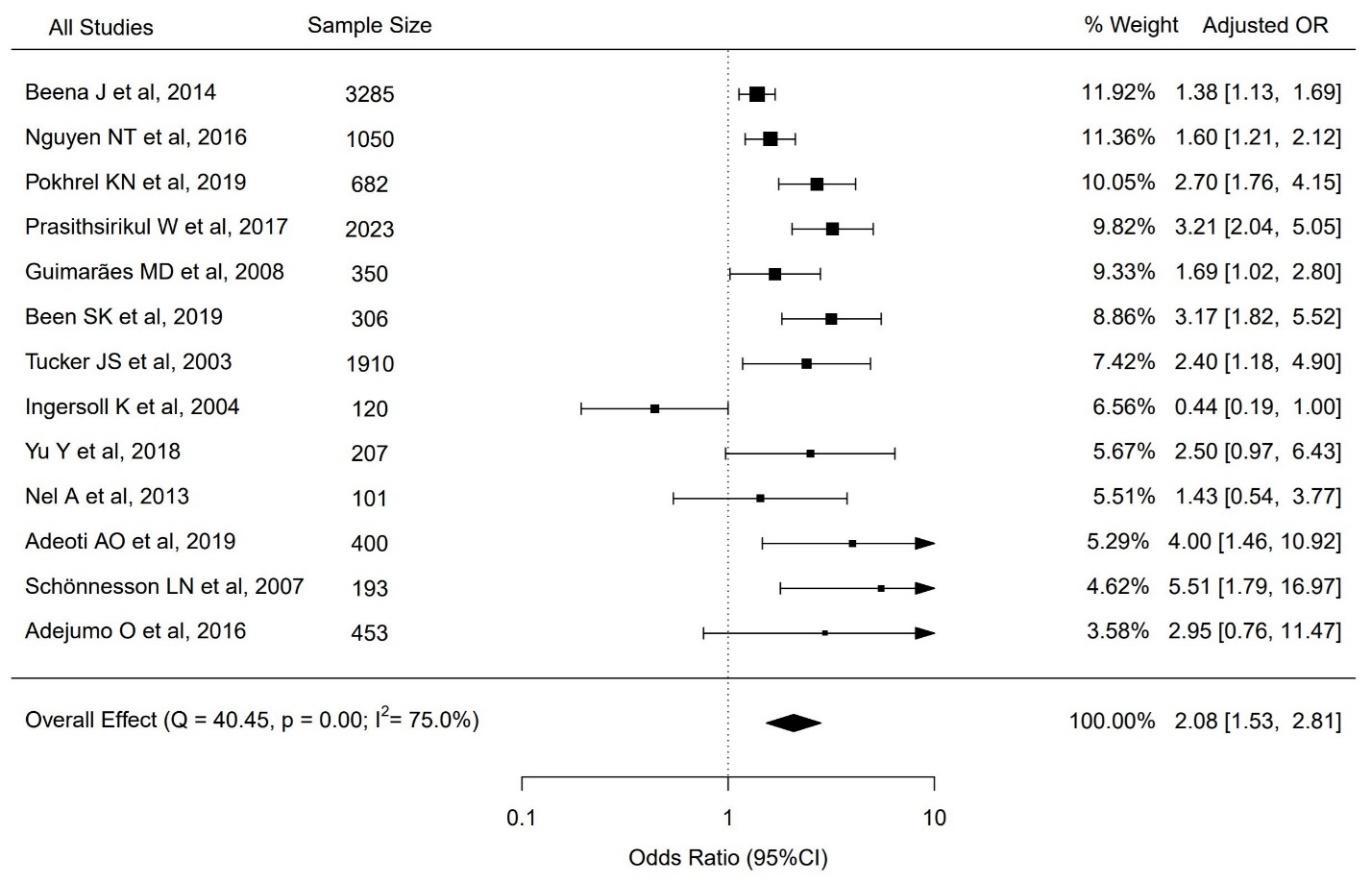
**

# Figure S30: Meta-analysis forest plot: pain and medication non-adherence

**
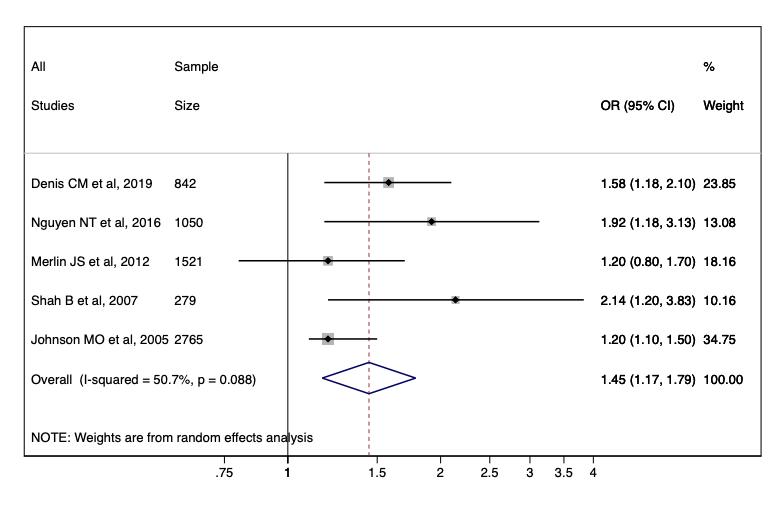
**

# Figure S31: Meta-analysis forest plot: tobacco and medication non-adherence


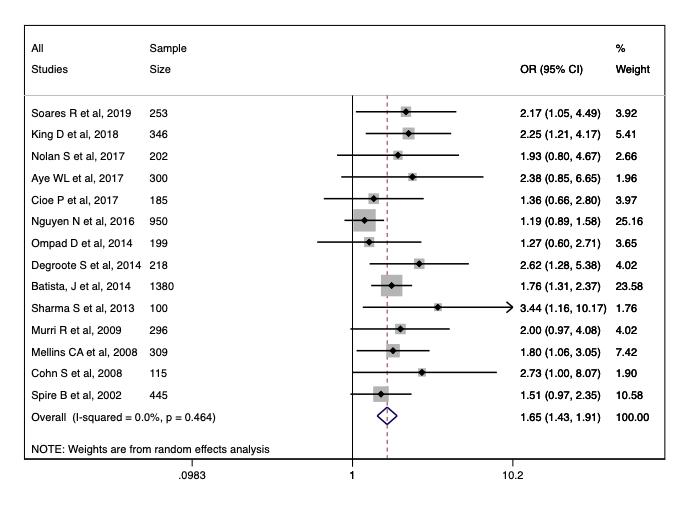


# Figure S32: Meta-analysis forest plot: opioids and medication non-adherence


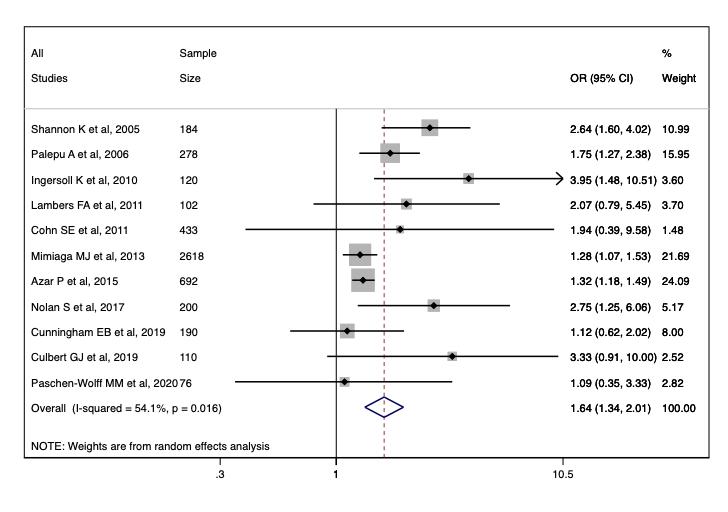


*Note: meta-analysis output in Stata reported the overall OR to be 1.643 (1.345-2.008); the discrepancy in the above forest plot (reporting OR 95% CI LL 1.34) and summary results table in the main text (OR 95% CI LL 1.35) is due to a rounding inconsistency in Stata.*

# Figure S33: Meta-analysis forest plot: stimulants and medication non-adherence

**
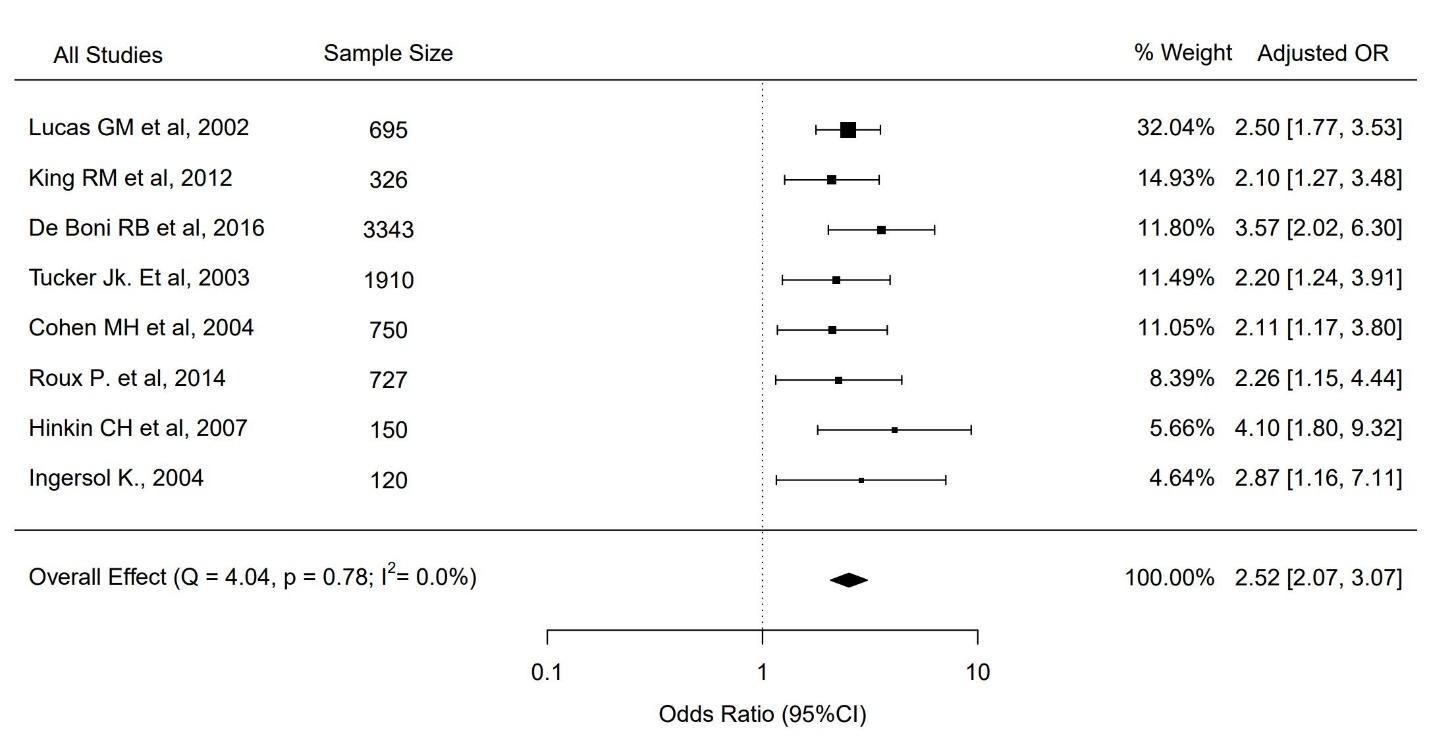
**

# Figure S34: Meta-analysis forest plot: alcohol and unprotected sex

**
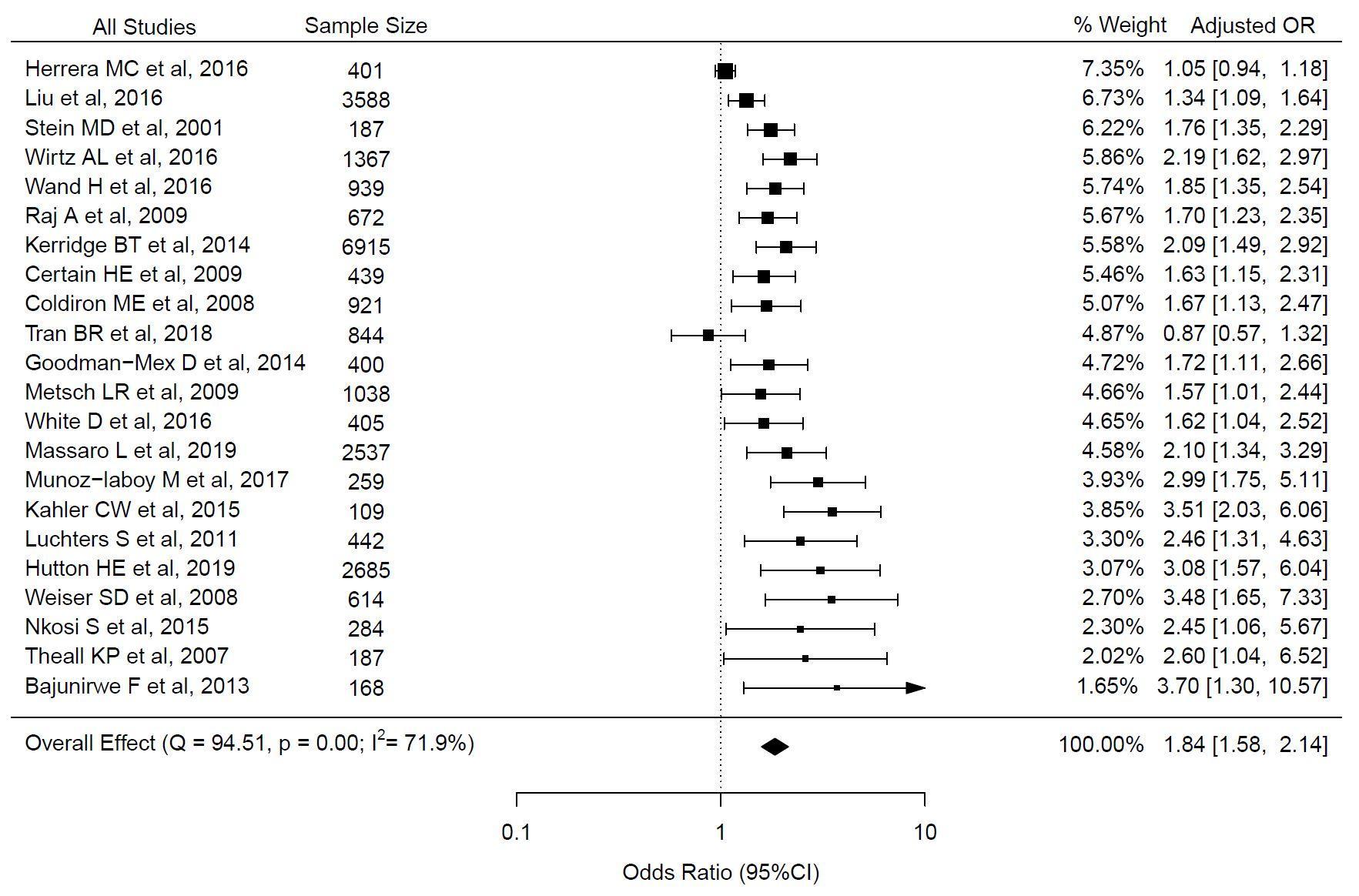
**

# Figure S35: Meta-analysis forest plot: depression and unprotected sex

**
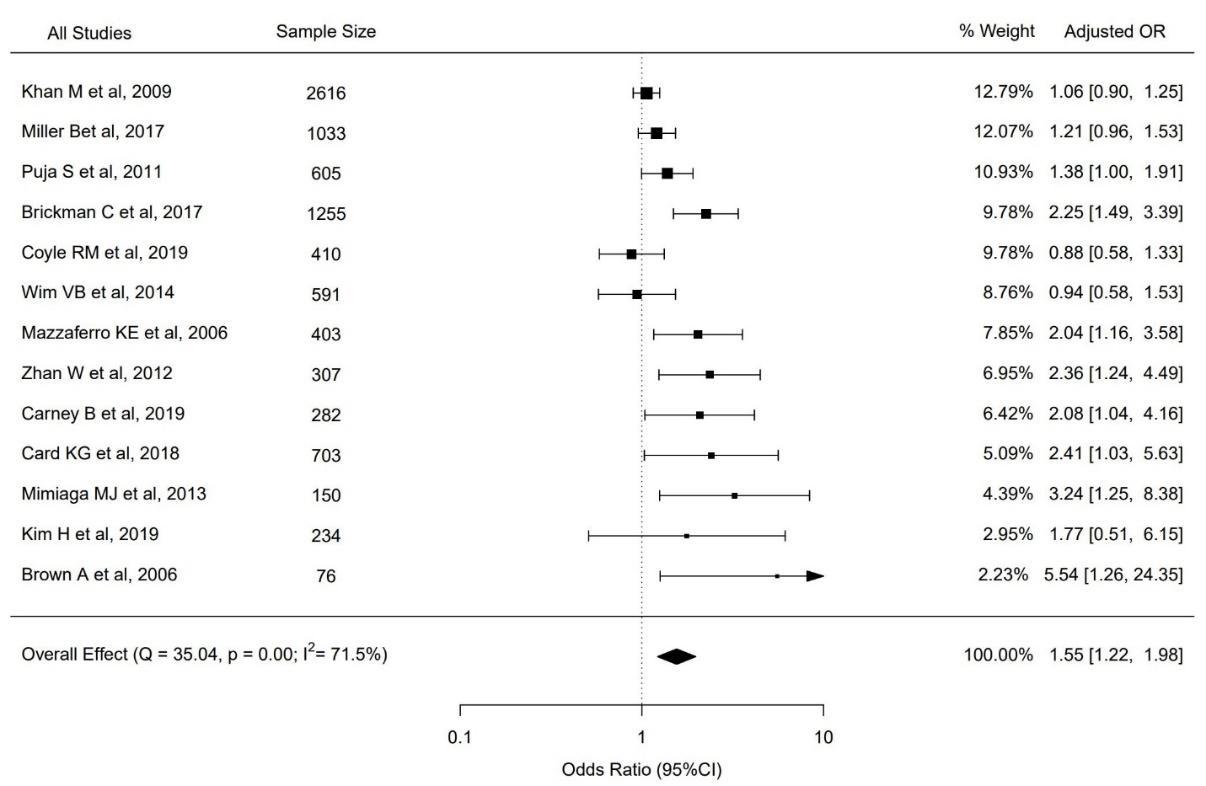
**

# Figure S36: Meta-analysis forest plot: anxiety and unprotected sex


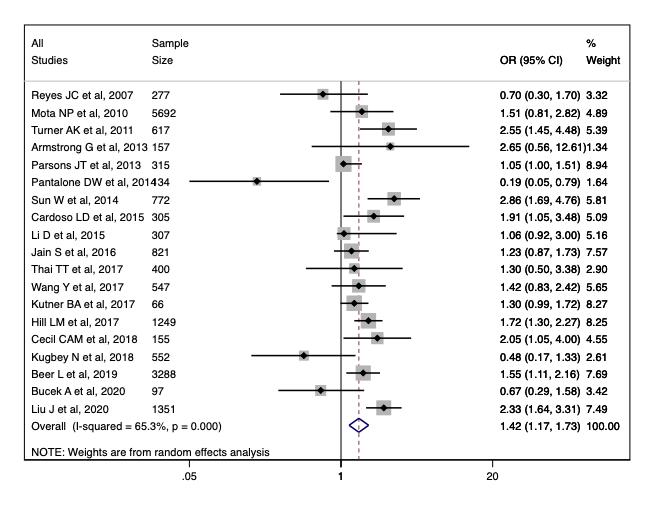


# Figure S37: Meta-analysis forest plot: pain and unprotected sex

**
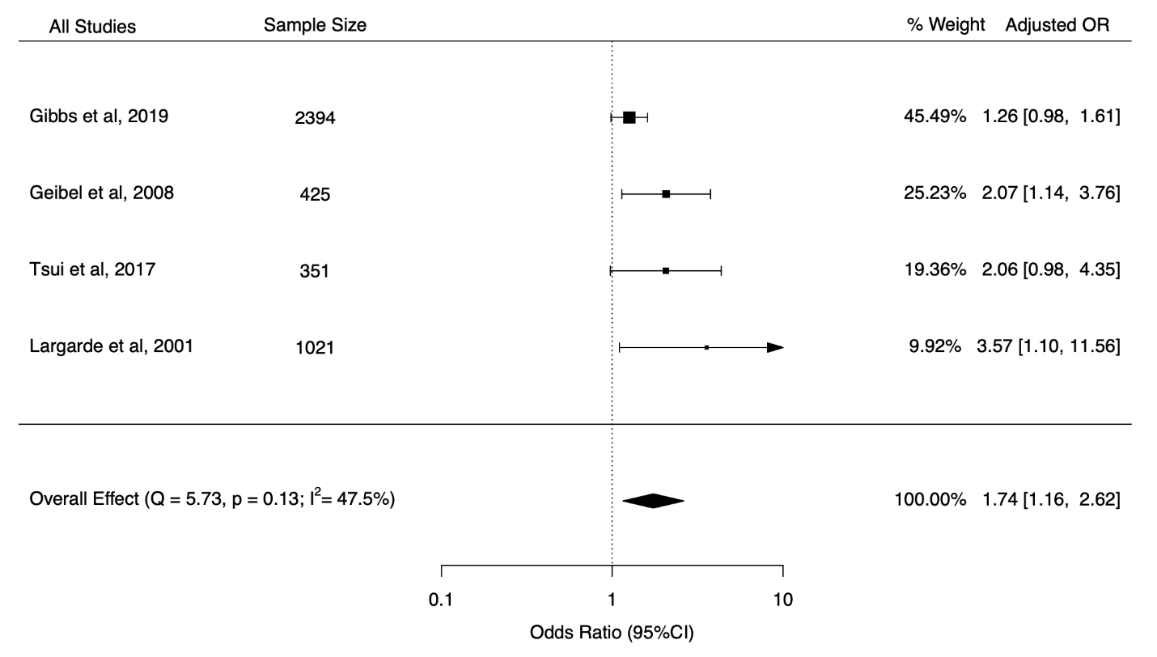
**

# Figure S38: Meta-analysis forest plot: tobacco and unprotected sex


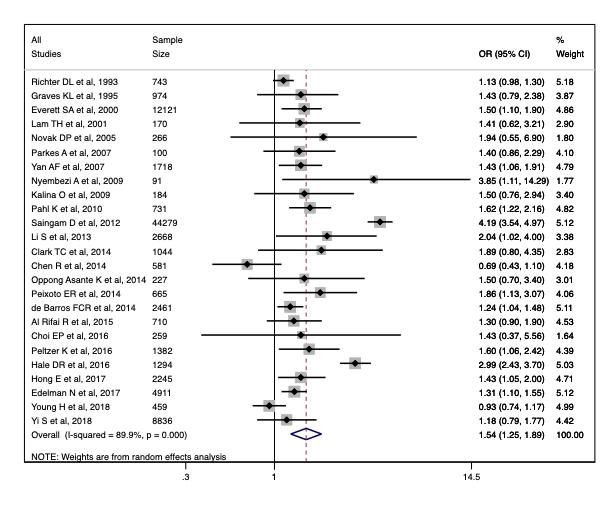


# Figure S39: Meta-analysis forest plot: opioids and unprotected sex


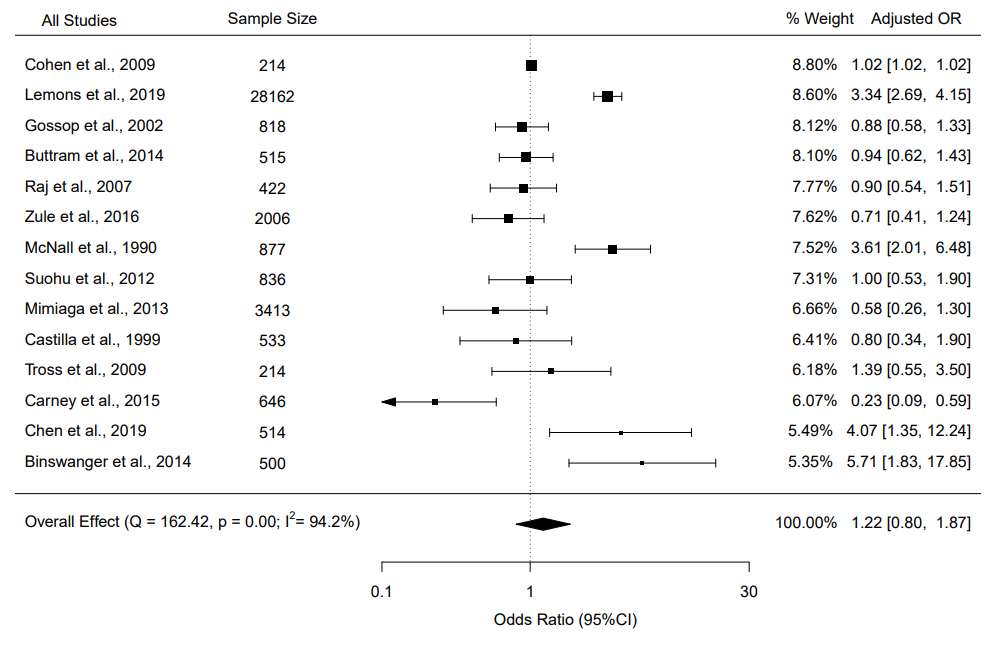


# Figure S40: Meta-analysis forest plot: stimulants and unprotected sex

**
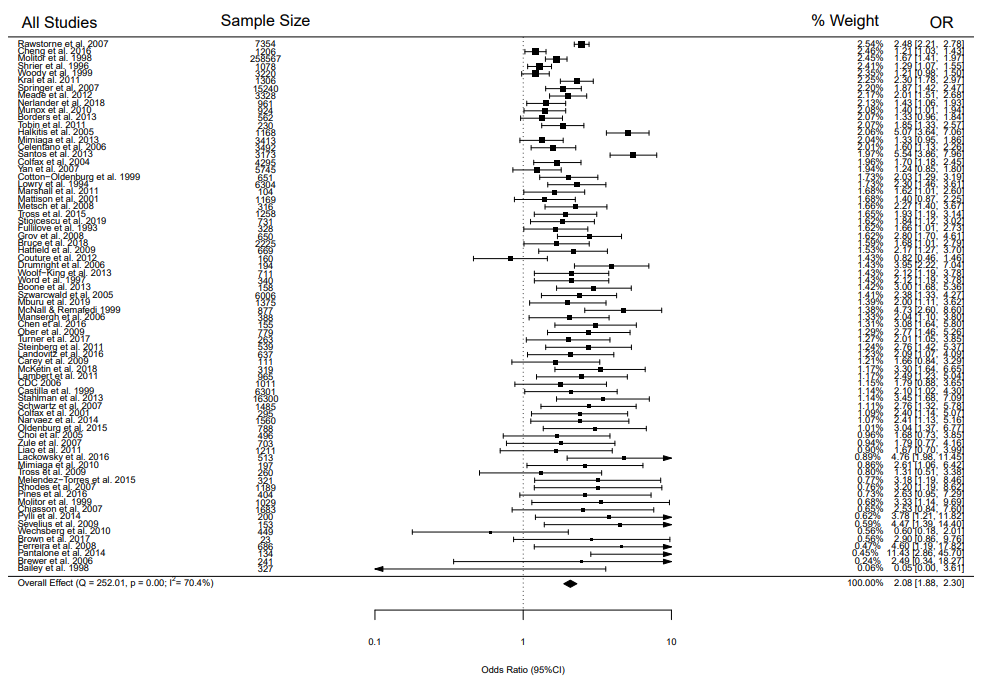
**

# Figure S41: Meta-analysis forest plot: alcohol and transactional sex

**
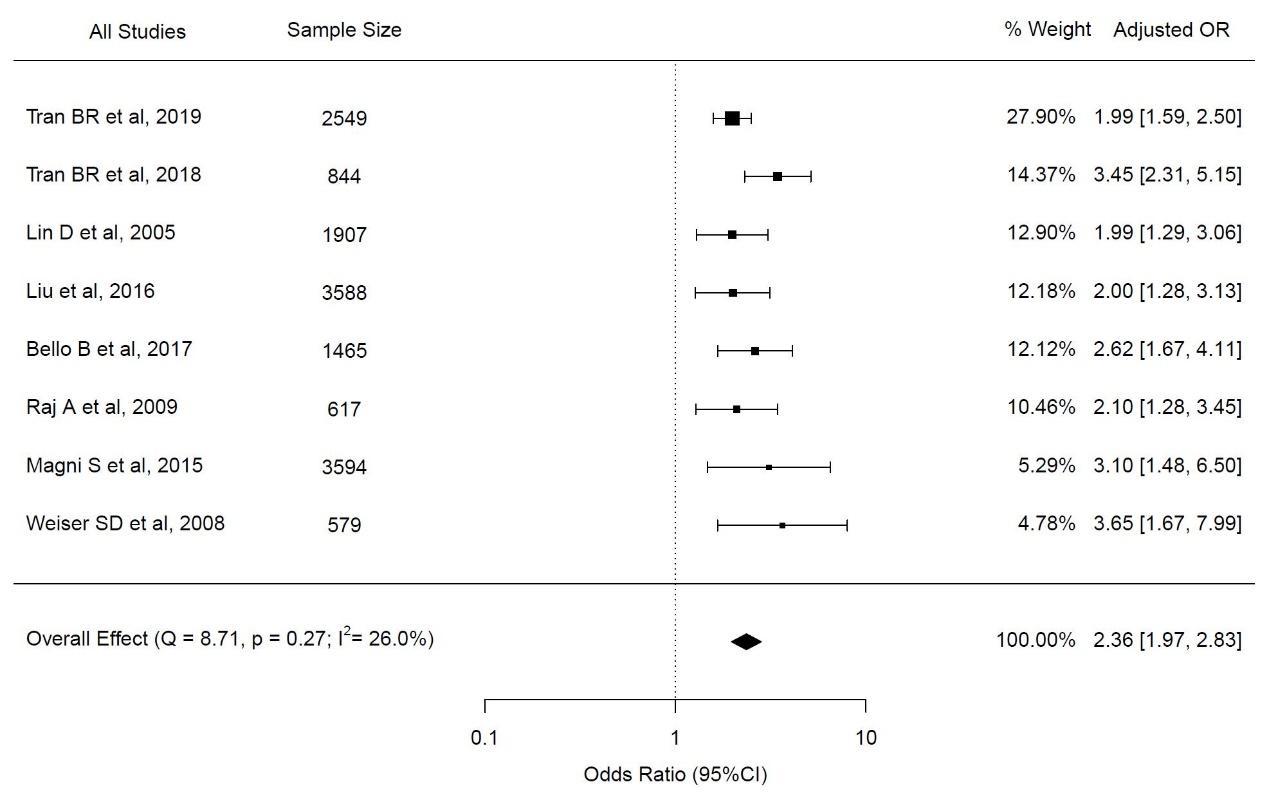
**

# Figure S42: Meta-analysis forest plot: depression and transactional sex

**
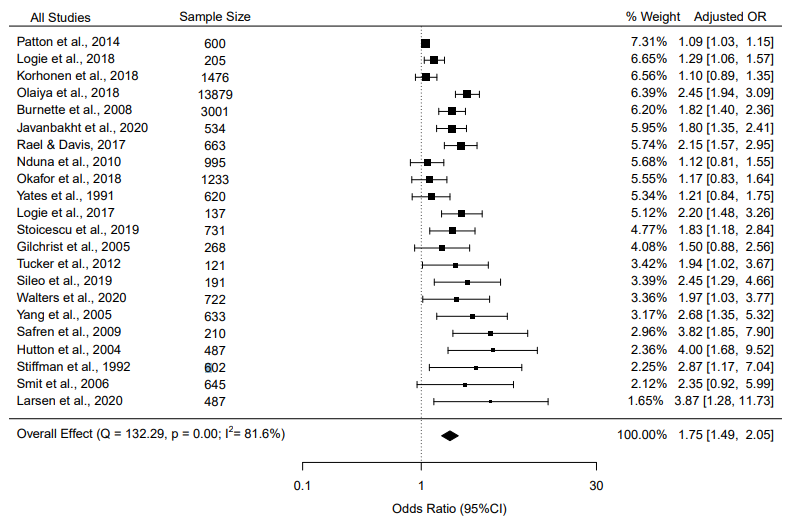
**

# Figure S43: Meta-analysis forest plot: anxiety and transactional sex


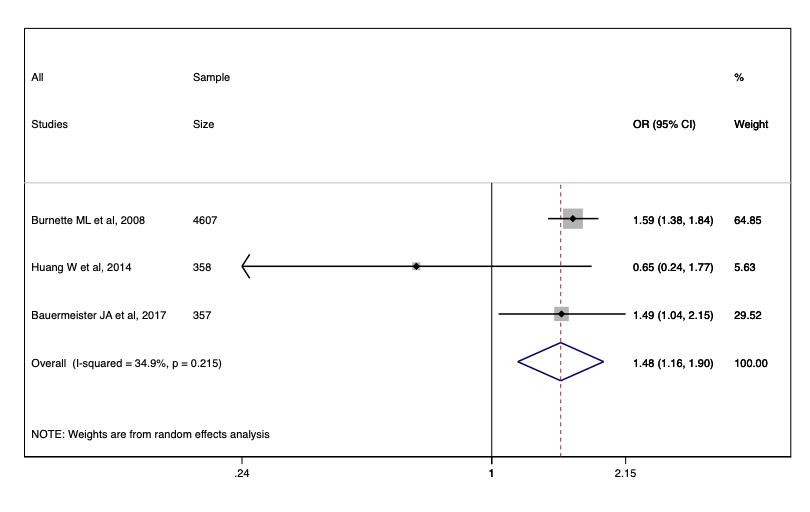


**NOTE:** Pain and transactional sex yielded no eligible studies.

# Figure S44: Meta-analysis forest plot: tobacco and transactional sex


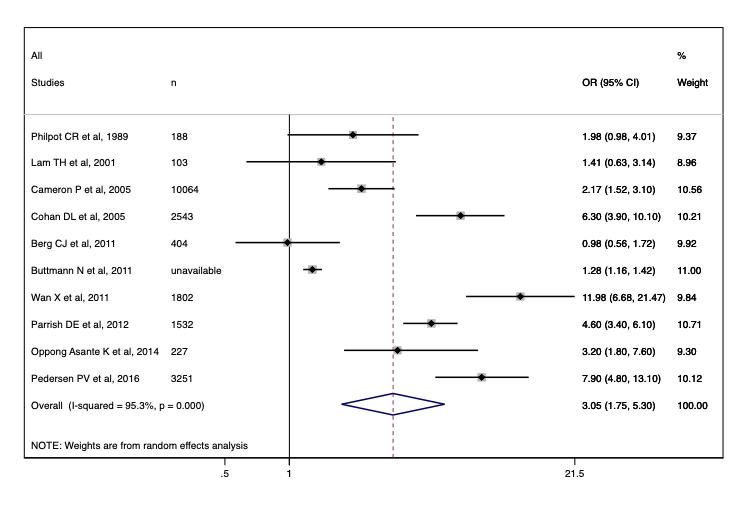


# Figure S45: Meta-analysis forest plot: opioids and transactional sex

**
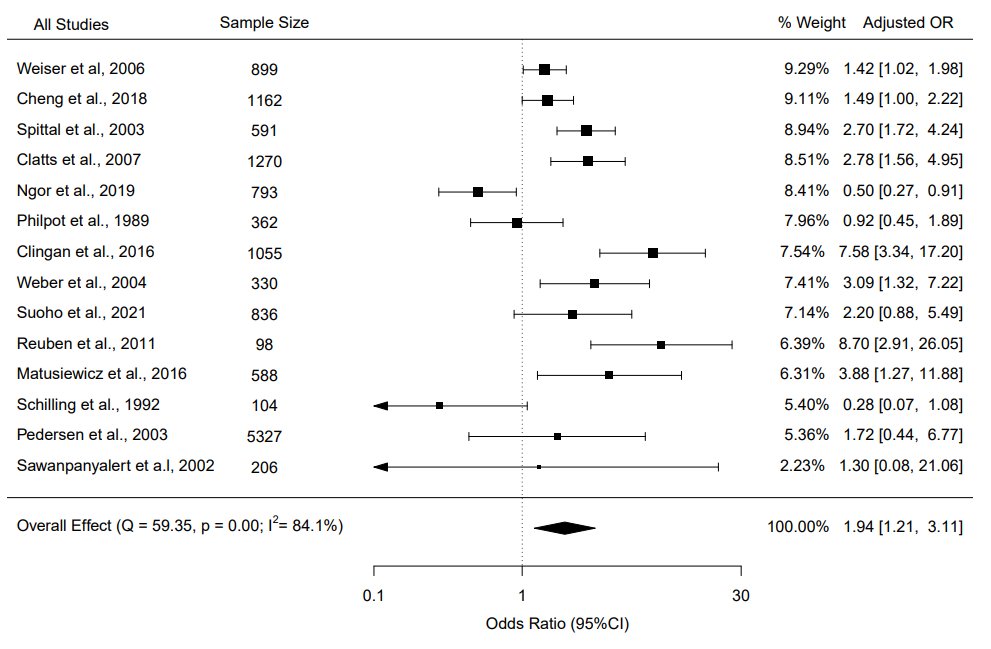
**

# Figure S46: Meta-analysis forest plot: stimulants and transactional sex

**
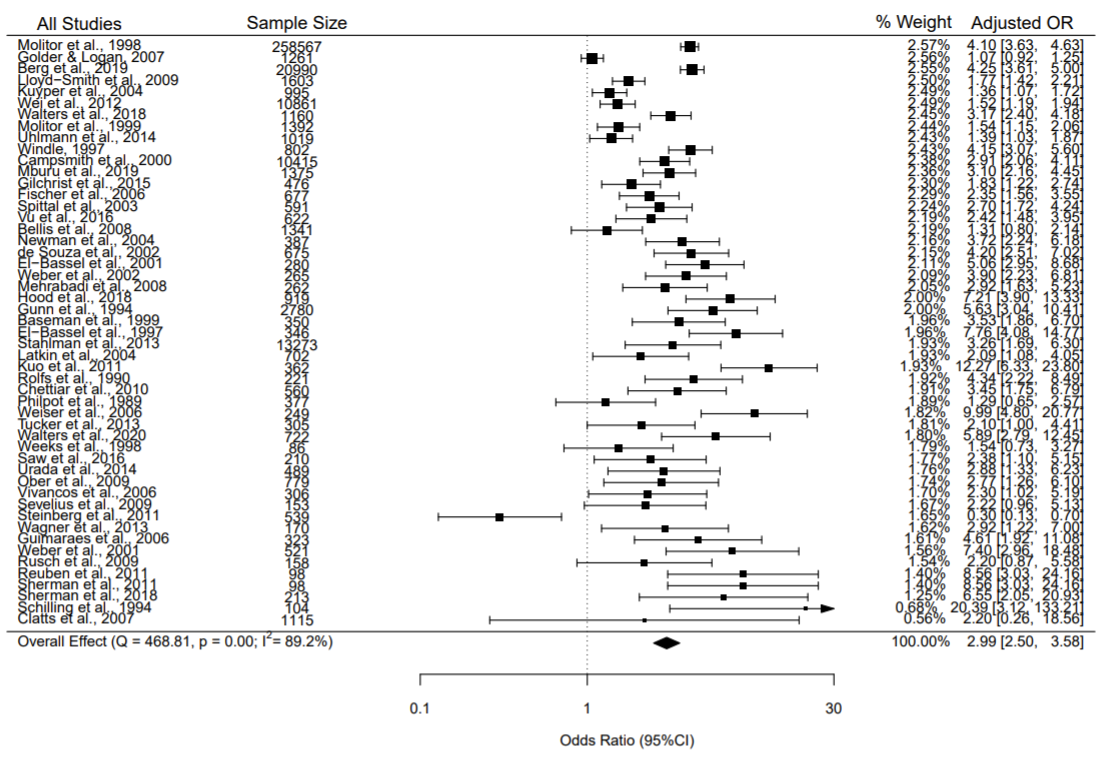
**

# Figure S47: Meta-analysis forest plot: alcohol and multiple sexual partners

**
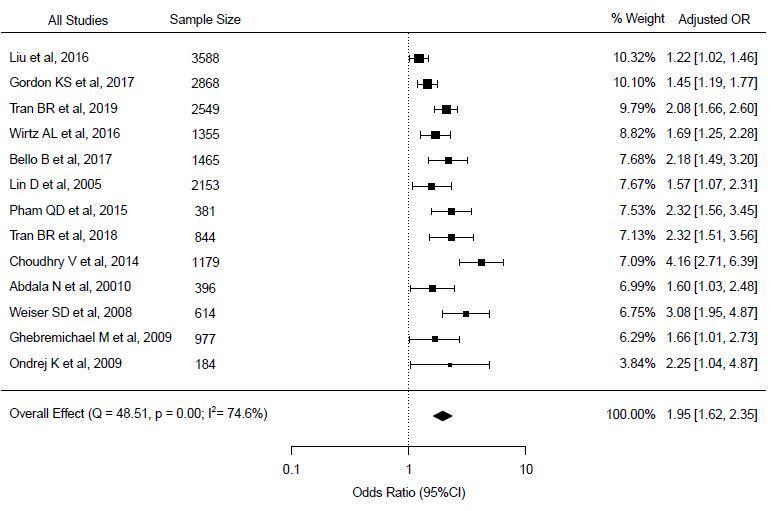
**

# Figure S48: Meta-analysis forest plot: depression and multiple sexual partners

**
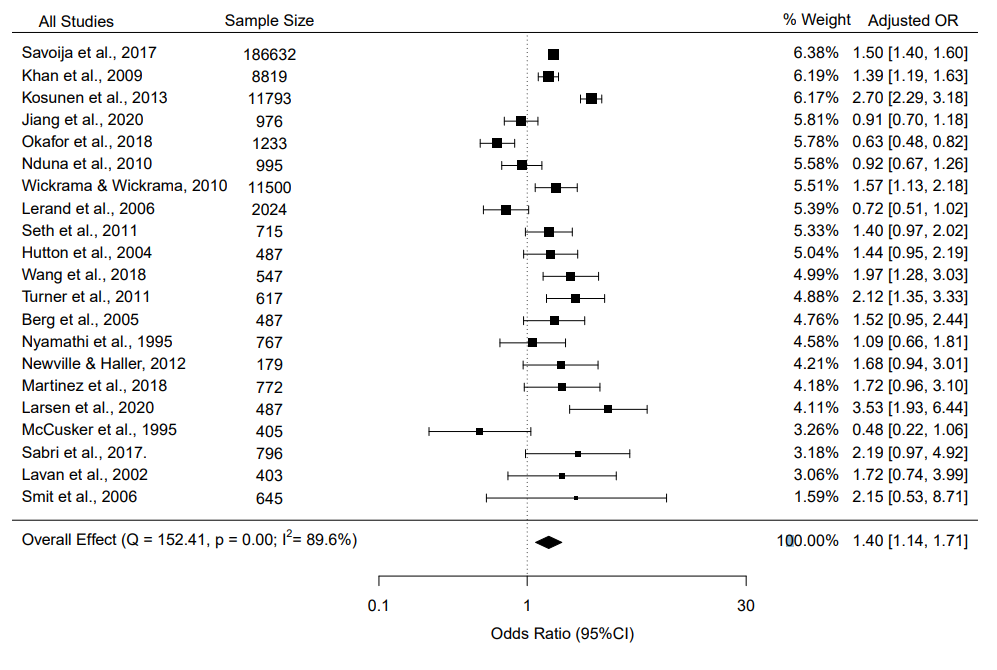
**

# Figure S49: Meta-analysis forest plot: anxiety and multiple sexual partners


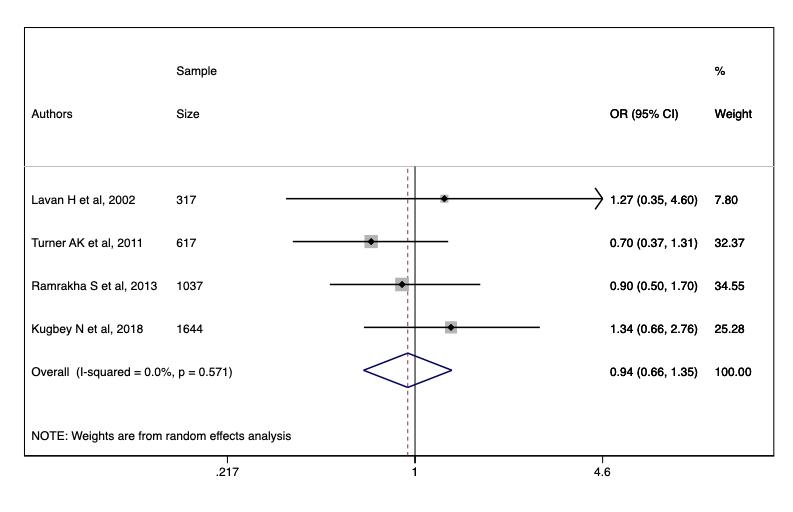


**NOTE:** Pain and multiple sexual partners yielded a single study.

# Figure S50: Meta-analysis forest plot: tobacco and multiple sexual partners


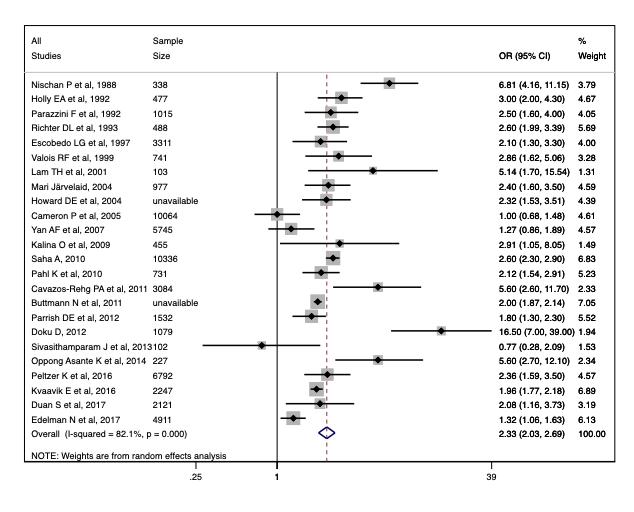


*Note: meta-analysis output in Stata reported the overall OR to be 2.335 (2.030-2.685); the discrepancy in the above forest plot (reporting OR 2.33) and summary results table in the main text (OR 2.34) is due to a rounding inconsistency in Stata.*

# Figure S51: Meta-analysis forest plot: opioids and multiple sexual partners

**
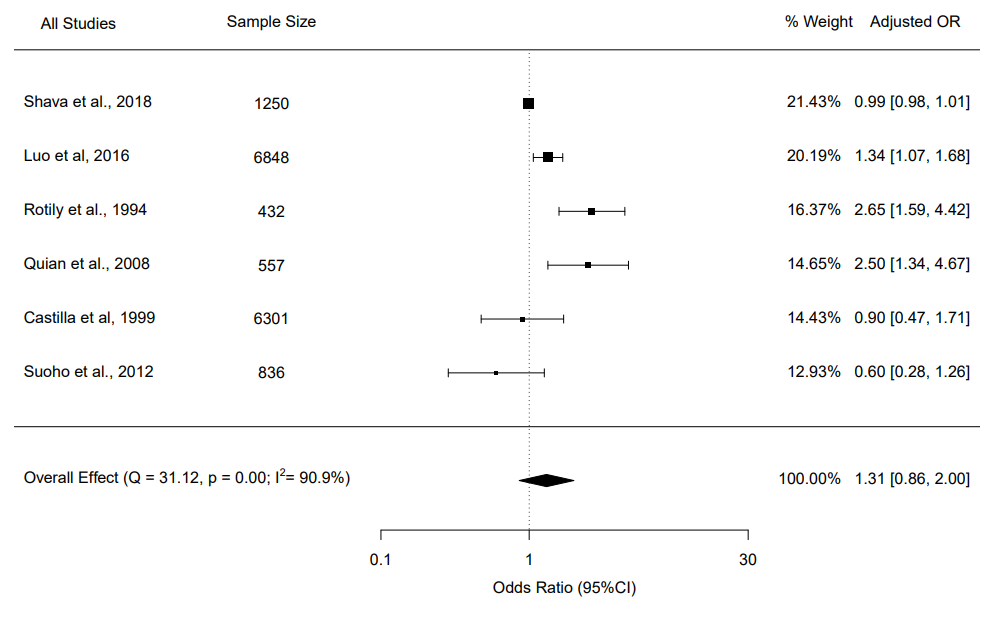
**

# Figure S52: Meta-analysis forest plot: stimulants and multiple sexual partners

**
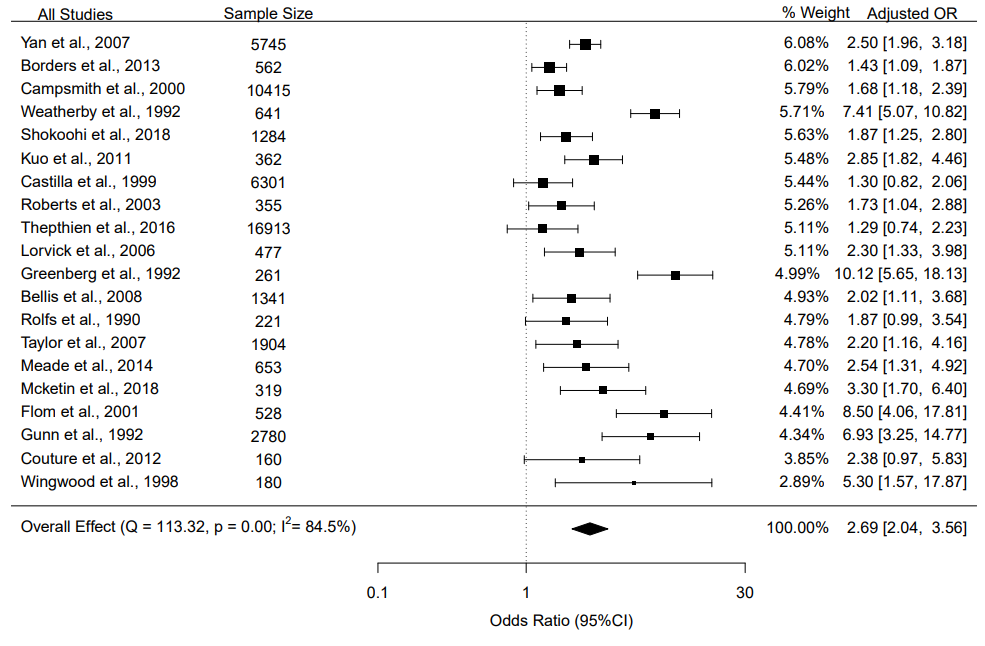
**

# Table S1: Heterogeneity values from statistical output of meta-analyses eligible for the proportion of meaningfully strong effects sensitivity analysis

| **Meta-analysis exposure** | **Meta-analysis outcome** | **Estimate of heterogeneity (τ²)** |
| --- | --- | --- |
| *Unrestricted meta-analyses* | | |
| Alcohol | Medication non-adherence | 0.01 |
| Depression | Medication non-adherence | 0.125 |
| Anxiety | Medication non-adherence | 0.191 |
| Opioids | Medication non-adherence | 0.0398 |
| Alcohol | Unprotected sex | 0.079 |
| Depression | Unprotected sex | 0.112 |
| Anxiety | Unprotected sex | 0.098 |
| Tobacco | Unprotected sex | 0.2132 |
| Opioids | Unprotected sex | 0.53 |
| Stimulants | Unprotected sex | 0.1 |
| Depression | Transactional sex | 0.092 |
| Tobacco | Transactional sex | 0.7217 |
| Opioids | Transactional sex | 0.60 |
| Stimulants | Transactional sex | 0.33 |
| Alcohol | Multiple sexual partners | 0.079 |
| Depression | Multiple sexual partners | 0.17 |
| Tobacco | Multiple sexual partners | 0.0712 |
| Stimulants | Multiple sexual partners | 0.32 |
| *CASM-adjusted meta-analyses* | | |
| Stimulants | Unprotected sex | 0.06 |
| Stimulants | Transactional sex | 0.21 |
| Tobacco | Multiple sexual partners | 0.0167 |

# Figure S53: Secondary analysis: meta-analysis results (restricted to CASM-adjusted studies)

**
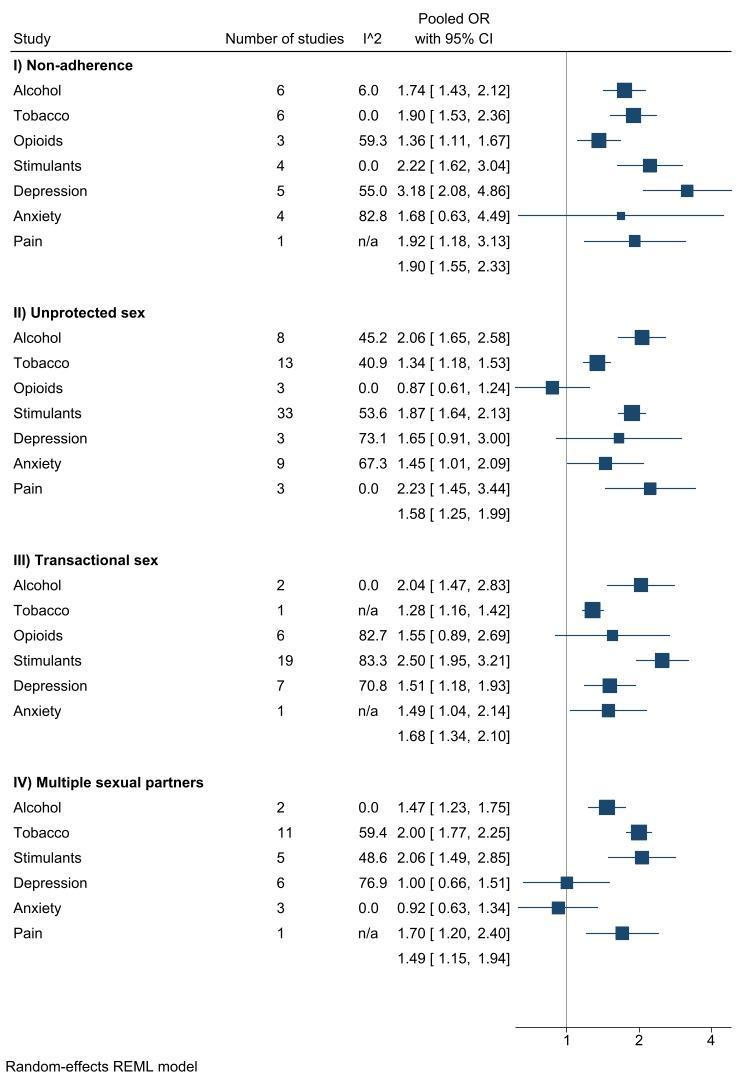
**

#

# Supplementary Box 3: Secondary analysis systematic review and meta-analysis notes

| Of the 28 associations of interest, the secondary analysis comprised 25 sets of associations (primary effect estimate vs CASM-adjusted-constituent-studies-effect estimate), since pain and transactional sex yielded no relevant studies, opioids and multiple sexual partners did not have CASM-adjusted estimates, and pain and multiple sexual partners had a single CASM-adjusted study with no comparator. Three CASM-adjusted effect estimates comprised a single CASM-adjusted study and should be interpreted with caution. Uncertainty ranges are generally wider, but do not demonstrate strong evidence of a reversal in direction of effect in most cases. I^2^ values are generally lower than in the primary analysis, potentially reflected by lower study counts.  Secondary analysis indicates 73% (19/26) of pooled odds ratios show strong evidence of a positive association, and 84% (21/25) of meta-analysis-derived estimates have notable confounding by other CASM conditions (defined by a ≥ 0.20 change-in-effect between the primary meta-analysis OR and the CASM-adjusted-subgroup meta-analysis OR). Most notable shifts, 76% (16/21), occurred towards the null, suggesting positive confounding by other CASM. 100% (15/15) of ATOS sets (comparing CASM adjusted-subgroup meta-analysis ORs to primary analyses ORs) indicated notable confounding by CASM, compared to 60% (6/10) for DAP association sets. Among those with notable shifts, 87% (13/15) of ATOS association sets showed positive confounding by other CASM, while 50% (3/6) of DAP association sets showed this. While all transactional sex and multiple sexual partner association sets indicate positive confounding by other CASM, only 57% (4/7) of medication non-adherence and 60% (3/5) of unprotected sex association sets reflect this. |
| --- |

# Table S2: Secondary analysis: assessing unmeasured confounding in meta-analyses restricted to CASM-adjusted estimates, by the point estimate sensitivity analysis, excluding studies assessed by proportion of meaningfully strong effects

| **Exposure** | **Outcome** | **Pooled RR**  **(95% CI)^1^** | **E-value**  **(95% CI LL)** | **Bias factor** | **Bias-adjusted RR (95% CI)** |
| --- | --- | --- | --- | --- | --- |
| Alcohol | Non-adherence | 1.32 (1.2-1.46) | 1.97 (1.68) | 1.11 | 1.19 (1.08-1.31) |
| Tobacco | Non-adherence | 1.38 (1.24-1.54) | 2.10 (1.78) | 1.11 | 1.24 (1.11-1.39) |
| Tpioids | Non-adherence | 1.17 (1.05-1.29) | 1.61 (1.29) | 1.11 | 1.05 (0.95-1.16) |
| Stimulants | Non-adherence | 1.49 (1.27-1.74) | 2.34 (1.86) | 1.11 | 1.34 (1.15-1.57) |
| Depression | Non-adherence | 1.78 (1.44-2.2) | 2.97 (2.24) | 1.01 | 1.77 (1.43-2.19) |
| Anxiety | Non-adherence | 1.30 (0.79-2.12) | 1.92 (1.00) | 1.01 | 1.29 (0.79-2.11) |
| Pain^2^ | Non-adherence | 1.39 (1.09-1.77) | 2.12 (1.39) | 1.01 | 1.38 (1.08-1.76) |
| Alcohol | Unprotected sex | 1.44 (1.28-1.61) | 2.23 (1.89) | 1.17 | 1.23 (1.10-1.37) |
| Tobacco | Unprotected sex | 1.16 (1.09-1.24) | 1.58 (1.39) | 1.17 | 0.99 (0.93-1.06) |
| Opioids | Unprotected sex | 0.93 (0.78-1.11) | 1.35 (1.00) | 1.17 | 1.09 (0.91-0.30) |
| Depression | Unprotected sex | 1.28 (0.95-1.73) | 1.89 (1.00) | 1.01 | 1.27 (0.95-1.72) |
| Anxiety | Unprotected sex | 1.20 (1.00-1.45) | 1.70 (1.08) | 1.01 | 1.19 (1.00-1.44) |
| Pain | Unprotected sex | 1.49 (1.2-1.85) | 2.35 (1.69) | 1.01 | 1.48 (1.19-1.83) |
| Alcohol | Transactional sex | 1.43 (1.21-1.68) | 2.21 (1.72) | 1.17 | 1.22 (1.04-1.43) |
| Tobacco^2^ | Transactional sex | 1.13 (1.08-1.19) | 1.52 (1.37) | 1.17 | 0.97 (0.92-1.02) |
| Opioids | Transactional sex | 1.24 (0.94-1.64) | 1.80 (1.00) | 1.17 | 1.06 (0.81-1.40) |
| Depression | Transactional sex | 1.23 (1.09-1.39) | 1.76 (1.39) | 1.01 | 1.22 (1.08-1.38) |
| Anxiety^2^ | Transactional sex | 1.22 (1.02-1.47) | 1.74 (1.16) | 1.01 | 1.21 (1.01-1.45) |
| Alcohol | Multiple sexual partners | 1.21 (1.11-1.33) | 1.72 (1.47) | 1.17 | 1.04 (0.95-1.13) |
| Tobacco | Multiple sexual partners | 1.41  (1.33-1.5) | 2.18 (1.99) | 1.17 | 1.21 (1.14-1.28) |
| Stimulants | Multiple sexual partners | 1.44 (1.22-1.69) | 2.23 (1.74) | 1.17 | 1.23 (1.04-1.44) |
| Depression | Multiple sexual partners | 1.00 (0.81-1.23) | 1.00 (1.00) | 1.01 | 0.99 (0.82-1.22) |
| Anxiety | Multiple sexual partners | 0.96 (0.79-1.16) | 1.25 (1.00) | 1.01 | 0.97 (0.80-1.17) |
| Pain^2^ | Multiple sexual partners | 1.30  (1.1-1.55) | 1.93 (1.42) | 1.01 | 1.29 (1.09-1.54) |

^1^converted from pooled ORs of appendix F

^2^ single study

# Table S3: Secondary analysis: assessing unmeasured confounding in meta-analyses restricted to CASM-adjusted estimates, by proportion of meaningfully strong effects sensitivity analysis

| **Exposure** | **Outcome** | **Pooled RR**  **(95% CI)^1^** | **E-value**  **(95% CI LL)** | **Bias factor** | **p̂(q)** | **T̂(r,q)** | **Ĝ(r,q)** |
| --- | --- | --- | --- | --- | --- | --- | --- |
| stimulants | transactional sex | 1.58 (1.40-1.79) | 2.54 (2.14) | 1.17 | 0.842 | 1.868 | 3.141 |
| stimulants | unprotected sex | 1.37 (1.28-1.46) | 2.08 (1.88) | 1.17 | 0.716 | 1.43 | 2.22 |

^1^converted from pooled ORs of appendix F

# Supplementary Box 4: Secondary analyses among sensitivity analyses adjusting for unmeasured confounding notes

| **Point estimate method**  In the secondary analysis, the meta-analyses with E-values and/or E-value 95% CI lower limits less than the RR_XU_ and RR_UY_ are: anxiety and medication non-adherence, opioids and medication non-adherence, depression and unprotected sex, tobacco and unprotected sex, opioids and unprotected sex, opioids and transactional sex, alcohol and multiple sexual partners, depression and multiple sexual partners, anxiety and multiple sexual partners, and tobacco and transactional sex. Bias-adjusted RRs are reported in appendix G, table 1.  **Proportion of meaningfully strong effects method**  In the secondary analysis, three meta-analyses were assessed, but only two are reported, as tobacco and multiple sexual partners did not have robust parametric confidence intervals as per the online calculator tool. Both remaining meta-analyses demonstrated moderate (stimulants and unprotected sex) or strong (stimulants and transactional sex) evidence of an association. To consider the possibility that the true association may be in the opposite direction, we repeated analyses, altering q (to RR 0.90 as all eligible meta-analyses have pooled RRs > 1). For all presented studies, Ĝ(r,q) and T̂(r,q) exceed their respective thresholds indicating robustness to unmeasured confounding. |
| --- |

# Table S4: Measures of association and E-values of exposure and outcome associations with comparable covariable distrust in medical institutions to gauge strength of the E-value

| **Association** | **Association group** | **RR**  **(95% CI)^1^** | **E-value**  **(95% CI LL)^2^** | **PICO^3^** | **Notes** | **Reference** |
| --- | --- | --- | --- | --- | --- | --- |
| Exposure-covariable | [Alcohol, tobacco, opioids, or stimulants] and [distrust in medical institutions]  (RR_XU_^ATOS-distrust^) | Men:  1.03  (0.99-1.06)  women:  1.22  (1.16-1.28) | Men:  1.20 (1.11)  Women:  1.73 (1.58) | General Swedish population; very low (lack of trust in 6–10 institutions); very high (high trust in all institutions) with four categories in total; harmful alcohol consumption (AUDIT-C with cutoff of 5 for men and 4 for women). | Reverse direction of association: alcohol was the outcome; the exposure of institutional trust included trust in medical institutions. | Ahnquist, J., Lindström, M., & Wamala, S. P. (2008). Institutional trust and alcohol consumption in Sweden: The Swedish National Public Health Survey 2006. BMC Public Health, 8(1), 283. https://doi.org/10.1186/1471-2458-8-283 |
|  | [Depression, anxiety, or pain] and [distrust in medical institutions]  (RR_XU_^DAP-distrust^) | 2.01  (1.89-2.12) | 3.43 (3.19) | Veterans Affairs health systems patients with diabetes; low physician trust (primary care assessment survey); high physician trust; non-cost related underuse of medications (taking less than prescribed, self-report). |  | Piette, J. D., Heisler, M., Krein, S., & Kerr, E. A. (2005). The Role of Patient-Physician Trust in Moderating Medication Nonadherence Due to Cost Pressures. Archives of Internal Medicine, 165(15), 1749–1755. https://doi.org/10.1001/archinte.165.15.1749 |
| Covariable-outcome | [Distrust in medical institutions] and [medication non-adherence]  (RR_UY_^distrust-medication non-adherence^) | Source 1:  1.03  (1.00-1.05)  Source 2:  1.93  (1.71-2.14) | Source 1:  1.20 (1.00)  Source 2:  3.26 (2.82) | Southern African American men with hypertension, general trust in the medical system (Hall Trust Scale); no general trust in the medical system; medication adherence (Morisky scale).  Veterans Affairs health systems patients with diabetes; low physician trust (primary care assessment survey); high physician trust; non-cost related underuse of medications (taking less than prescribed, self-report). |  | Elder, K., Ramamonjiarivelo, Z., Wiltshire, J., Piper, C., Horn, W. S., Gilbert, K. L., Hullett, S., & Allison, J. (2012). Trust, Medication Adherence, and Hypertension Control in Southern African American Men. American Journal of Public Health, 102(12), 2242–2245. https://doi.org/10.2105/AJPH.2012.300777  Piette, J. D., Heisler, M., Krein, S., & Kerr, E. A. (2005). The Role of Patient-Physician Trust in Moderating Medication Nonadherence Due to Cost Pressures. Archives of Internal Medicine, 165(15), 1749–1755. https://doi.org/10.1001/archinte.165.15.1749 |
|  | [Distrust in medical institutions] and [unprotected sex or transactional sex or multiple sexual partners]  (RR_UY_^distrust-risky sexual behavior^) | 1.09  (1.02-1.17) | 1.41 (1.16) | HIV-negative Black MSM; global medical mistrust (using the Medical Mistrust Index); unspecified score of 1-4; time since last medical exam. | Proxy for outcome: time since last medical exam. | Eaton, L. A., Driffin, D. D., Kegler, C., Smith, H., Conway-Washington, C., White, D., & Cherry, C. (2015). The Role of Stigma and Medical Mistrust in the Routine Health Care Engagement of Black Men Who Have Sex With Men. American Journal of Public Health, 105(2), e75–e82. <https://doi.org/10.2105/AJPH.2014.302322> |

^1^calculated from study data reporting ORs

^2^calculated from study data

^3^population, intervention/exposure, control/reference group, outcome

# Supplementary Box 5: Assessing Ĝ(r,q) in the context of reference risk ratios

| Comparing our E-values to these exposure-covariable (RR_XU_^ATOS-DIM^ or RR_XU_^DAP-DIM^) and covariable-outcome (RR_XU_^DIM-medication non-adherence^ or RR_XU_^DIM-risky sexual behavior^) RRs (56–59), we see that DIM appears to influence the associations less than risk propensity. Relative to DIM for risky sexual behavior outcomes, ATOS associations are uniformly “very strong” to unmeasured confounding (relative to both “exposure-covariable” and “covariable-outcome” RRs), however DAP associations are both “likely not strong” (relative to “exposure-covariable” RRs) and “very strong” to unmeasured confounding (relative to covariable-outcome” RRs). Relative to DIM for medication non-adherence, all exposures except for pain are “very strong” and/or “moderately strong” compared to “exposure-covariable” and “covariable-outcome” RRs, while pain is “likely not strong” (relative to the “exposure-covariable” RR) or “moderately strong” (relative to the “covariable-outcome RR). We used the same definitions for strength to unmeasured confounding as in the primary analysis: we describe E-values that exceed reference RRs as “very strong,” those within the range of reference RRs as “moderately strong,” and those less than reference RRs as “likely not strong” to unmeasured confounding. |
| --- |

#

#

#

# Table S5: Bias factors from associations with comparable covariable distrust in medical institutions to gauge strength of T̂(r,q)

| **Association group** | **RR_XU_** | **RR_UY_** | **Bias factor** | **Reference(s) (RR_XU_)** | **Reference(s) (RR_UY_)** |
| --- | --- | --- | --- | --- | --- |
| ATOS-non-adherence | 1.13 | 1.48 | 1.04 | Ahnquist, J., Lindström, M., & Wamala, S. P. (2008). Institutional trust and alcohol consumption in Sweden: The Swedish National Public Health Survey 2006. BMC Public Health, 8(1), 283. <https://doi.org/10.1186/1471-2458-8-283>  (Used midrange of men and women.) | Elder, K., Ramamonjiarivelo, Z., Wiltshire, J., Piper, C., Horn, W. S., Gilbert, K. L., Hullett, S., & Allison, J. (2012). Trust, Medication Adherence, and Hypertension Control in Southern African American Men. American Journal of Public Health, 102(12), 2242–2245. https://doi.org/10.2105/AJPH.2012.300777  (Used midrange of two sources.)  Piette, J. D., Heisler, M., Krein, S., & Kerr, E. A. (2005). The Role of Patient-Physician Trust in Moderating Medication Nonadherence Due to Cost Pressures. Archives of Internal Medicine, 165(15), 1749–1755. https://doi.org/10.1001/archinte.165.15.1749 |
| DAP-non-adherence | 2.01 | 1.48 | 1.19 | Piette, J. D., Heisler, M., Krein, S., & Kerr, E. A. (2005). The Role of Patient-Physician Trust in Moderating Medication Nonadherence Due to Cost Pressures. Archives of Internal Medicine, 165(15), 1749–1755. https://doi.org/10.1001/archinte.165.15.1749 | Elder, K., Ramamonjiarivelo, Z., Wiltshire, J., Piper, C., Horn, W. S., Gilbert, K. L., Hullett, S., & Allison, J. (2012). Trust, Medication Adherence, and Hypertension Control in Southern African American Men. American Journal of Public Health, 102(12), 2242–2245. https://doi.org/10.2105/AJPH.2012.300777  Piette, J. D., Heisler, M., Krein, S., & Kerr, E. A. (2005). The Role of Patient-Physician Trust in Moderating Medication Nonadherence Due to Cost Pressures. Archives of Internal Medicine, 165(15), 1749–1755. https://doi.org/10.1001/archinte.165.15.1749 |
| ATOS-risky sexual behavior | 1.13 | 1.09 | 1.01 | Ahnquist, J., Lindström, M., & Wamala, S. P. (2008). Institutional trust and alcohol consumption in Sweden: The Swedish National Public Health Survey 2006. BMC Public Health, 8(1), 283. https://doi.org/10.1186/1471-2458-8-283 | Eaton, L. A., Driffin, D. D., Kegler, C., Smith, H., Conway-Washington, C., White, D., & Cherry, C. (2015). The Role of Stigma and Medical Mistrust in the Routine Health Care Engagement of Black Men Who Have Sex With Men. American Journal of Public Health, 105(2), e75–e82. https://doi.org/10.2105/AJPH.2014.302322 |
| DAP-risky sexual behavior | 2.01 | 1.09 | 1.04 | Piette, J. D., Heisler, M., Krein, S., & Kerr, E. A. (2005). The Role of Patient-Physician Trust in Moderating Medication Nonadherence Due to Cost Pressures. Archives of Internal Medicine, 165(15), 1749–1755. https://doi.org/10.1001/archinte.165.15.1749 | Eaton, L. A., Driffin, D. D., Kegler, C., Smith, H., Conway-Washington, C., White, D., & Cherry, C. (2015). The Role of Stigma and Medical Mistrust in the Routine Health Care Engagement of Black Men Who Have Sex With Men. American Journal of Public Health, 105(2), e75–e82. https://doi.org/10.2105/AJPH.2014.302322 |
